# Productive Alkyne Metathesis with “Canopy Catalysts” Mandates Pseudorotation

**DOI:** 10.1021/jacs.1c01404

**Published:** 2021-04-07

**Authors:** Alexander Haack, Julius Hillenbrand, Markus Leutzsch, Maurice van Gastel, Frank Neese, Alois Fürstner

**Affiliations:** Max-Planck-Institut für Kohlenforschung, D-45470 Mülheim/Ruhr, Germany

## Abstract

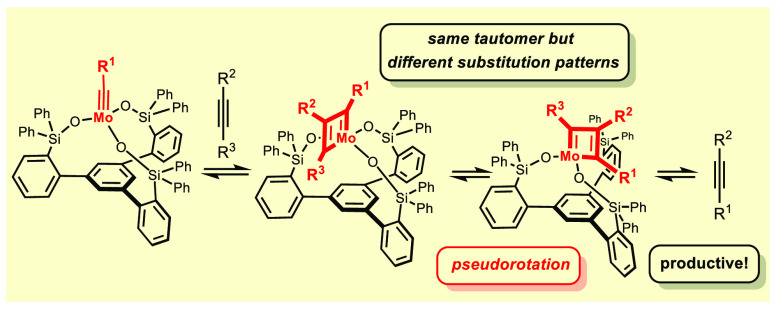

Molybdenum alkylidyne complexes of the “canopy catalyst” series define new standards in the field of alkyne metathesis. The tripodal ligand framework lowers the symmetry of the metallacyclobutadiene complex formed by [2 + 2] cycloaddition with the substrate and imposes constraints onto the productive [2 + 2] cycloreversion; pseudorotation corrects this handicap and makes catalytic turnover possible. A combined spectroscopic, crystallographic, and computational study provides insights into this unorthodox mechanism and uncovers the role that metallatetrahedrane complexes play in certain cases.

The discovery that molybdenum alkylidyne units synergize particularly well with triarylsilanolate ligands marked an important milestone in the development of alkyne metathesis in general.^1−6^ Catalysts such as **1** and the derived bench-stable phenanthroline adducts combine high activity and unrivaled functional group tolerance with a previously unknown user-friendliness (Scheme 1).^7−9^ A new generation of “canopy catalysts” of type **2** distinguished by a tripodal silanolate ligand framework shows an even better application profile.^10−13^

**Scheme 1:**
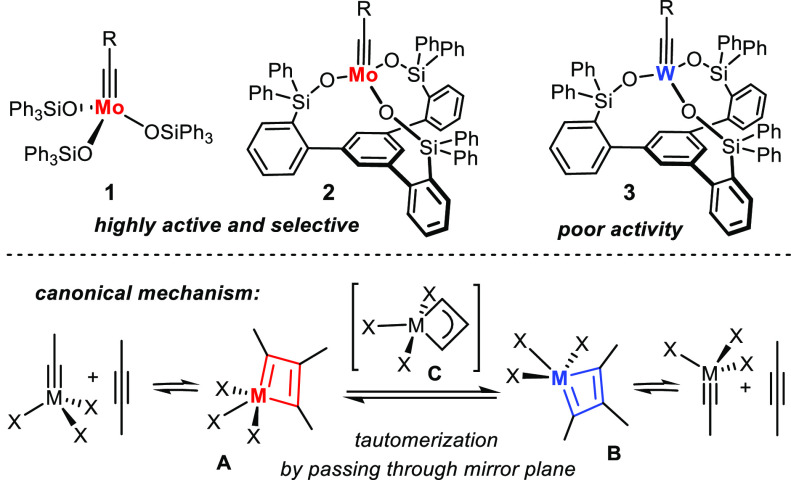
Overview

In consideration thereof, it was perplexing to find that addition of excess 3-hexyne to a solution of **2a** (R = 4-MeOC_6_H_4_-) in [D_8_]-toluene afforded the metallatetrahedrane **4** as the only detectable and isolable intermediate (Scheme 2).^10−12^ Even though its formation is reversible, as shown by exchange NMR spectroscopy (EXSY), the generally accepted mechanism of alkyne metathesis does not involve an intermediate of this type; rather, it is believed to proceed via the two square-pyramidal metallacyclobutadiene tautomers **A** and **B** formed and disassembled by [2 + 2] cycloaddition/cycloreversion; they interconvert by passing through a trigonal-bipyramidal form **C** (Scheme 1);^14−19^ metallatetrahedranes, in contrast, are considered to be unreactive sinks and/or gateways to catalyst decomposition.^20−23^ The exclusive formation of **4** from one of the best available catalysts is therefore nonintuitive.^10,11^ A veritable conundrum accrues when the behavior of the tungsten analogue **3** (R = 2,6-Me_2_C_6_H_3_-) is also taken into consideration, which furnished the canonical metallacyclobutadiene **5** on reaction with 3-hexyne. It took 1 week for the latter to transform into **6** by what represents a single “turnover”; complex **3** is hence catalytically incompetent.^24^ The question arises whether these perplexing observations challenge the consensus mechanism of alkyne metathesis or whether they can be consolidated with it. The answer is deemed critically important for further catalyst development.

**Scheme 2:**
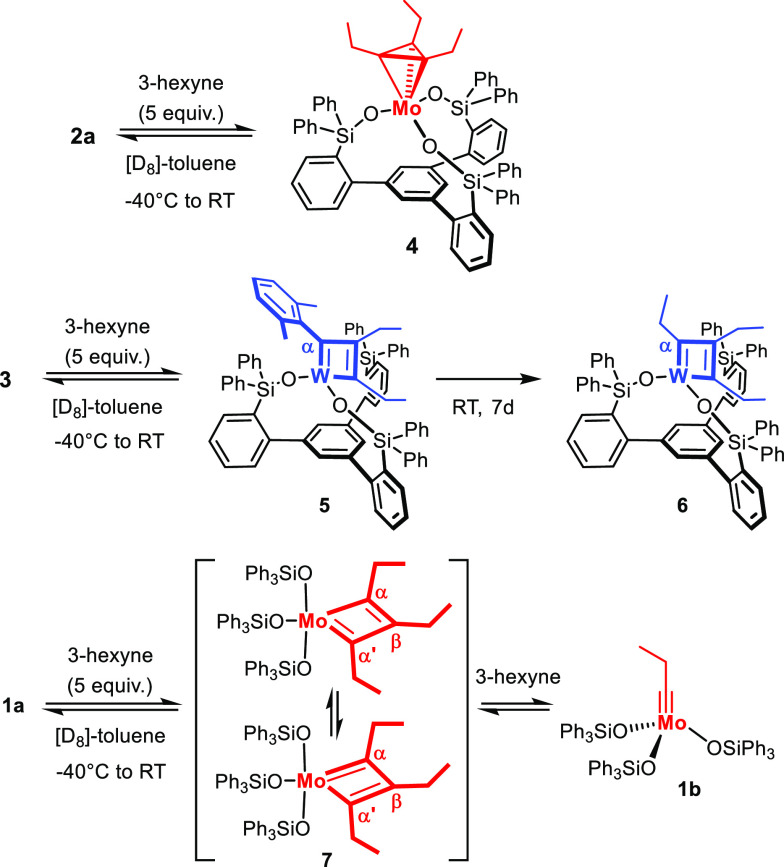
Distinct Behavior of Different Alkylidyne Complexes

In the first foray, we checked the behavior of the parent catalyst **1a** (R = 4-MeOC_6_H_4_-) endowed with *monodentate* silanolates, which had so far been tacitly assumed to follow the canonical mechanistic course. Indeed, treatment of a solution of **1a** with 3-hexyne (5 equiv) in [D_8_]-toluene gave molybdenacyclobutadiene **7** exclusively (Scheme 2). Although **1a** and **2a** are both excellent catalysts and both carry silanolate ligands, they obviously afford distinct types of intermediates on reaction with the substrate. Complex **7** is *C*_2*v*_ symmetric in solution since only one signal is observed for the two C_α_-atoms (δ_C_ = 248.8 ppm); even at −90 °C, the two tautomers of the metallacycle are not frozen out, which indicates an extremely low barrier for interconversion. EXSY-NMR data revealed the dynamic exchange of the ethyl substituents at the C_α_- and C_β_-atoms with free 3-hexyne, thus implying that the product-forming (“productive”) and the substrate-regenerating (“unproductive”) [2 + 2] cycloreversions are equally likely.^25^

Highly sensitive steel-blue crystals suitable for X-ray diffraction could be grown from a solution of **7** in Et_2_O at −85 °C. This result is deemed rewarding since pertinent information about the structure of molybdenacyclobutadienes in the solid state is very limited.^19,26−28^

The Mo(+6) center of **7** adopts a coordination geometry in between trigonal-bipyramidal and square-pyramidal (τ_5_ = 0.37, Figure 1).^29^ The bond lengths are uneven: whereas the Mo1–C2 bond is only slightly shorter than the Mo1–C3 bond, the difference is more pronounced for C1–C2 versus C1–C3 (Figure 2).^30,31^ It is remarkable that the metallacyclobutadiene forms **A**/**B** surface in the X-ray structure of **7** even though it is fairly close to the trigonal-bipyramidal rendition **C** where the tautomers converge (Scheme 1);^18^ this peculiar situation may explain why their interconversion in solution is fast even at −90 °C as manifested in the spectra of *C*_2*v*_ symmetry.^32,33^

**Figure 1:**
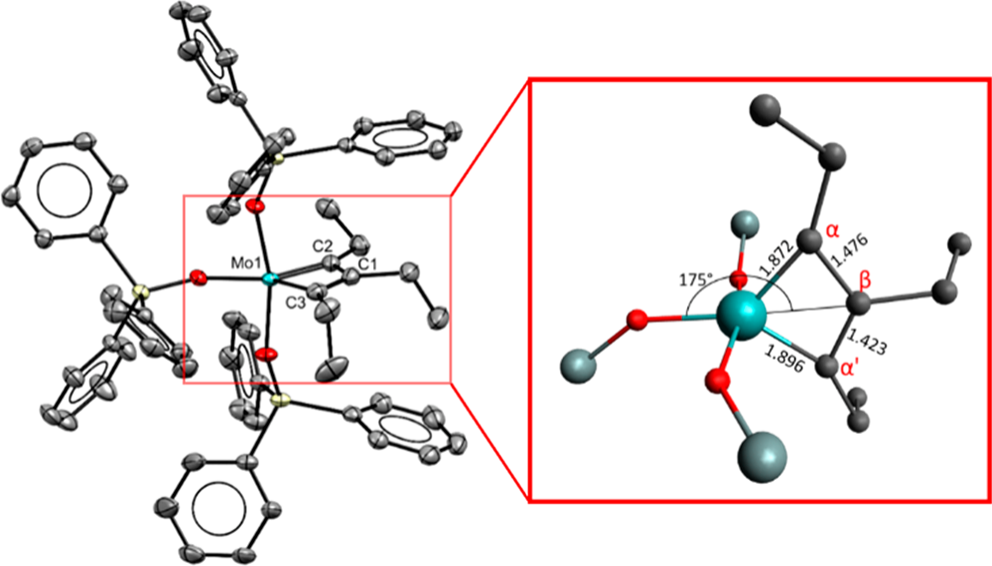
Structure of complex **7** in the solid state; H atoms and the solvent are omitted for clarity.

**Figure 2:**
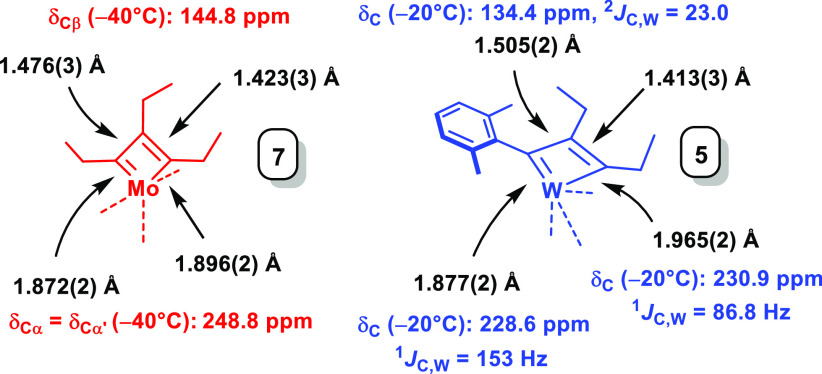
Comparison of the metallacyclobutadiene cores of complexes **7** and **5**.

The comparison of **7** with the structure of tungstenacyclobutadiene **5**(24) derived from the catalytically incompetent tungsten alkylidyne **3** is also informative, as it allows the effect of the tripodal “canopy” ligand architecture to be assessed (Figure 2). In contrast to **7**, complex **5** is closer to square-pyramidal than trigonal-bipyramidal (τ_5_ ≈ 0.14).^29^ The metallacyclic core is much more distorted in all bond distances;^24,34^ this distortion persists in solution in that the C_α_/C_α′_-atoms of **5** are inequivalent as manifested in discrete shifts and notably different ^1^*J*_C,W_ coupling constants indicative of substantial “double” bond character for the short W–C_α_ bond but “single” bond character for the longer W–C_α′_.^24^ The fact that a single tautomer of **5** is detected in solution explains why EXSY-NMR experiments show only the dynamic exchange between the ethyl substituents at C_α′_ and C_β_ with free 3-hexyne by “unproductive” [2 + 2] cycloreversion that regenerates the starting materials. The obviously much higher barrier of the “productive” cycloreversion is in line with the overly long reaction time of 1 week for **5** to transform into the all-ethyl-substituted tungstenacyclobutadiene **6**.^24^ The core of **6** must be similarly distorted since the C_α_/C_α′_-atoms and their ethyl substituents are inequivalent. However, mutual interconversion of these positions is observed on the NMR time scale: for favorable circumstances, the activation parameters could be deduced.^35^

As mentioned above, the reaction of the molybdenum alkylidyne **2** with 3-hexyne gave metallatetrahedrane **4** exclusively. A more systematic study, however, showed that the outcome is substrate-dependent: thus, treatment of **2a** with 2-butyne gave a mixture of metallatetrahedrane **8** and the corresponding metallacyclobutadiene **9** (Scheme 3).^36^ Only for the latter, a dynamic exchange with 2-butyne by [2 + 2] cycloreversion was observed by EXSY-NMR, whereas the metallatetrahedrane **8** is static at −40 °C. The mixture had to be warmed to 0 °C for **8** and **9** to mutually interconvert and for **8** to commence exchanging with 2-butyne (see the SI).

**Scheme 3:**
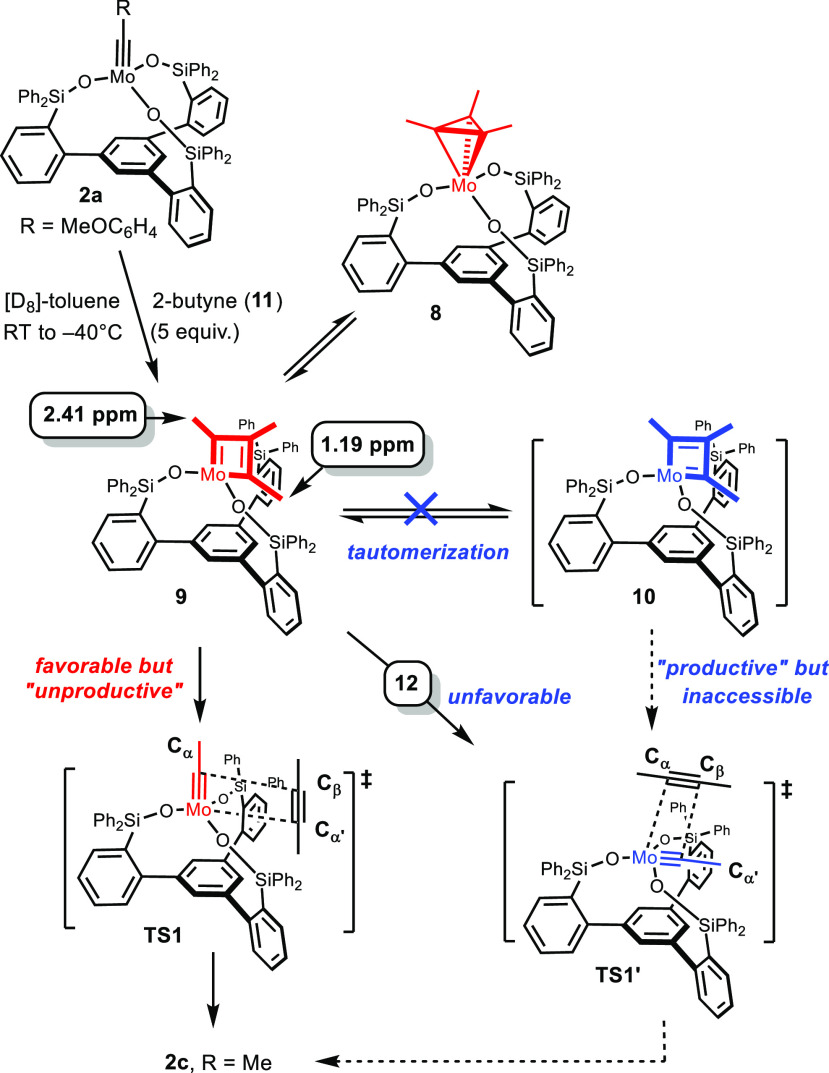
Formation and Fate of Intermediates Carrying a Tripodal Ligand Framework

The shifts of the methyl groups at the C_α_ and C_α′_ positions of **9** are strikingly different, which implies a complex of low symmetry: on the NMR time scale, only one of these methyl substituents resides in the anisotropy cone of a neighboring phenyl ring.^39,40^ Equally informative are the EXSY data, which show two different dynamic processes: First, the methyl groups at C_α′_/C_β_ exchange with 2-butyne (**11**) much more readily than that at C_α_ (Figure 3). This finding proves that the “unproductive” and the “productive” [2 + 2] cycloreversion both proceed even at −40 °C but are not equally facile.^25^ Second, interconversion of the methyl substituents at the C_α_/C_α′_ positions is observed: this effect, however, is unlikely to be caused by formation of the second canonical tautomer: the tripodal ligand scaffold renders the second canonical tautomer (Scheme 1) inaccessible on steric grounds. Retention of the geometry of **9** but shuffling of the π-bonds with formation of a hypothetical tautomer **10** is equally excluded;^41^ even if **10** were reached, release of the product would be strongly disfavored by the clash of the incipient alkylidyne with the ligand framework (Scheme 3). DFT calculations confirmed the notion of two massively different barriers for the disintegration of the metallacyclobutadiene (TS1/TS1′, see below). It is therefore safe to conclude that canopy catalysts do *not* operate by the generally accepted mechanism because the second required canonical metallacyclobutadiene tautomer is beyond reach, and its productive deconvolution is disfavored. Yet, complexes of type **2** are very powerful catalysts; therefore, some process must be operative that corrects this situation and renders turnover facile.

**Figure 3:**
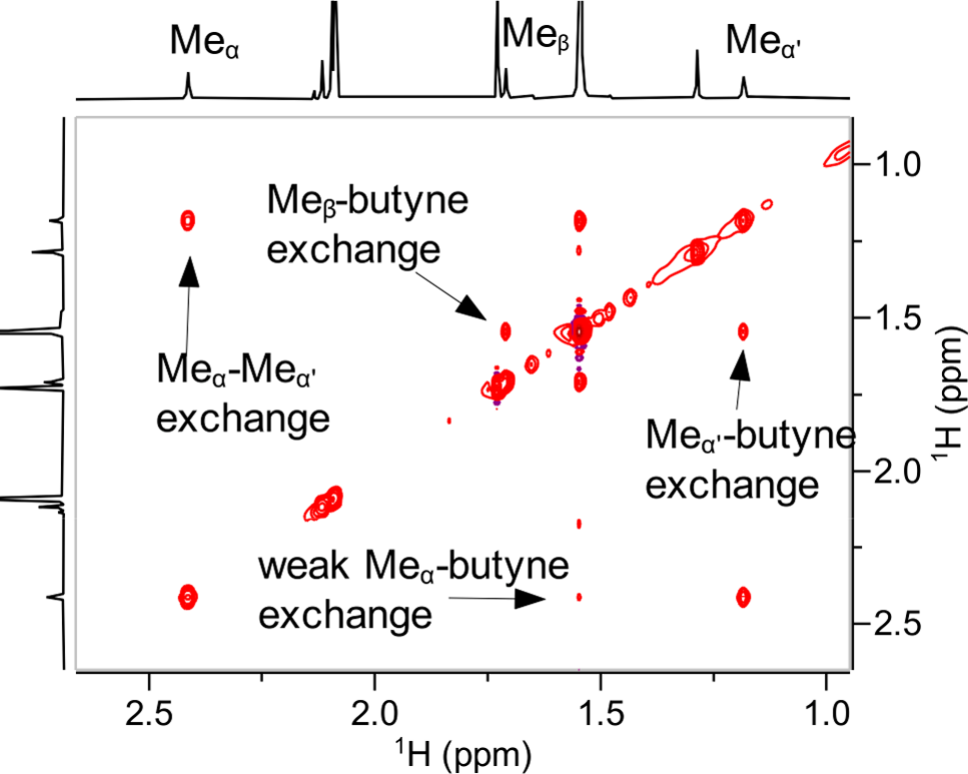
Dynamic exchange processes of **9** manifested in cross peaks in the EASY-ROESY spectrum ([D_8_]-toluene, −40 °C, spin-lock time: 200 ms).^37,38^

DFT calculations were used to probe this missing piece of the mechanism.^42,43^ The minimum and transition state geometries as well as the obtained minimum energy pathways for the reaction of **2c** (R = Me) with 2-butyne and the interplay of **8** and **9** are available in the SI as well as Cartesian coordinates and video files A and B. Figure 4 summarizes the essentials: focusing on the black data first, all barriers along the path are thermally accessible, including the interconversion of **9** and **8**. Moreover, the Gibbs free energy of the dissociated reactants is similar to that of these intermediates. Therefore, a mixture of both intermediates should be formed in the presence of excess alkyne, whereas the starting alkylidyne complex gets depleted. This conclusion is in excellent agreement with experiment (NMR) and hence gives confidence in the accuracy of the chosen DFT level of theory.

**Figure 4:**
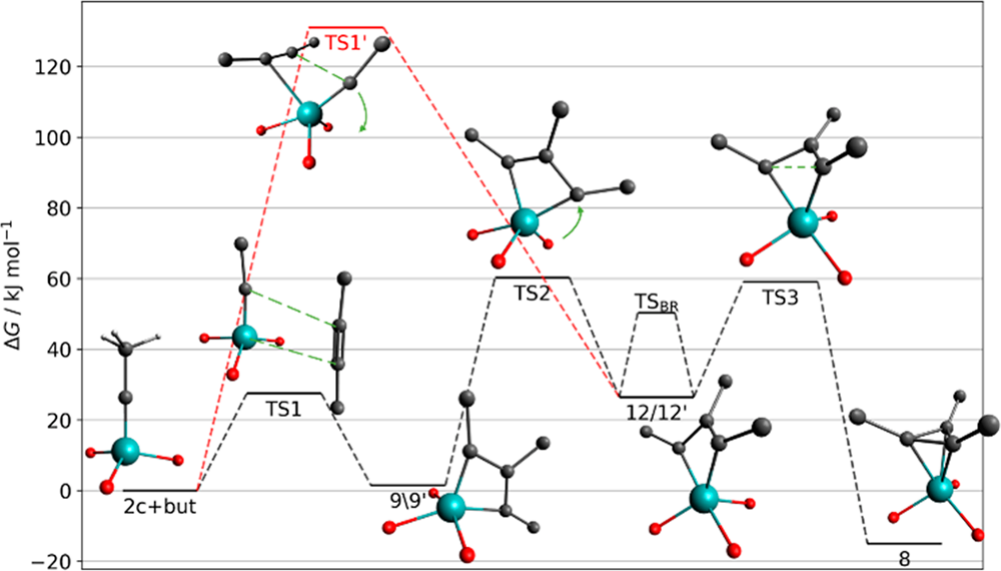
Thermochemistry of the reaction of **2c** with 2-butyne.

Complexes **9** to **8** were computationally found to interconvert via an intermediate **12**, which is higher in energy and hence not observed by NMR. A priori, **12** shows the proper π-bonding for productive cycloreversion. However, the metallacyclobutadiene ring is no longer flat as in **9**, but the three Mo–C distances are not yet equal as in **8** (Figure 5); it adopts a trigonal-bipyramidal geometry with two oxygen atoms and the former C_α_-atom occupying equatorial positions, whereas the third oxygen and the former C_α′_ are axially disposed. Related metallacyclobutadienes are known in the literature;^19^ the arguably most relevant one is a rhenacycle, in which C_β_ is tilted out of the M–C_α_–C_α′_ plane by no less than 34°; importantly, however, this complex does not undergo [2 + 2] cycloreversion and is hence catalytically incompetent.^44,45^

**Figure 5:**
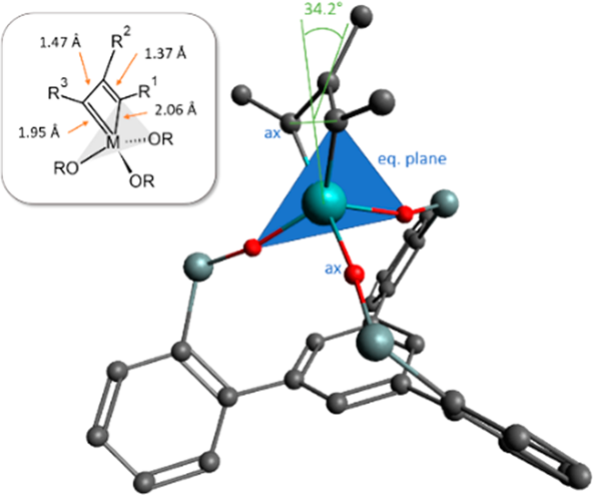
Computed structure of **12**; lateral phenyl groups and H-atoms are omitted for clarity.

Intermediate **12** is not static but succumbs to Berry pseudorotation^46^ about the adjacent M–O bond,^47^ which exchanges the axial and equatorial C_α_ positions via TS_BR_; **12**/**12′**, in turn, connect to two distinct metallacyclobutadienes **9**/**9′**, in which the C_α_/C_α′_ atoms and their substituents R^1^/R^3^ are mutually exchanged, whereas C_β_ remains in place (Scheme 4).^48,49^

**Scheme 4:**
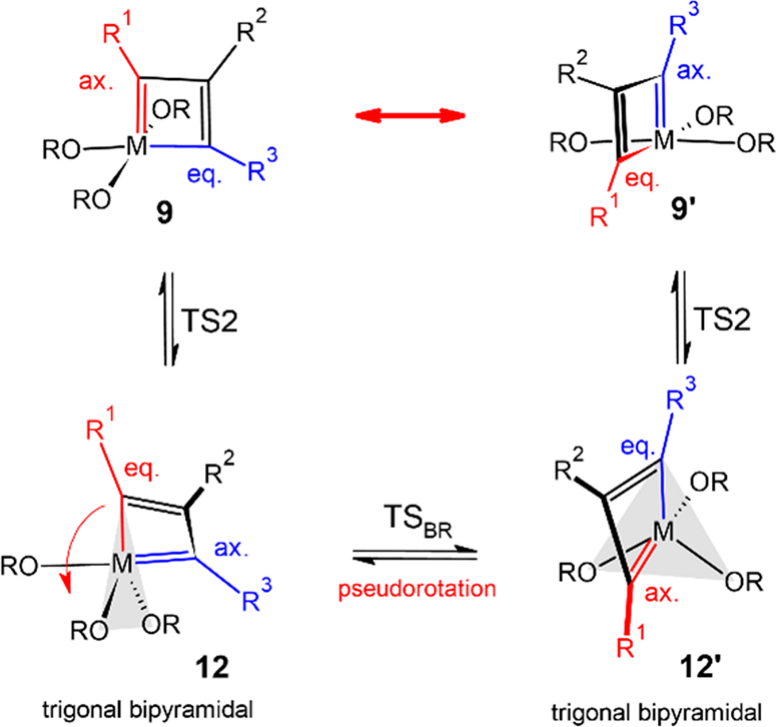
Crucial Berry Pseudorotation

Because of the lost *C*_2*v*_ symmetry, only the “unproductive” cycloreversion is facile for metallacyclobutadiene **9** via the low-lying TS1.^50,51^ “Productive” cleavage would either require isomer **10**, which is not within reach, or the highly distorted metallacycle **12**, for which DFT predicts an unfavorably high barrier (TS1′) (Figure 4). The fact that **9** is, after all, not a dead end but a truly competent catalytic intermediate is solely due to its dynamic behavior: the pseudorotation that interconverts **9**/**9′** via **12**/**12′***entails exchange of the R*^1^*and R*^3^*substituents on one and the same tautomeric form of the* π-*system* (Scheme 5). The small barrier TS_BR_ can be overcome at (or even below) room temperature, where the canopy catalysts are usually fully operative. Hence, we conclude that catalysts of type **2** operate by an unprecedented mechanism that involves *a single tautomeric form of the metallacyclobutadiene which appears in two differently substituted formats* (**9**/**9′**). Pseudorotation is the quintessential link in between them, without which product formation and catalyst turnover would not take place. The need to pass through this higher-lying intermediate and the accumulation of **8** off the actual cycle (see below) might be construed as an inherent kinetic disadvantage: indeed, **2** reacts more slowly than **1**. Importantly, however, canopy catalysts comprising smaller lateral R_2_Si– groups allow this handicap to be counterbalanced.^10^

**Scheme 5:**
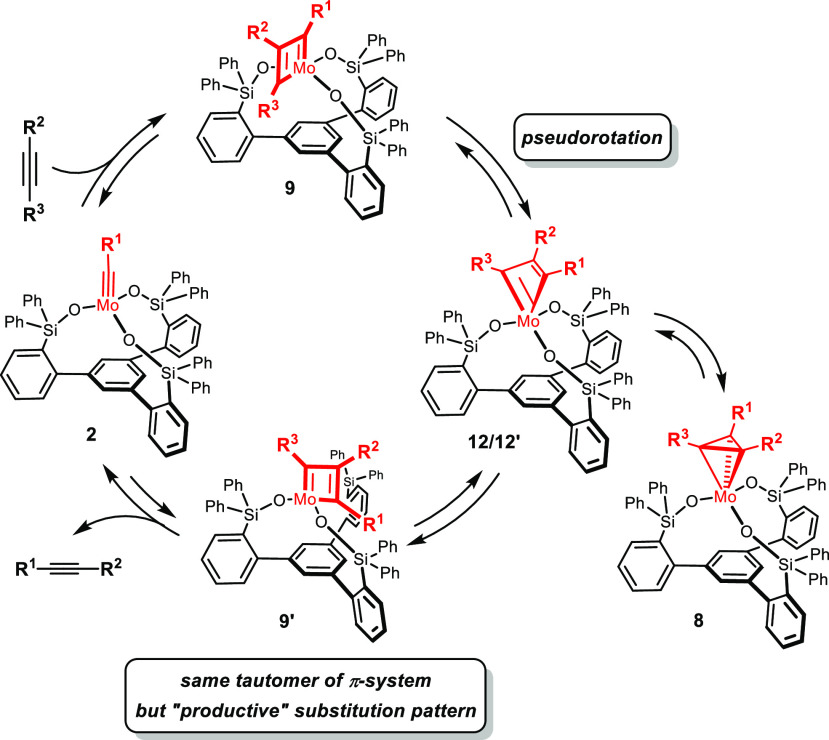
Turnover Enabled by Pseudorotation

Finally, one needs to consider that the interconversion of **9** and **9′** could pass through **8**. Yet, several pieces of evidence speak against this assumption. As discussed above, EXSY-NMR experiments showed the exchange of **9** with 2-butyne at −40 °C, whereas **8** was static; productive and unproductive [2 + 2] cycloreversions are obviously ongoing, but the metallatetrahedrane is not engaged. The new mechanism allows this observation to be readily explained, since the barrier TS_BR_ for pseudorotation is lower than TS3 connecting **9** and **8**.

Moreover, if a metallatetrahedrane were to connect **9**/**9′**, *all three* C atoms would eventually get scrambled. However, the EXSY-NMR experiments showed only exchange of C_α_/C_α′_ but no exchange of C_α_/C_β_. For the tungstenacyclobutadiene **6**, which exhibits an analogous dynamic behavior, such a process can also be firmly excluded: only the NMR signals of the C_α_/R^1^ and C_α′_/R^3^ are broadened, whereas the resonances of C_β_/R^2^ remain sharp.

Taken together, these data suggest that the metallatetrahedrane is off-cycle (Scheme 5). The question as to whether this conclusion applies to any substrate/catalyst combination can currently not be answered. In the present case, however, it is clear that intermediate **12** brokers the interconversion of the metallacyclobutadiene isomers **9**/**9′** and connects them with the metallatetrahedrane **8**; since TS2 and TS3 are of similar magnitude, a metallatetrahedrane can–but must not–be present in high concentration.

In summary, a combined spectroscopic/theoretical investigation advocates the notion that the performant canopy catalysts for alkyne metathesis operate by a mechanism that is notably different from that of earlier catalyst generations. The tripodal ligand framework lifts the degeneracy of the [2 + 2] cycloreversions and makes the classical pathway unattainable: pseudorotation, however, clears this handicap. This conclusion needs to be closely considered in future catalyst development exercises.

## Supporting Information Available

Experimental part (procedures, characterization data, and supporting crystallographic information) and computational part (energies and geometries of stable structures computed as well as energy paths of isomerization reactions) (PDF)
Pseudorotation video A (ZIP)
Pseudorotation video B (ZIP)

## Accession Codes

CCDC 1987916 contains the supplementary crystallographic data for this paper. These data can be obtained free of charge via www.ccdc.cam.ac.uk/data_request/cif, or by emailing data_request@ccdc.cam.ac.uk, or by contacting The Cambridge Crystallographic Data Centre, 12 Union Road, Cambridge CB2 1EZ, UK; fax: +44 1223 336033.
