# Supplementary material for: Productive Alkyne Metathesis with “Canopy Catalysts” Mandates Pseudorotation
Source: J Am Chem Soc. 2021 Apr 7;143(15):5643–8. doi: 10.1021/jacs.1c01404 (PMC8154524; doi:10.1021/jacs.1c01404)
Supplement: Supplementary file 1 — ja1c01404_si_001.pdf [file ja1c01404_si_001.pdf]

SUPPORTING INFORMATION

Productive Alkyne Metathesis with "Canopy Catalysts" Mandates Pseudorotation

Alexander Haack§, Julius Hillenbrand§, Markus Leutzsch, Maurice van Gastel, Frank Neese*, and Alois Fürstner*

§*these authors contributed equally*

Max-Planck-Institut für Kohlenforschung, 45470 Mülheim/Ruhr, Germany

Table of Contents

SUPPORTING CRYSTALLOGRAPHIC INFORMATION S2

GENERAL S7

EXPERIMENTAL DATA S7

COMPUTATIONAL DATA S15

Optimized geometries S15

2-butyne S15

Ethylidyne **10** S15

TS1 .... S18

TS1' S21

MCBD **9** S24

TS2 .... S27

Intermediate **12** S29

TS_{BR} S32

TS3 .... S35

MTd **8** S38

Minimum energy paths of isomerization S42

MCBD formation S42

MCBD **9** to Intermediate **12** S43

Berry Pseudorotation **12** to **12'** S44

Intermediate **12** to MTd **8** S44

High barrier dissociation (**TS1'**) S45

NMR calculation S45

SPECTRA S46

REFERENCES S76

SUPPORTING CRYSTALLOGRAPHIC INFORMATION

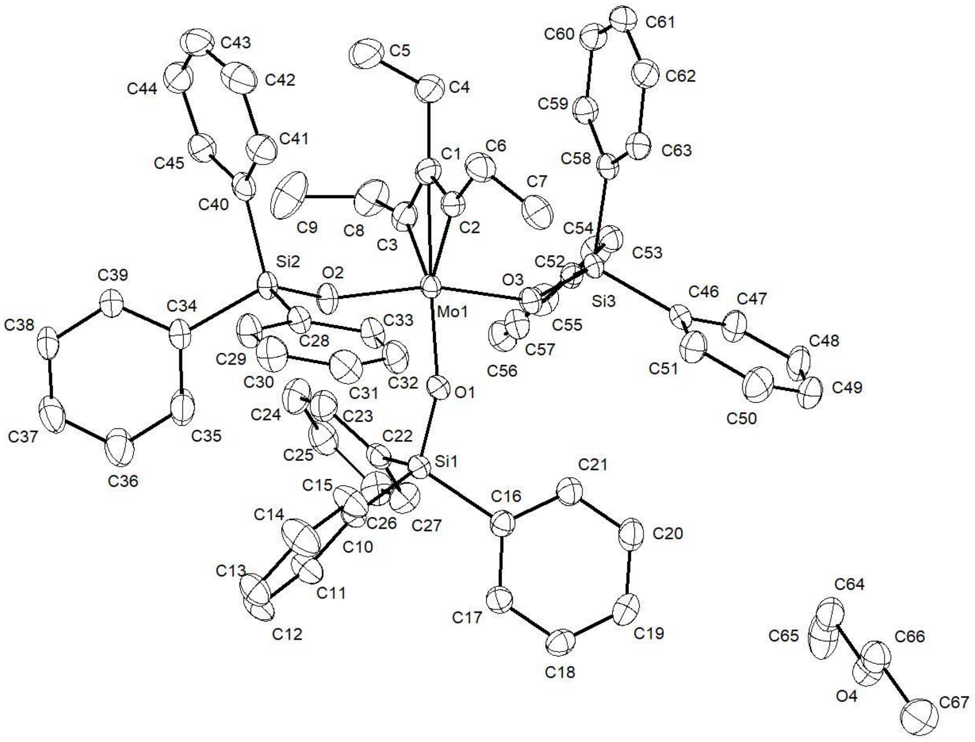

Figure S1. Structure of complex **7** in the solid state; hydrogen atoms omitted for clarity

X-ray Crystal Structure Analysis of Complex 7 (internal number 12905): C_{67} H_{70} Mo O_4 Si_3, M_r = 1119.44 g · mol^{-1}, blue prism, crystal size 0.22 x 0.16 x 0.15 mm^3, monoclinic, space group $P2_1$/n [14], a = 11.515(3) Å, b = 17.3173(16) Å, c = 29.157(4) Å, β = 94.723(11)°, V = 5794.3(16) Å^3, T = 100(2) K, Z = 4, D_{calc} = 1.283 g · cm^3, λ = 0.71073 Å, $\mu(Mo\text{-}K_\alpha)$ = 0.336 mm^{-1}, analytical absorption correction (T_{min} = 0.93, T_{max} = 0.96), Bruker AXS Enraf-Nonius KappaCCD diffractometer with a FR591 rotating Mo-anode X-ray source, 2.628 < θ< 33.193°, 125041 measured reflections, 22074 independent reflections, 15242 reflections with I > 2σ(I), R_{int} = 0.0658, 689 parameters, S = 1.064, residual electron density +0.8 (0.71 Å from Mo1) / –1.0 (0.72 Å from Mo1) e · Å^{-3}. The structure was solved by *SHELXT* and refined by full-matrix least-squares (*SHELXL*) against F^2 to R_1 = 0.048 [I > 2σ(I)], wR_2 = 0.119. **CCDC-1987916**

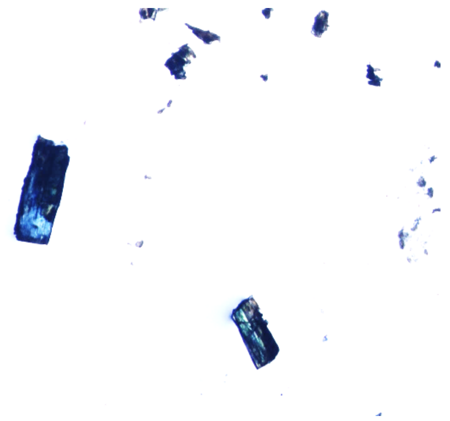

Figure S2. Photo of the steel-blue crystals of complex **7**.

There is no significant residual electron density in the crystal structure of **7** that would indicate disorder of the metallacyclobutadiene unit. The atomic displacement parameters of C1, C2 and C3 are not significantly larger than those of neighboring atoms.

A search of the Cambridge Structural Database (March 2021) for compounds containing the metallacyclobutadiene unit gave three crystal structures (DIRFOC, VIZWUZ, WEJCOG) with a comparable coordination geometry as found in complex **7 (= 12905)**; additional crystal structures show either heteroatom-substituted and/or paramagnetic molybdenacyclobutadienes (QOKPUB, GENTIF, GENTOL, MATRAE, NATREI) or deprotio-molybdenacyclobutadienes (DEGWAN, LEKHEQ, VIZXAG) that are of no immediate relevance. All of these complexes are compiled in the Table shown below.

Of these crystal structures, VIZWUZ (–OCMe$(CF_3)_2$ as catalytically competent anionic ligands) and WEJCOG (–OC$(CF_3)_3$ as anionic ligands that overstabilize the metallacycle and hence entail poor catalytic activity) are most relevant as they contain the identical 4-ethylhept-4-en-3-yl entity and a Mo central atom. The distortion of the bond angles in VIZWUZ is similar (final R-value: 8.26% and significant disorder) but less pronounced in WEJCOG (final R-value: 7.44%; no disorder). The arrangement of the two axial O atoms with respect to the equatorial plane is also worth noting: In all three cases, the two axial O-atoms are slightly bent away from the shorter of the two Mo-C bonds when the metallacycle is viewed from above. In addition to the uneven bond lengths and –angles, this observation may be taken as a further indication that the structures of these metallacycles in the solid state basically capture one of the two possible tautomeric forms of the metallacyclodiene.

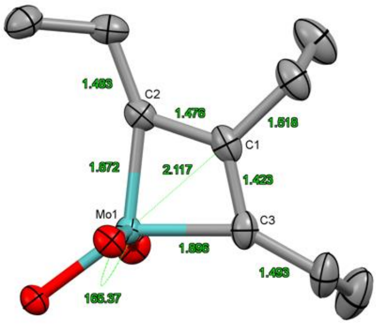

12905

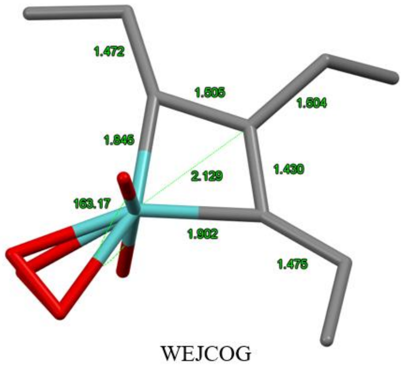
WEJCOG

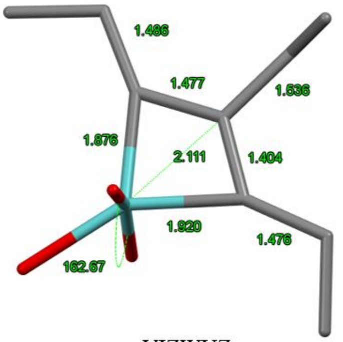
VIZWUZ

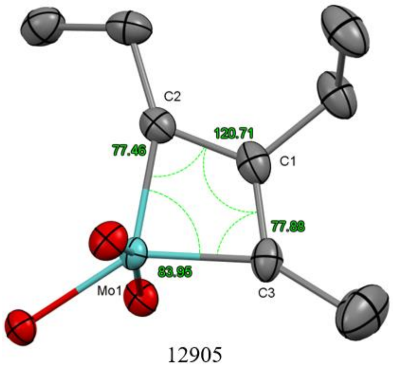

12905

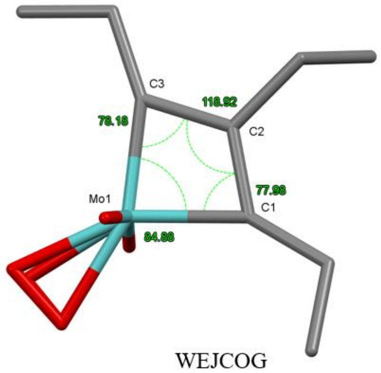

WEJCOG

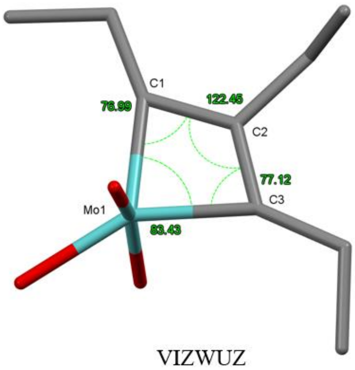

VIZWUZ

Table S1. Molybdenacyclobutadienes Deposited in the Cambridge Crystallographic Data (March 2021)

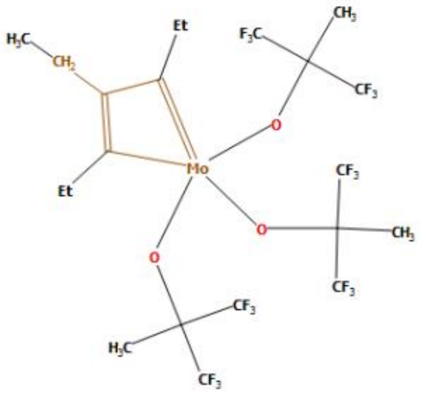

VIZWUZ
1892548

Ehrhorn, H.; Bockfeld, D.; Freytag, M.; Bannenberg, T.; Kefalidis, C. E.; Maron, C.; Tamm, M. Studies on Molybdena- and Tungstenacyclobutadiene Complexes Supported by Fluoroalkoxy Ligands as Intermediates of Alkyne Metathesis. *Organometallics* **2019**, *38*, 1627-1639.

DOI: 10.1021/acs.organomet.9b00068

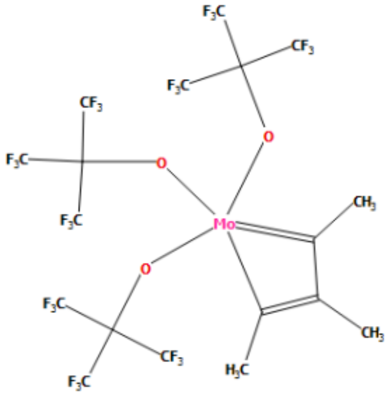

DIRFOC
1573642

Estes, D. P.; Gordon, C. P.; Fedorov, A.; Liao, W.-C.; Ehrhorn, H.; Bittner, C.; Zier, M. L.; Bockfeld, D.; Chan, K. W.; Eisenstein, O.; Raynaud, C.; Tamm, M.; Copéret, C. Molecular and Silica-Supported Molybdenum Alkyne Metathesis Catalysts: Influence of Electrons and Dynamics on Activity Revealed by Kinetics, Solid-State NMR, and Chemical Shift Analysis. *J. Am. Chem. Soc*. **2017**, *139*, 17597-17607.

DOI: 10.1021/jacs.7b09934

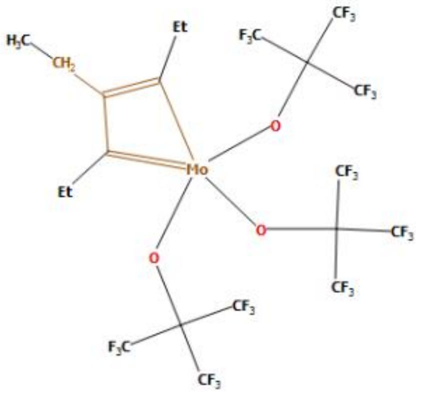

WEJCOG
1566270

Bittner, C.; Ehrhorn, H.; Bockfeld, D.; Brandhorst, K.; Tamm, M. Tuning the Catalytic Alkyne Metathesis Activity of Molybdenum and Tungsten 2,4,6-Trimethylbenzylidyne Complexes with Fluoroalkoxide Ligands $OC(CF_3)_nMe_{3-n}$ (n = 0-3). *Organometallics* **2017**, *36*, 3398–3406.

10.1021/acs.organomet.7b00519

DEPROTIO-MOLKYBDENACYCLOBUTADIENES

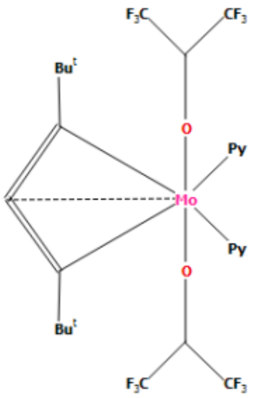

DEGWAN
1138179

McCullough, L. G.; Schrock, R. R.; Dewan, J. C.; Murdzek, J. C., Preparation of Trialkoxymolybdenum(VI)Alkyxlidyne Complexes, Their Reactions with Acetylenes, and an X-ray Strcuture of $Mo[C_3(CMe_3)_2][OCH(CF_3)_2]_2(C_5H_5N)_2$, J. Am. Chem. Soc. **1985**, *107*, 5987-5998.

DOI: 10.1021/ja00307a025

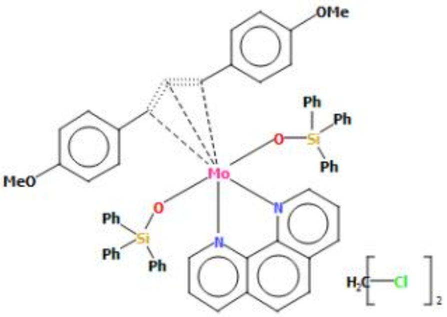

LEKHEQ
867604

Heppekausen, J.; Stade, R.; Kondoh, A.; Seidel, G.; Goddard, R.; Fürstner, A. Optimized Synthesis, Structural Investigations, Ligand Tuning and Synthetic Evaluation of Silyloxy-based Alkyne Metathesis Catalysts. *Chem. Eur. J.* **2012**, *18*, 10281-10299.

DOI: 10.1002/chem.201200621

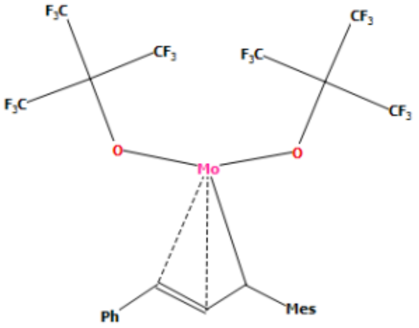

VIZXAG
1892549

Ehrhorn, H.; Bockfeld, D.; Freytag, M.; Bannenberg, T.; Kefalidis, C. E.; Maron, C.; Tamm, M. Studies on Molybdena- and Tungstenacyclobutadiene Complexes Supported by Fluoroalkoxy Ligands as Intermediates of Alkyne Metathesis. *Organometallics* **2019**, *38*, 1627-1639.

DOI: 10.1021/acs.organomet.9b00068

MISCELLANEOUS

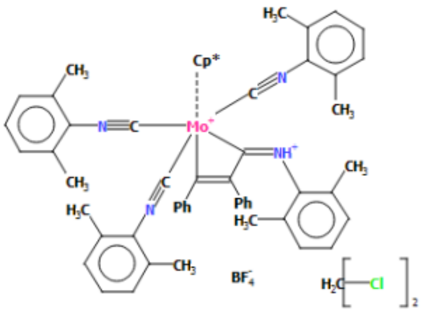

QOKPUB
155029

Adams, C. J.; Anderson, K.M.; Bartlett, I.M.; Connelly, N.G.; Orpen, A.G.; Paget, T.J.; Phetmung, H.; Smith, D.W., Molybdenum-based Alkyne-Isocyanide Coupling Reactions: Synthesis of a Reactive Diaminometallacyclopentene Complex, *J. Chem. Soc., Dalton Trans.*, **2001**, 1284-1292.

DOI: 10.1039/b010018h

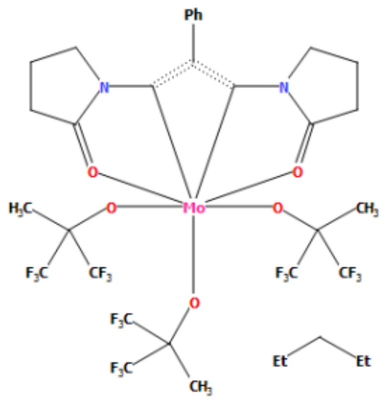

GENTIF
1570921

Jeong, H.; von Kugelgen, S.; Bellone, D.; Fischer, F. R., Regioselective Termination Reagents for Ring-Opening Alkyne Metathesis Polymerization, *J. Am. Chem. Soc.*, **2017**, *139*, 15509-15514.

DOI: 10.1021/jacs.7b09390

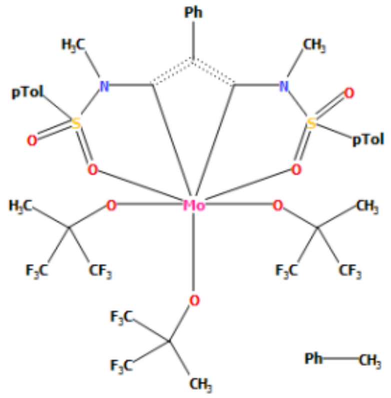

GENTOL
1570922

Jeong, H.; von Kugelgen, S.; Bellone, D.; Fischer, F. R., Regioselective Termination Reagents for Ring-Opening Alkyne Metathesis Polymerization, *J. Am. Chem. Soc.*, **2017**, *139*, 15509-15514.

DOI: 10.1021/jacs.7b09390

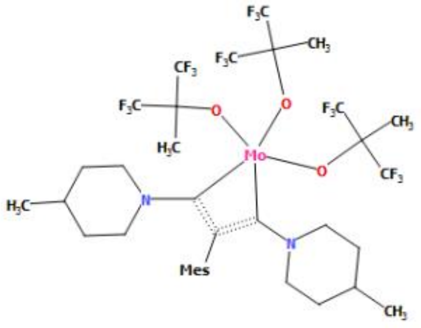

NATRAE
1499336

Arias, O.; Brandhorst, K.; Baabe, D.; Freytag, M.; Jones, P. G.; Tamm, M., Formation of Paramagnetic Metallacyclobutadienes by Reaction of Diaminoacetylenes with Molybdenum Alkylidyne Complexes, *Dalton Trans.* **2017**, *46*, 4737-4748.

DOI: 10.1039/C7DT00305F

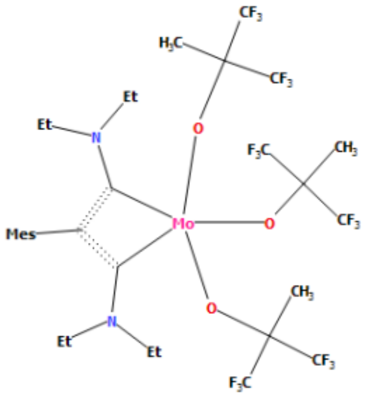

NATREI
1499337

Arias, O.; Brandhorst, K.; Baabe, D.; Freytag, M.; Jones, P. G.; Tamm, M., Formation of Paramagnetic Metallacyclobutadienes by Reaction of Diaminoacetylenes with Molybdenum Alkylidyne Complexes, *Dalton Trans.* **2017**, *46*, 4737-4748.

DOI: 10.1039/C7DT00305F

GENERAL

Unless stated otherwise, all reactions were carried out under Ar in flame-dried glassware. The solvents and commercially available compounds (Aldrich) were purified by distillation over the drying agents indicated and were transferred under Ar: Et_2O (CaH_2), [D_8]-toluene (3Å MS), 2-butyne (3Å MS), and 3-hexyne (3Å MS). Complexes **1-3** were prepared as described in the literature.[1] The molecular sieves used in this investigation were dried for 24 h at 150 °C (sand bath) under vacuum prior to use and were stored and transferred under argon atmosphere.

NMR spectra were acquired on Bruker AvanceIII 300, 400, 500 MHz or an AvanceNeo 600 MHz NMR spectrometer in the solvents indicated; chemical shifts (δ) are given in ppm relative to TMS, coupling constants (*J*) in Hz. The solvent signals were used as references and the chemical shifts converted to the TMS scale ($CDCl_3$: $\delta_C \equiv 77.0$ ppm; residual $CHCl_3$ in $CDCl_3$: $\delta_H \equiv 7.26$ ppm; CD_2Cl_2: $\delta_C \equiv 53.8$ ppm; residual ^1H: $\delta_H \equiv 5.32$ ppm; [D_8]-toluene: $\delta_C \equiv 20.7$ ppm; residual $D_5C_6CD_2H$: $\delta_H = 2.09$ ppm). ^{29}Si chemical shifts were referenced indirectly to the residual ^1H chemical shift of the deuterated solvent and are reported relative to the signal of 1% Me_4Si in $CDCl_3$ ($\delta_{Si} \equiv 0.0$ ppm).[2]

EXPERIMENTAL DATA

Complex 7

A 10 mL Schlenk flask was equipped with a magnetic stir bar and was flame-dried under vacuum. The flask was flushed with argon and charged with **1a** (27.1 mg, 0.026 mmol), which was dissolved in Et_2O (1 mL) to give a yellow solution. Upon addition of 3-hexyne (10.7 mg, 14.8 µL) the color instantly changed to green/blue. This solution was filtered via cannula under Ar and the filtrate stored at –85 °C for one week to obtain very sensitive, steel-blue crystals suitable for single-crystal X-ray diffraction (Figure S2).

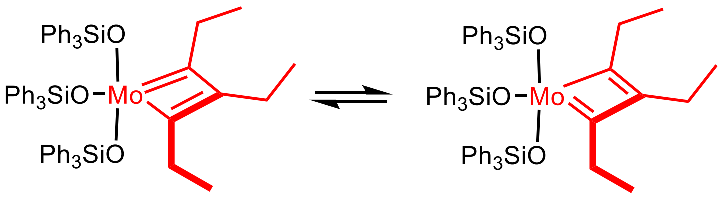

A 10 mL Schlenk flask was equipped with a magnetic stir bar and was flame-dried under vacuum. The flask was flushed with argon and charged with complex **1a** (12.6 mg, 0.012 mmol), which was dissolved in $C_6D_5CD_3$ (0.6 mL) to give a yellow solution. This solution was transferred into a *J. Young* NMR Tube and a ^1H-NMR spectrum was measured. Upon addition of 3-hexyne (10.7 mg, 6.9 µL) the color instantly changed to green/blue. Complex **7** was fully characterized in this mixture by NMR spectroscopy at –40 °C (see below for a tabular survey of the assignments). ^1H NMR (600 MHz, $C_6D_5CD_3$, -40°C): δ =

8.07 (d, *J* = 7.3 Hz, 6H), 7.45 – 7.42 (m, 12H), 7.11 (t, *J* = 7.5 Hz, 9H), 7.02 (t, *J* = 7.3 Hz, 18H), 2.61 (q, *J* = 7.5 Hz, 4H), 1.87 (q, *J* = 7.8 Hz, 2H), 1.52 (t, *J* = 7.5 Hz, 6H), -0.13 (t, *J* = 7.7 Hz, 3H). ^{13}C NMR (151 MHz, $C_6D_5CD_3$, -40°C): δ = 249.0, 145.0, 138.7, 137.5, 136.3, 135.6, 129.5, 128.8, 127.49(2C), 31.5, 26.1, 14.6, 11.4. ^{29}Si NMR (119 MHz, $C_6D_5CD_3$, -40°C): = δ –15.6, –22.3.

Complexes 8 and 9

A 10 mL Schlenk flask was equipped with a magnetic stir bar and was flame-dried under vacuum. The flask was back-filled with argon and then charged with complex [**2a**]$_2$ (13.9 mg, 12.5 µmol) and $C_6D_5CD_3$ (0.6 mL). The resulting yellow suspension was transferred into a *J. Young* NMR Tube and heated at 60°C for 1 h to give an orange solution containing only monomeric complex **2a**. Upon addition of 2-butyne (4.9 µL, 62.3 µmol) the color instantly changed to black. Complexes **8** and **9** contained in this mixture were fully characterized by NMR spectroscopy at –40 °C (for complex **8**, the NMR signals of H-14 , C11 and C14 could be extracted from the spectra of the mixture; the assignment of all other signals in tabular form is shown below): ^{1}H NMR (600 MHz, $C_6D_5CD_3$, -40°C): δ = 7.86 – 7.82 (m, 12H), 7.80 – 7.77 (m, 3H), 7.32 (s, 3H), 7.16 – 7.14 (m, 6H), 7.08 – 7.04 (m, 12H), 6.97 – 6.93 (m, 3H), 1.29 (s, 9H); ^{13}C NMR (151 MHz, $C_6D_5CD_3$, -40°C): δ = 150.7, 144.5, 137.5, 136.6, 135.7, 130.2, 130.0, 129.8, 128.4, 125.7, 78.3, 8.3. ^{29}Si NMR (119 MHz, $C_6D_5CD_3$, -40°C): = δ –8.7.

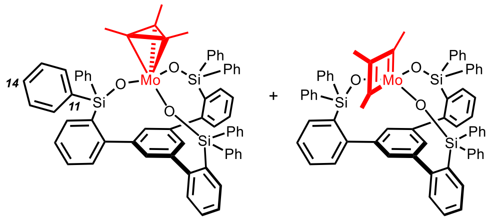

Complex **9**: ^{1}H NMR (600 MHz, $C_6D_5CD_3$, -40°C): δ = 8.35 (d, *J* = 6.7 Hz, 4H), 8.26 (s, 1H), 7.87 – 7.83 (m, 1H), 7.72 (d, *J* = 7.2 Hz, 2H), 7.58 (d, *J* = 1.2 Hz, 2H), 7.54 (d, *J* = 7.0 Hz, 4H), 7.35 (d, *J* = 8.4 Hz, 2H), 7.25 – 7.19 (m, 2H), 7.20 – 7.17 (m, 1H), 7.16 – 7.13 (m, 2H), 7.11 – 7.08 (m, 6H), 7.07 – 7.05 (m, 2H), 7.05 – 7.01 (m, 8H,), 6.97 – 6.93 (m, 4H), 6.89 (t, *J* = 7.6 Hz, 4H), 2.41 (s, 3H), 1.71 (s, 3H), 1.18 (s, 3H). ^{13}C NMR (151 MHz, $C_6D_5CD_3$, -40°C): δ = 235.7, 234.2, 148.9, 146.8, 145.0, 144.6, 143.5, 140.5, 140.4, 140.2, 140.2, 137.0, 136.0, 135.9, 135.7, 135.4, 135.2, 131.4 (3x), 129.4, 129.2, 129.0, 128.7, 127.8, 127.6, 127.6, 127.5, 126.4, 126.1, 125.2, 22.9, 21.8, 18.0. ^{29}Si NMR (119 MHz, $C_6D_5CD_3$, -40°C): = δ –19.6, –20.0.

NMR Characterization of Complex 7

The sample was a complex mixture, but the following three different species could be identified and fully characterized. Please see the next pages for the characterization data of metallacyclobutadiene **7**. There is another species containing SiPh groups that is part of this equilibrium, which could not be identified. From ^{29}Si NMR it only shows one signal indicating it is C_3 symmetric. All of this species are interconverting into each other and pseudo-cross peaks between different molecules are observed in the ROESY data. This indicated that the exchange is very quick.

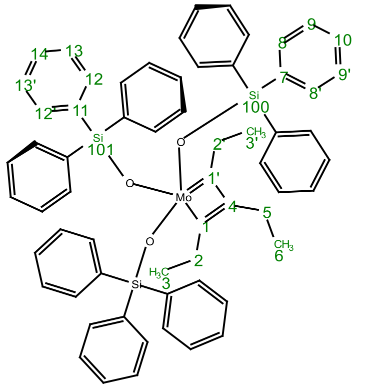

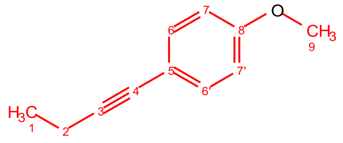

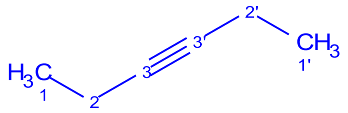

NMR Assignment: Metallacyclobutadiene Complex 7, $C_6D_5CD_3$, –40 °C

Atom	δ (ppm)	COSY	HSQC	^{13}C-HMBC	^{29}Si-HMBC	Atom	δ (ppm)	COSY	HSQC	^{13}C-HMBC	^{29}Si-HMBC
**1 C**	249.0			2, 3		**9 C**	127.5		9	9'	
**1' C**	249.0			3'		**H**	7.02	8	9	7, 9'	100
**2 C**	31.4		2	3		**9' C**	127.5		9'	9	
**H2**	2.61	3	2	1, 3, 4		**H**	7.02	9'	9'	7, 9	100
**2' C**	31.4		2'	3'		**10 C**	128.8		10	8, 8'	
**H2**	2.61	3'	2'			**H**	7.11		10	8, 8'	
**3 C**	14.6		3	2		**11 C**	137.5			12, 13, 13'	
**H3**	1.52	2	3	1, 2		**12 C**	136.3		12	14	
**3' C**	14.6		3'			**H**	8.07	13	12	11, 12', 14	101
**H3**	1.52	2'	3'	1', 2'		**12' C**	136.3		12'	12, 14	
**4 C**	145.0			2, 5, 6		**H**	8.07	13'	12'		101
**5 C**	26.1		5	6		**13 C**	127.5		13	13'	
**H2**	1.87	6	5	4, 6		**H**	7.02	12	13	11, 13'	
**6 C**	11.4		6	5		**13' C**	127.5		13'	13	
**H3**	-0.13	5	6	4, 5		**H**	7.02	12'	13'	11, 13	
**7 C**	138.7			8, 8', 9, 9'		**14 C**	129.5		14	12	
**8 C**	135.6		8	8', 10		**H**	7.11		14	12, 12'	
**H**	7.44	9	8	7, 8', 10	100	**100 Si**	-22.3				8, 8', 9, 9'
**8' C**	135.6		8'	8, 10		**101 Si**	-15.6				12, 12'
**H**	7.44		8'	7, 8, 10	100						

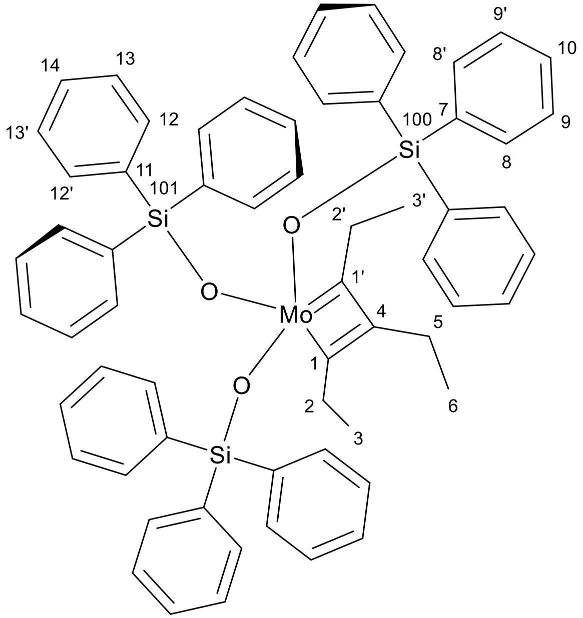

Atom	δ (ppm)	COSY	HSQC	^{13}C-HMBC	^{29}Si-HMBC	Atom	δ (ppm)	COSY	HSQC	^{13}C-HMBC	^{29}Si-HMBC
**1 C**	248.84			2, 3		**9 C**	127.3		9	9'	
**1' C**	248.84			3'		**H**	7.02	8	9	7, 9'	100
**2 C**	31.22		2	3		**9' C**	127.3		9'	9	
**H2**	2.61	3	2	1, 3, 4		**H**	7.02	9'	9'	7, 9	100
**2' C**	31.22		2'	3'		**10 C**	128.59		10	8, 8'	
**H2**	2.61	3'	2'			**H**	7.11		10	8, 8'	
**3 C**	14.36		3	2		**11 C**	137.33			12, 13, 13'	
**H3**	1.52	2	3	1, 2		**12 C**	136.14		12	14	
**3' C**	14.36		3'			**H**	8.07	13	12	11, 12', 14	101
**H3**	1.52	2'	3'	1', 2'		**12' C**	136.14		12'	12, 14	
**4 C**	144.79			2, 5, 6		**H**	8.07	13'	12'		101
**5 C**	25.96		5	6		**13 C**	127.3		13	13'	
**H2**	1.87	6	5	4, 6		**H**	7.02	12	13	11, 13'	
**6 C**	11.2		6	5		**13' C**	127.3		13'	13	
**H3**	-0.13	5	6	4, 5		**H**	7.02	12'	13'	11, 13	
**7 C**	138.51			8, 8', 9, 9'		**14 C**	129.36		14	12	
**8 C**	135.43		8	8', 10		**H**	7.11		14	12, 12'	
**H**	7.44	9	8	7, 8', 10	100	**100 Si**	-22.34				8, 8', 9, 9'
**8' C**	135.43		8'	8, 10		**101 Si**	-15.56				12, 12'
**H**	7.44		8'	7, 8, 10	100						

NMR Characterization of Complexes 8 and 9, $C_6D_5CD_3$, −40 °C

The sample was a complex mixture, but the following four different species could be identified and (at least partially) characterized. The next pages summarize the characterization data of the metallatetrahedrane **8** and the metallacyclobutadiene **9**.

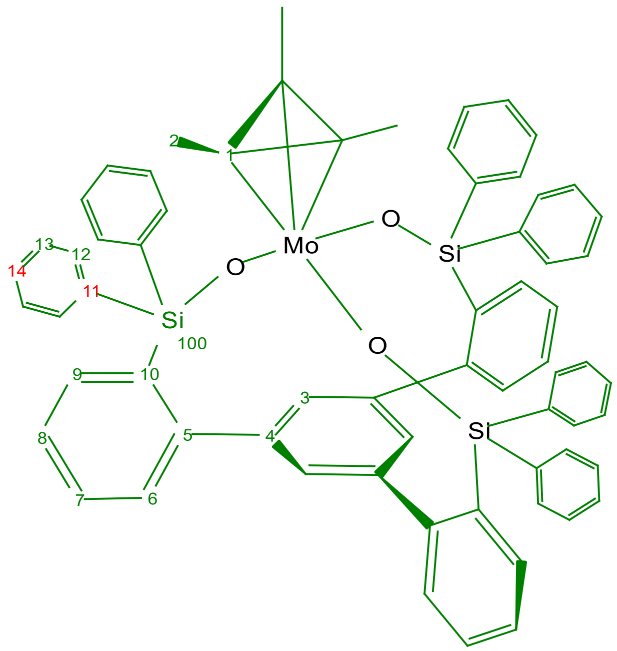

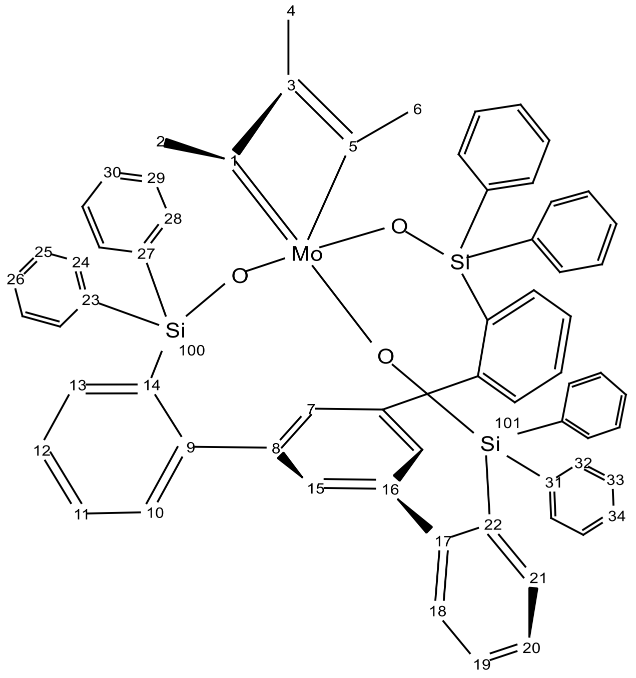

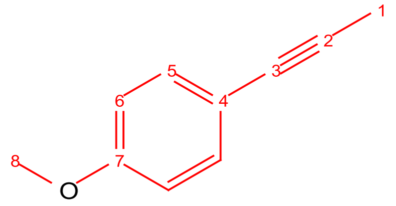

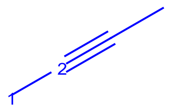

NMR Assignment: Complex 8, $C_6D_5CD_3$, −40 °C

Atom	δ (ppm)	COSY	HSQC	^{13}C-HMBC	^{29}Si-HMBC	ROESY
1 C	78.3					
2 C	8.3		2			
H3	1.29		2			12, 13
3 C	128.4		3	3		
H	7.33		3	3, 5		
4 C	144.5					
5 C	150.7			3, 7		
6 C	130.2		6			
H	6.95	7	6			
7 C	129.8		7			
H	7.15	6	7	5		
8 C	125.7		8			
H	7.16	9	8			
9 C	137.5		9			
H	7.78	8	9		100	
10 C	136.6					
11 C						
12 C	135.7		12			
H	7.84	13	12			2
13 C						
H	7.06	12				2
14 C						
H						
100 Si	-8.7				9	

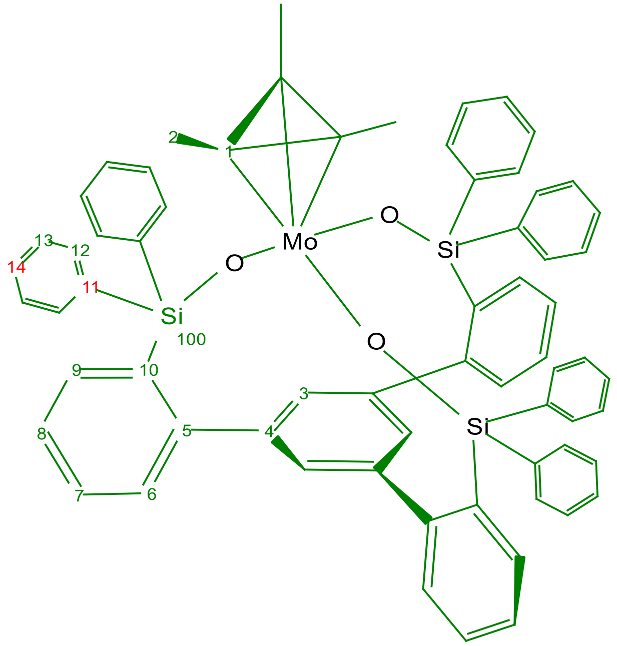

NMR Assignment: Complex 9, $C_6D_5CD_3$, –40 °C

Atom	δ (ppm)	*J*(Hz)	COSY	HSQC	^{13}C-HMBC	^{29}Si-HMBC	ROESY	Atom	δ (ppm)	COSY	HSQC	^{13}C-HMBC	^{29}Si-HMBC	ROESY
**1 C**	234.2				2, 4			**20 C**	126.4		20	18		
**2 C**	22.9			2				**H**	7.22	21	20			21
**H3**	2.41			2	1, 3		4	**21 C**	135.2		21			
**3 C**	144.6				2, 4, 6			**H**	7.85	20	21	17, 19, 22	101	20, 32
**4 C**	18.0			4				**22 C**	140.38			18, 21		
**H3**	1.71			4	1, 3, 5		2, 6, 28, 29	**23 C**	140.2			24, 25		
**5 C**	235.7				4, 6			**24 C**	135.4		24	24		
**6 C**	21.8			6				**H**	7.54	25	24	23, 24, 26	100	13, 25
**H3**	1.18			6	3, 5		4, 7, 15, 28, 29	**25 C**	127.5		25			
**7 C**	125.2			7				**H**	6.89	24, 26	25	23		24
**H**	8.26	1.20(15)	15	7	9, 15		6, 10	**26 C**	128.7		26	24		
**8 C**	143.5				10			**H**	7.04	25	26			
**9 C**	150.0				7, 11, 13, 15			**27 C**	140.5			29		
**10 C**	131.4			10	12			**28 C**	135.7		28	28		
**H**	7.35		11	10	8, 12, 14		7, 15	**H**	7.03	29	28	28, 30	100	4, 6, 13
**11 C**	129.5			11	13			**29 C**	127.6		29			
**H**	7.15		10, 12	11	9, 13			**H**	6.95	28, 30	29	27		4, 6
**12 C**	126.1			12	10			**30 C**	129.0		30	28		
**H**	7.06		11, 13	12	10, 14		13	**H**	7.05	29	30			
**13 C**	137.0			13	11			**31 C**	140.2			32, 33		
**H**	7.72		12	13	9, 11, 14	100	12, 24, 28, 32	**32 C**	135.9		32	34		
**14 C**	136.0				10, 12, 13			**H**	8.35	33	32	31, 34	101	13, 21
**15 C**	131.4			15	7, 15			**33 C**	127.8		33	33		
**H**	7.58	1.20(7)	7	15	9, 15, 17		6, 10, 18	**H**	7.08	32, 34	33	31, 33		
**16 C**	146.8				18			**34 C**	129.2		34	32		
**17 C**	148.9				15, 21			**H**	7.11	33	34	32		
**18 C**	127.6			18				**100 Si**	-20.0				13.24.28	
**H**	7.18		19	18	16, 20, 22		15	**101 Si**	-19.6				32.21	
**19 C**	129.4			19	21									
**H**	7.22		18	19										

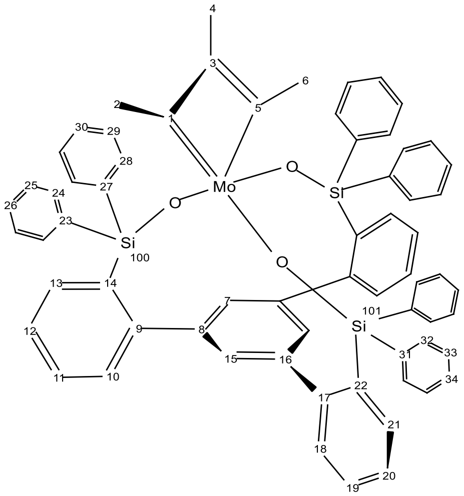

COMPUTATIONAL DATA

We applied the TPSS functional[3] with Grimme's D3(BJ) dispersion correction[4] and the reasonably large def2-TZVP basis set.[5] To speed up the calculations, we applied the RI-J approximation. Minima and transition states (TS) were confirmed by analytical computation of the Hessian and inspection of the eigenvalues and -modes. To find the TS's, we made extensive use of the Nudged-Elastic Band (NEB) method[6] to also ensure the TS's connect the correct minima. The electronic energy of all stable structures was refined on the B3LYP[7]-D3(BJ)/def2-TZVP level of theory, applying the RIJCOSX approximation. All computations were carried out using implicit solvation of toluene using the CPCM model with Gaussian surface charges.[8] The calculations were conducted with the development version of the ORCA program package.[9]

Optimized geometries

All bond lengths are in Å, all energies in E_h. The final Gibbs free energy is computed by $E_{elec} + G_{corr}$. The thermochemical Gibbs free energy differences are then the differences of these numbers, i.e.:

$$\Delta G = \sum_{products} (E_{elec} + G_{corr}) - \sum_{reactants} (E_{elec} + G_{corr})$$

2-butyne

E_elec (B3LYP)	-155.945649952448	G_corr (TPSS)	0.05683295

(the internal rotation of the methyl groups was manually treated as internal rotation in the calculation of the partition functions).

C	0.00000018	0.00000186	-0.60534079
C	0.00000065	-0.00000219	0.60534105
C	-0.00000004	0.00000563	-2.06589700
H	-0.00000221	1.02316002	-2.45880906
H	0.88607087	-0.51158065	-2.45880750
H	-0.88606911	-0.51158409	-2.45880725
C	0.00000008	-0.00000561	2.06589694
H	-0.88606906	0.51158419	2.45880675
H	-0.00000217	-1.02315984	2.45880921
H	0.88607081	0.51158070	2.45880765

Ethylidyne **10**

E_elec (B3LYP)	-3553.81138682279	G_corr (TPSS)	0.81343431

Mo	0.01279515	-0.05711145	0.00447363
Si	0.21636042	-3.36754460	-1.08345673
Si	-2.79519749	1.51612595	-1.32319247
Si	2.92892574	1.69469867	-0.76772605
O	-0.12575187	-1.85908751	-0.53288017
O	-1.42362501	1.03218374	-0.55897411
O	1.71966806	0.70255563	-0.26805216

C	-0.27644699	-1.30485661	-3.56943879
C	-1.02597176	-0.12313553	-3.63424628
H	-2.10170776	-0.18348973	-3.75352975
C	-0.40785146	1.12987318	-3.54391962
C	0.98542908	1.18981043	-3.41062577
H	1.47120711	2.15752461	-3.35813567
C	1.75626360	0.02308203	-3.34334926
C	1.11445583	-1.21840399	-3.43966501
H	1.70701021	-2.12561256	-3.40461210
C	-0.95570160	-2.62362303	-3.64955680
C	-0.75041793	-3.63464766	-2.67721982
C	-1.42689602	-4.85607917	-2.84117738
H	-1.30360626	-5.63379627	-2.09121089
C	-2.27953731	-5.08756991	-3.92053758
H	-2.78609520	-6.04370177	-4.01969923
C	-2.49337122	-4.07547385	-4.85493140
H	-3.16869470	-4.23346571	-5.69140260
C	-1.83861609	-2.85385905	-4.71361786
H	-1.99859448	-2.06512782	-5.44414599
C	-0.38325682	-4.59967634	0.19551500
C	-1.50058152	-4.29122960	0.99178938
H	-1.99133550	-3.32811042	0.87582167
C	-1.98230666	-5.19544031	1.93831171
H	-2.84492468	-4.93686025	2.54705778
C	-1.35402337	-6.43193897	2.10469087
H	-1.72787366	-7.13823560	2.84112973
C	-0.23982603	-6.75488920	1.32794969
H	0.25686646	-7.71250242	1.46068506
C	0.24118452	-5.84364241	0.38610756
H	1.11874585	-6.10195114	-0.20300200
C	2.07145342	-3.51173682	-1.29795553
C	2.93706768	-2.69080377	-0.55598358
H	2.52794428	-1.98075461	0.15864941
C	4.31816246	-2.74848588	-0.73802479
H	4.96782321	-2.08637254	-0.17244286
C	4.86173563	-3.64237654	-1.66188554
H	5.93676104	-3.68020157	-1.81453705
C	4.01997989	-4.47693137	-2.39992479
H	4.43968092	-5.17104704	-3.12338071
C	2.63763256	-4.40780176	-2.22100534
H	1.99063609	-5.04311318	-2.82312925
C	-1.20321367	2.38263242	-3.61625174
C	-2.31449750	2.62284822	-2.76966358
C	-3.01550394	3.83146965	-2.92846577
H	-3.85591227	4.04477280	-2.27241665
C	-2.64471399	4.78046211	-3.88106256
H	-3.20924918	5.70368201	-3.97895951

C	-1.53298590	4.54378246	-4.68785180
H	-1.21962711	5.28063988	-5.42238706
C	-0.81925898	3.35538087	-4.54982772
H	0.04337916	3.16447059	-5.18288590
C	-3.76269886	-0.00508860	-1.83079664
C	-3.58450907	-1.22057492	-1.14994038
H	-2.89488684	-1.27302833	-0.31104408
C	-4.25809656	-2.37527233	-1.54646648
H	-4.08560847	-3.31091880	-1.02187005
C	-5.13749756	-2.33065291	-2.62925224
H	-5.65371335	-3.23180889	-2.94831390
C	-5.34084273	-1.12881160	-3.31063680
H	-6.02203508	-1.09137656	-4.15673920
C	-4.65610055	0.02111808	-2.91574834
H	-4.80167707	0.94503391	-3.47245279
C	-3.79429396	2.53614815	-0.10838160
C	-5.19782391	2.49509255	-0.07507643
H	-5.73291055	1.83630403	-0.75571341
C	-5.92103674	3.28121019	0.82413765
H	-7.00693607	3.23542702	0.83459228
C	-5.24836602	4.12005617	1.71415807
H	-5.80892858	4.73020149	2.41733845
C	-3.85205832	4.16826779	1.70347100
H	-3.32446959	4.81469362	2.40009474
C	-3.13539775	3.38445022	0.79974604
H	-2.04868655	3.42507649	0.80418152
C	3.23256237	0.08919335	-3.19475626
C	3.85618482	0.85049290	-2.17404571
C	5.26151602	0.86305717	-2.12621366
H	5.75825765	1.42039733	-1.33572152
C	6.03834619	0.15401921	-3.04236019
H	7.12250263	0.18645385	-2.97779217
C	5.41197793	-0.61220707	-4.02388839
H	6.00189034	-1.18474169	-4.73443365
C	4.02104820	-0.64416683	-4.09260017
H	3.53017515	-1.23426419	-4.86211554
C	4.11267864	1.91478675	0.66955643
C	4.34198866	0.84435928	1.55268773
H	3.81250073	-0.09407356	1.40640196
C	5.22914229	0.96935807	2.62140302
H	5.38975034	0.13180807	3.29538701
C	5.90926437	2.17243321	2.82609128
H	6.60131135	2.27208597	3.65803329
C	5.69199356	3.24833740	1.96374771
H	6.21322188	4.18844075	2.12468601
C	4.79863630	3.11889755	0.89858480
H	4.62834325	3.96882169	0.24107283

C	2.18413983	3.34575560	-1.24577968
C	2.82430130	4.20190766	-2.15855829
H	3.77811413	3.91023681	-2.59433780
C	2.24220840	5.41190886	-2.53868666
H	2.74885297	6.05794164	-3.25091422
C	1.00179155	5.78397299	-2.01644535
H	0.53751136	6.71605760	-2.32641074
C	0.35227721	4.94840968	-1.10655178
H	-0.62004097	5.22624053	-0.70929122
C	0.94307333	3.74524959	-0.72250774
H	0.41894356	3.09961010	-0.02232911
C	-0.15256060	-0.11627920	1.73588838
C	-0.29217188	-0.17426617	3.20141114
H	-1.15033108	0.42371178	3.53700292
H	0.60818045	0.21522848	3.69569119
H	-0.44510245	-1.20857922	3.53849246

TS1

E_elec(B3LYP)	-3709.76998416374	G_corr(TPSS)	0.89370487

Mo	-0.25642206	0.94475003	-0.15111845
Si	-1.19761739	-0.34711232	2.99648786
Si	-1.35801848	-0.25896727	-3.19182230
Si	3.16758429	0.57340200	-0.13463032
O	-0.85737100	0.21820253	1.49946915
O	-1.03610790	0.66788175	-1.87534370
O	1.53156778	0.42306357	-0.32311170
C	0.82516880	-2.44281197	1.19612233
C	-0.37478041	-2.53696463	0.48116233
H	-1.31559127	-2.62072601	1.01669538
C	-0.38808400	-2.54164250	-0.92342962
C	0.82425251	-2.40890133	-1.61360669
H	0.82644384	-2.38797140	-2.69734955
C	2.02909039	-2.26431547	-0.91883878
C	2.02306939	-2.33734011	0.47716737
H	2.95887253	-2.24258779	1.01762103
C	0.87671016	-2.46268887	2.68159886
C	0.06125791	-1.65633428	3.51469120
C	0.21651040	-1.78829653	4.90775662
H	-0.38481500	-1.16788843	5.56798597
C	1.13830609	-2.66168952	5.47953424
H	1.22864958	-2.72936751	6.56020002
C	1.95180814	-3.43179969	4.64961023
H	2.68513665	-4.11290577	5.07265001

C	1.81767971	-3.32672774	3.26898329
H	2.43938037	-3.93796795	2.62025538
C	-1.66077777	-2.76428878	-1.66004299
C	-2.10649330	-1.93267049	-2.71967469
C	-3.29803704	-2.29509273	-3.37584389
H	-3.66933650	-1.66423031	-4.17910279
C	-4.04388243	-3.41357294	-3.00477490
H	-4.95920442	-3.65838540	-3.53664156
C	-3.61784839	-4.19503082	-1.93251683
H	-4.19517473	-5.05923576	-1.61531089
C	-2.43548204	-3.86872565	-1.27211439
H	-2.08616540	-4.48919042	-0.45083840
C	3.27664969	-1.89528755	-1.63708369
C	3.92576259	-0.67347784	-1.32155601
C	5.09755828	-0.34337183	-2.02204339
H	5.59681230	0.59941513	-1.80889464
C	5.61942963	-1.18223093	-3.00774046
H	6.52782497	-0.90418510	-3.53515761
C	4.95532550	-2.36639159	-3.32726265
H	5.34249657	-3.01738644	-4.10641288
C	3.78836247	-2.71573778	-2.64840765
H	3.26957113	-3.63848514	-2.89522483
C	-1.15789663	1.12866473	4.15893230
C	-0.53994158	2.31898866	3.73715934
H	-0.10559938	2.36788277	2.74159563
C	-0.49952711	3.44508059	4.56055409
H	-0.01669994	4.35465418	4.21208028
C	-1.08296088	3.40356425	5.82826335
H	-1.05317506	4.27834253	6.47243410
C	-1.71633220	2.23628795	6.26156421
H	-2.18490672	2.20329618	7.24175512
C	-1.75575542	1.11518806	5.43184536
H	-2.27478409	0.22267224	5.77552382
C	-2.91151166	-1.10668845	3.08658377
C	-3.10359113	-2.49843903	3.05101913
H	-2.24006197	-3.15875644	3.00861283
C	-4.38363577	-3.05402295	3.08341659
H	-4.50912052	-4.13311980	3.04992884
C	-5.50087335	-2.22112135	3.16600931
H	-6.49929232	-2.64960322	3.19225833
C	-5.33177141	-0.83552056	3.22486837
H	-6.19886900	-0.18406496	3.29654506
C	-4.05027481	-0.28739930	3.18616063
H	-3.93247775	0.79185046	3.22542345
C	-2.64372491	0.68577983	-4.18283517
C	-3.52509982	1.56171364	-3.52440497
H	-3.43527209	1.70489202	-2.44988706

C	-4.50732733	2.25793182	-4.23024757
H	-5.17927060	2.93046652	-3.70288434
C	-4.62531965	2.09302907	-5.61210253
H	-5.38946874	2.63492355	-6.16317613
C	-3.75239230	1.23554722	-6.28533719
H	-3.83371664	1.11169587	-7.36221088
C	-2.77048873	0.54264821	-5.57524504
H	-2.09056854	-0.11382618	-6.11458230
C	0.19357764	-0.44552016	-4.22395697
C	0.34107083	-1.50494171	-5.13634322
H	-0.44522737	-2.25250865	-5.22606686
C	1.49295809	-1.62913994	-5.91412814
H	1.59206507	-2.45786494	-6.61041962
C	2.52321847	-0.69527082	-5.78695089
H	3.42828786	-0.79826308	-6.37910578
C	2.39347633	0.36416311	-4.88743456
H	3.19970068	1.08514866	-4.78152335
C	1.23661189	0.48824198	-4.11820590
H	1.14217025	1.31089188	-3.41401339
C	3.66127601	2.32562615	-0.57855185
C	3.44038102	2.82413086	-1.87508445
H	3.06806896	2.15830991	-2.64782568
C	3.68256705	4.16050595	-2.18756856
H	3.49758513	4.52559939	-3.19445214
C	4.15849684	5.03037420	-1.20273273
H	4.34479839	6.07393854	-1.44197310
C	4.39590168	4.55450641	0.08785253
H	4.76935801	5.22700602	0.85573080
C	4.14753104	3.21501217	0.39458757
H	4.32471342	2.85756383	1.40598667
C	3.56516382	0.26904987	1.66718810
C	4.73403737	-0.39104716	2.08126781
H	5.43922265	-0.75753692	1.33771802
C	4.99494931	-0.60211886	3.43579151
H	5.89947139	-1.12257662	3.73972841
C	4.08879138	-0.15473964	4.40046666
H	4.28485811	-0.33143307	5.45447225
C	2.92455350	0.50728075	4.00784448
H	2.20762417	0.84521515	4.75053132
C	2.67042310	0.71537524	2.65326812
H	1.75435519	1.21934079	2.35624864
C	-0.11838575	2.67266924	0.04119824
C	-3.64701428	0.93241826	0.11879295
C	-3.40965941	2.07604458	0.44317943
C	0.15780797	4.11561997	0.17304367
C	-3.97666261	-0.43995608	-0.23882340
H	-4.12102683	-0.54487522	-1.31830773

H	-4.89511128	-0.75614577	0.26699335
C	-3.20064751	3.44938597	0.88571644
H	-2.81607507	4.07295970	0.07230940
H	-4.14295036	3.88673067	1.23606882
H	-0.35739102	4.69249126	-0.60666103
H	-0.16160539	4.50199600	1.15034455
H	1.23583353	4.29921279	0.07116126
H	-2.47936452	3.48410812	1.70899672
H	-3.17814885	-1.12424728	0.06192588

TS1’

E_elec(B3LYP)	-3709.73315066665	G_corr(TPSS)	0.89665388

Mo	-0.158976	-0.120946	1.274658
Si	-2.588687	1.907669	-0.110138
Si	2.942695	1.208686	-0.193174
Si	-0.310126	-3.186612	-0.130829
O	-1.212597	1.107784	0.325149
O	1.583706	0.699196	0.504988
O	0.191139	-1.652106	0.228302
C	-1.450183	0.029460	-2.597249
C	-0.608701	1.145884	-2.627371
H	-1.035652	2.143400	-2.590158
C	0.782102	0.997962	-2.667421
C	1.329432	-0.289058	-2.707194
H	2.407218	-0.412853	-2.723579
C	0.506965	-1.421652	-2.686532
C	-0.881417	-1.249488	-2.656130
H	-1.529318	-2.119156	-2.647441
C	-2.921284	0.187163	-2.477420
C	-3.513719	0.987804	-1.467064
C	-4.917151	1.088340	-1.441790
H	-5.391543	1.683005	-0.664603
C	-5.722652	0.423661	-2.366135
H	-6.803669	0.522579	-2.317546
C	-5.129020	-0.381220	-3.337183
H	-5.741882	-0.916459	-4.057411
C	-3.741878	-0.498570	-3.384602
H	-3.276496	-1.119379	-4.145555
C	1.654104	2.200828	-2.647384
C	2.658595	2.376043	-1.663943
C	3.444221	3.541322	-1.737180
H	4.214166	3.705416	-0.987374
C	3.250060	4.508762	-2.724236

H	3.878365	5.395393	-2.749147
C	2.233714	4.337039	-3.663217
H	2.056104	5.089047	-4.427391
C	1.442140	3.191140	-3.617506
H	0.653755	3.046326	-4.352135
C	1.103009	-2.781724	-2.629744
C	0.758289	-3.692182	-1.596679
C	1.361928	-4.961248	-1.601235
H	1.125423	-5.663696	-0.805066
C	2.281881	-5.333982	-2.582419
H	2.733057	-6.322323	-2.560345
C	2.632684	-4.420832	-3.576393
H	3.361168	-4.690340	-4.336309
C	2.047830	-3.155573	-3.594409
H	2.316802	-2.443642	-4.370501
C	-3.716473	2.081342	1.382114
C	-4.341835	0.954630	1.946377
H	-4.232703	-0.015022	1.468926
C	-5.101491	1.055606	3.111394
H	-5.574350	0.170721	3.529790
C	-5.255888	2.295015	3.739088
H	-5.848880	2.376532	4.646122
C	-4.650352	3.427924	3.192669
H	-4.771814	4.395307	3.673346
C	-3.888050	3.318382	2.026821
H	-3.415168	4.206452	1.613946
C	-1.984886	3.601432	-0.636494
C	-2.819838	4.516165	-1.300877
H	-3.850772	4.247314	-1.523825
C	-2.343116	5.768087	-1.692805
H	-3.000730	6.462354	-2.209601
C	-1.018580	6.125298	-1.427687
H	-0.643829	7.095523	-1.742800
C	-0.174506	5.227769	-0.771659
H	0.862192	5.492712	-0.582957
C	-0.654816	3.978609	-0.380414
H	0.015267	3.271561	0.100931
C	3.897637	2.222562	1.087625
C	3.210493	3.232686	1.786142
H	2.166127	3.427378	1.550952
C	3.835582	3.982962	2.780602
H	3.283634	4.757861	3.307190
C	5.174409	3.736660	3.102407
H	5.665064	4.318125	3.878590
C	5.875708	2.739050	2.423580
H	6.915930	2.541119	2.670793
C	5.239742	1.992313	1.427834

H	5.795222	1.211876	0.912285
C	4.027652	-0.240307	-0.712515
C	5.164247	-0.066913	-1.521814
H	5.431148	0.929686	-1.869481
C	5.952427	-1.153795	-1.904353
H	6.824919	-0.999167	-2.534386
C	5.612516	-2.442382	-1.485489
H	6.217576	-3.291952	-1.791284
C	4.485169	-2.637370	-0.685525
H	4.205012	-3.641215	-0.377568
C	3.704732	-1.545053	-0.304608
H	2.805337	-1.700431	0.285085
C	0.096896	-4.237406	1.368274
C	1.410836	-4.253517	1.872318
H	2.193613	-3.707897	1.351141
C	1.731187	-4.957076	3.032190
H	2.751993	-4.955206	3.405835
C	0.737465	-5.665143	3.713124
H	0.983754	-6.214714	4.617876
C	-0.571442	-5.664394	3.229149
H	-1.346677	-6.213515	3.757373
C	-0.885726	-4.956517	2.066664
H	-1.910312	-4.954936	1.701795
C	-2.140948	-3.320500	-0.507370
C	-2.601303	-4.372589	-1.319667
H	-1.890650	-5.084352	-1.735939
C	-3.957419	-4.510057	-1.619268
H	-4.292692	-5.328104	-2.251762
C	-4.880687	-3.591018	-1.115368
H	-5.935770	-3.690775	-1.355621
C	-4.440784	-2.537774	-0.312506
H	-5.151560	-1.807214	0.064015
C	-3.085974	-2.407441	-0.011591
H	-2.743440	-1.580008	0.605605
C	-1.322016	-0.824606	2.428370
C	0.268478	1.098683	3.436674
C	1.300337	0.467827	3.223520
C	-2.023312	-1.382837	3.597361
C	-0.814119	1.989745	3.854717
H	-0.465106	2.587836	4.705522
H	-1.700080	1.424298	4.153274
C	2.579169	-0.243273	3.229606
H	3.295320	0.248567	2.568403
H	2.971916	-0.236275	4.253305
H	-2.244117	-2.447255	3.436239
H	-1.400547	-1.308922	4.501342
H	-2.972334	-0.865665	3.792573

H	2.461351	-1.284577	2.913685
H	-1.100562	2.668340	3.046716

MCBD 9

E_elec(B3LYP)	-3709.78460892226	G_corr(TPSS)	0.89843476

Mo	-0.57662097	0.81438451	-0.00923030
Si	-0.90054935	-0.25991458	3.31782037
Si	2.95595862	0.53012027	-0.07478960
Si	-0.99517651	-0.38330344	-3.22753332
O	-0.42708228	0.47584645	1.94405472
O	1.31145569	0.48307768	-0.02213235
O	-0.43250846	0.50098679	-1.97269243
C	0.75160837	-2.51598080	1.36070766
C	-0.45780940	-2.56546671	0.65398822
H	-1.39697085	-2.62061913	1.19678577
C	-0.47142579	-2.56948397	-0.75375051
C	0.74239055	-2.48052933	-1.44267681
H	0.74526776	-2.47662724	-2.52729069
C	1.94824087	-2.34707515	-0.75626450
C	1.94877093	-2.41250656	0.63953811
H	2.88722065	-2.33596696	1.17880169
C	0.83416424	-2.61802016	2.84353404
C	0.16194084	-1.76125457	3.74884005
C	0.35473635	-1.98748823	5.12540125
H	-0.13178820	-1.32964914	5.84089910
C	1.17772633	-3.00093082	5.61105564
H	1.30160073	-3.13698147	6.68197159
C	1.85306965	-3.82145280	4.70915330
H	2.51000018	-4.61083212	5.06417041
C	1.67923416	-3.62447465	3.34268815
H	2.19537535	-4.26811532	2.63513387
C	-0.75423721	1.03603739	4.67782742
C	0.05727584	2.16757345	4.48875100
H	0.58783458	2.28989212	3.54841858
C	0.18514674	3.14187184	5.48016759
H	0.82112564	4.00724301	5.31152354
C	-0.50566006	3.00590316	6.68568796
H	-0.40773325	3.76252053	7.45971806
C	-1.33148173	1.89804137	6.88909940
H	-1.88148637	1.79284314	7.82082390
C	-1.45502648	0.92925176	5.89253716
H	-2.11645882	0.08119960	6.05939101
C	-2.70829266	-0.77787777	3.27887839

C	-3.11344622	-2.12222780	3.28377229
H	-2.36164579	-2.90701412	3.33623677
C	-4.46449438	-2.47188684	3.23063832
H	-4.75625751	-3.51894464	3.23289151
C	-5.43993114	-1.47466348	3.17974101
H	-6.49216330	-1.74315798	3.13871291
C	-5.05999554	-0.13005833	3.19113045
H	-5.81766293	0.64899648	3.15945673
C	-3.70916543	0.21009520	3.24058819
H	-3.42454752	1.26034885	3.25330822
C	3.18170055	-1.98504093	-1.49852766
C	3.72016222	-0.69511508	-1.28938423
C	4.86420675	-0.32997521	-2.01768408
H	5.28216310	0.66636442	-1.89155647
C	5.46440343	-1.21139183	-2.91788559
H	6.35061163	-0.90700137	-3.46838110
C	4.90832383	-2.47324139	-3.12666018
H	5.35794247	-3.15769377	-3.84086250
C	3.76416215	-2.85378606	-2.42592688
H	3.32090305	-3.83227751	-2.59218423
C	3.39260190	2.26687755	-0.64493574
C	2.54219662	2.93314849	-1.54561449
H	1.63155810	2.44299441	-1.88421021
C	2.84024602	4.21685843	-2.00267250
H	2.16891407	4.71512287	-2.69768370
C	4.00055779	4.86143331	-1.56633129
H	4.23451804	5.86204198	-1.92023231
C	4.85551121	4.21854363	-0.66933929
H	5.75526463	4.71939488	-0.32113607
C	4.55022191	2.93450270	-0.21281757
H	5.21736920	2.45011550	0.49734082
C	3.65584468	0.20358842	1.63202021
C	4.97289198	-0.25066166	1.81938918
H	5.61370286	-0.42336059	0.95688187
C	5.46708482	-0.50874845	3.09890028
H	6.48665795	-0.86398163	3.22576923
C	4.64741679	-0.32337075	4.21515388
H	5.02768264	-0.53661935	5.21072959
C	3.33591585	0.12426008	4.04718073
H	2.68692261	0.25467189	4.90866581
C	2.84981108	0.38624659	2.76698862
H	1.82009994	0.70278135	2.63149422
C	-1.72964406	-2.69886199	-1.52743610
C	-2.00044009	-1.89707599	-2.66886300
C	-3.19015243	-2.15649814	-3.37457412
H	-3.42781710	-1.55192888	-4.24602787
C	-4.09732130	-3.13841398	-2.97440829

H	-5.00588552	-3.30590891	-3.54647040
C	-3.83539394	-3.88797354	-1.82846515
H	-4.53706676	-4.64731020	-1.49407302
C	-2.65759684	-3.66826312	-1.11863174
H	-2.43592708	-4.26859255	-0.23998535
C	0.43112051	-0.84321752	-4.35400790
C	0.32710561	-1.88999585	-5.28594490
H	-0.58934397	-2.47496703	-5.33999549
C	1.38685453	-2.20269506	-6.13846578
H	1.29025867	-3.01914274	-6.84967251
C	2.57335196	-1.46892334	-6.07282082
H	3.40283262	-1.71533619	-6.73041483
C	2.69591501	-0.42683995	-5.15234858
H	3.62279108	0.13518447	-5.08567153
C	1.63432106	-0.11984566	-4.30192907
H	1.74321408	0.68022705	-3.57547907
C	-2.17050611	0.72382643	-4.20213197
C	-1.90193615	1.12521169	-5.52031080
H	-1.01611579	0.74580070	-6.02436953
C	-2.75027142	2.00573589	-6.19707787
H	-2.52287778	2.30145983	-7.21811498
C	-3.88708881	2.50699615	-5.56225891
H	-4.54740700	3.19305313	-6.08614957
C	-4.17400986	2.12175247	-4.24946321
H	-5.05989187	2.50694811	-3.75022883
C	-3.32423223	1.24058487	-3.58350854
H	-3.56255604	0.94288467	-2.56554385
C	-3.68452947	-0.51317559	-0.02931228
H	-4.59459382	0.00658821	0.28678569
H	-3.53309957	-1.36576197	0.63691974
C	-2.49664334	0.37153429	-0.00657948
C	-2.47562043	1.78384671	0.00993433
C	-1.19534871	2.56700690	0.00596313
C	-3.73250108	2.61830891	0.03629586
H	-3.71435218	3.27990456	0.90810563
H	-4.62091794	1.98788116	0.07468689
C	-1.09611144	4.04809501	0.02855856
H	-1.60991749	4.49350397	-0.83347074
H	-1.54262720	4.46281953	0.94205257
H	-0.04477987	4.34360905	-0.00636306
H	-3.84322800	-0.91313372	-1.03738166
H	-3.76989824	3.24567774	-0.86021378

TS2

E_elec(B3LYP)	-3709.76001584385	G_corr(TPSS)	0.89620252

Mo	-0.285318	-0.034172	1.127619
Si	-0.281547	-3.294405	-0.141316
Si	-2.325155	2.316652	-0.105913
Si	2.866611	1.043810	-0.349964
O	-0.223513	-1.685035	0.211982
O	-0.910696	1.564003	0.293419
O	1.464826	0.491662	0.260979
C	0.404264	-1.366007	-2.728156
C	-0.966939	-1.154399	-2.554570
H	-1.631147	-2.003020	-2.428019
C	-1.497579	0.140285	-2.485309
C	-0.635283	1.232373	-2.631272
H	-1.031056	2.242658	-2.586307
C	0.740332	1.043607	-2.775689
C	1.247660	-0.256794	-2.847331
H	2.316818	-0.408548	-2.955062
C	0.998395	-2.725464	-2.720818
C	0.754299	-3.643736	-1.672089
C	1.377043	-4.903539	-1.735762
H	1.220288	-5.616177	-0.929013
C	2.218538	-5.257458	-2.788642
H	2.686777	-6.237766	-2.806551
C	2.470135	-4.335302	-3.804691
H	3.135169	-4.590024	-4.625383
C	1.866723	-3.081389	-3.764219
H	2.056364	-2.362113	-4.556677
C	-2.941483	0.356242	-2.229828
C	-3.402700	1.320087	-1.294715
C	-4.793351	1.516518	-1.189782
H	-5.172860	2.255785	-0.488797
C	-5.707669	0.774762	-1.936878
H	-6.773575	0.955205	-1.828205
C	-5.241504	-0.210432	-2.806897
H	-5.939924	-0.810924	-3.383412
C	-3.871077	-0.410230	-2.950255
H	-3.504138	-1.153216	-3.653396
C	1.670280	2.196535	-2.770473
C	2.686583	2.273339	-1.787981
C	3.573140	3.363865	-1.857315
H	4.363170	3.453380	-1.116164
C	3.455664	4.350915	-2.837229
H	4.159619	5.178699	-2.861729
C	2.420712	4.279336	-3.769218

H	2.305980	5.052545	-4.524156
C	1.532868	3.206087	-3.730919
H	0.730123	3.135439	-4.460671
C	0.423246	-4.172850	1.360992
C	1.255004	-3.490878	2.265388
H	1.481720	-2.439261	2.104114
C	1.793819	-4.134291	3.379885
H	2.433292	-3.585125	4.066030
C	1.509969	-5.481596	3.612061
H	1.928411	-5.985660	4.479102
C	0.680668	-6.177577	2.729871
H	0.449634	-7.224095	2.910714
C	0.141937	-5.526357	1.619399
H	-0.516015	-6.076540	0.949923
C	-2.023912	-3.934444	-0.428910
C	-2.472529	-4.291594	-1.712492
H	-1.792324	-4.217647	-2.558182
C	-3.773456	-4.750479	-1.923668
H	-4.099117	-5.016706	-2.925758
C	-4.653476	-4.869600	-0.846755
H	-5.668197	-5.223436	-1.008206
C	-4.220831	-4.546082	0.441462
H	-4.896821	-4.652746	1.285951
C	-2.918610	-4.090567	0.644086
H	-2.588628	-3.862148	1.653183
C	-3.303226	2.634591	1.470350
C	-4.229858	1.716485	1.993842
H	-4.453318	0.806999	1.443551
C	-4.876374	1.951814	3.207654
H	-5.592499	1.228881	3.590117
C	-4.599484	3.112979	3.933000
H	-5.101574	3.298149	4.878871
C	-3.674428	4.034400	3.437782
H	-3.454264	4.939565	3.997700
C	-3.037526	3.795526	2.218986
H	-2.325785	4.525159	1.839068
C	-1.779222	3.965070	-0.814410
C	-2.682932	4.845107	-1.434232
H	-3.732053	4.569857	-1.525387
C	-2.254705	6.070516	-1.945748
H	-2.966237	6.737610	-2.425610
C	-0.910001	6.437905	-1.845207
H	-0.575157	7.390055	-2.248353
C	0.002164	5.576311	-1.233726
H	1.051614	5.848952	-1.166332
C	-0.431111	4.351906	-0.724497
H	0.283947	3.672600	-0.270217

C	3.776608	1.983064	1.011097
C	3.338677	3.254829	1.424369
H	2.507991	3.731079	0.907550
C	3.947600	3.922635	2.486422
H	3.591304	4.904577	2.787858
C	5.013548	3.326371	3.165605
H	5.490135	3.843712	3.994168
C	5.467306	2.066852	2.770907
H	6.299307	1.600271	3.292462
C	4.853519	1.406548	1.703605
H	5.216951	0.425949	1.404030
C	3.918235	-0.428853	-0.858726
C	5.043064	-0.303676	-1.691808
H	5.323407	0.675616	-2.075801
C	5.802880	-1.418832	-2.048359
H	6.666383	-1.303418	-2.698822
C	5.449570	-2.685293	-1.574472
H	6.035648	-3.555684	-1.857841
C	4.335669	-2.830337	-0.746071
H	4.044934	-3.813877	-0.387904
C	3.579474	-1.711469	-0.397454
H	2.698442	-1.829829	0.226329
C	0.460128	0.394618	2.945477
C	-1.690056	-0.555243	2.326601
C	-0.850926	0.206390	3.312486
C	1.577751	1.106251	3.609821
C	-2.877998	-1.306248	2.812741
H	-3.492617	-0.701830	3.491526
H	-2.562974	-2.199717	3.372749
C	-1.501463	0.791135	4.546033
H	-2.349767	1.416771	4.249700
H	-1.887069	-0.013985	5.180908
H	1.671312	2.134659	3.243096
H	1.446399	1.131782	4.698421
H	2.527638	0.613968	3.381014
H	-0.788278	1.388633	5.117314
H	-3.500355	-1.631813	1.976347

Intermediate 12

E_elec(B3LYP)	-3709.77191509919	G_corr(TPSS)	0.89523293

Mo	0.00670486	-0.05310981	1.17808684
Si	-2.41009157	2.05670217	-0.02735542
Si	3.02315944	1.20996454	-0.36807372

Si	-0.25853568	-3.27736063	-0.08189696
O	-0.97835029	1.35623617	0.36815466
O	1.61679074	0.70540834	0.28315840
O	0.14942172	-1.74815936	0.41895630
C	-1.41820088	0.05995931	-2.50075579
C	-0.57113365	1.16687678	-2.60774602
H	-0.98861902	2.16895015	-2.57547628
C	0.81125827	1.00234837	-2.72557632
C	1.34458073	-0.29041454	-2.75424305
H	2.41816102	-0.42466299	-2.83502736
C	0.51666656	-1.41371950	-2.64985826
C	-0.86650389	-1.22637915	-2.55159873
H	-1.52207560	-2.08797493	-2.49152460
C	-2.87788197	0.24705572	-2.32295810
C	-3.40244698	1.10140798	-1.32108500
C	-4.80157974	1.24444971	-1.25117603
H	-5.23119829	1.88289938	-0.48292227
C	-5.66038183	0.57291399	-2.12061159
H	-6.73509442	0.71051986	-2.03819422
C	-5.12940077	-0.28920009	-3.07901601
H	-5.78508106	-0.83393100	-3.75282852
C	-3.74977152	-0.44985807	-3.17149600
H	-3.33031039	-1.11330796	-3.92324934
C	1.70779768	2.18110072	-2.79094268
C	2.77201549	2.33593313	-1.87026499
C	3.62099937	3.44524961	-2.03804065
H	4.44780902	3.58891496	-1.34736403
C	3.41963943	4.38467177	-3.04996832
H	4.09580535	5.22954878	-3.15034226
C	2.33612728	4.24258181	-3.91623023
H	2.15507074	4.97733536	-4.69611946
C	1.48841892	3.14482583	-3.78422544
H	0.65350453	3.01589250	-4.46838903
C	1.11175952	-2.77280869	-2.61184362
C	0.80610806	-3.68962465	-1.57451619
C	1.41348767	-4.95719563	-1.60677929
H	1.20743840	-5.66406005	-0.80643889
C	2.29929854	-5.32195739	-2.62047412
H	2.75294741	-6.30922214	-2.61862735
C	2.61328684	-4.40227459	-3.61998325
H	3.31503724	-4.66516284	-4.40681336
C	2.02583222	-3.13893893	-3.60908472
H	2.26528788	-2.42268605	-4.39063412
C	-3.48557015	2.17526576	1.50734277
C	-4.12640108	1.02985718	2.01540639
H	-4.04384099	0.08767435	1.48155281
C	-4.87278398	1.07989360	3.19169572

H	-5.35733124	0.18196354	3.56644040
C	-4.99364784	2.28448888	3.88919736
H	-5.57580319	2.32746111	4.80579367
C	-4.36104254	3.43173987	3.40705399
H	-4.45072198	4.37024341	3.94781206
C	-3.61297857	3.37359449	2.22872984
H	-3.12416727	4.27331772	1.86195800
C	-1.92949622	3.76633443	-0.62936345
C	-2.87057862	4.66949547	-1.15256721
H	-3.92076287	4.38846236	-1.20618661
C	-2.47938141	5.92595050	-1.61628560
H	-3.22041491	6.61120062	-2.01974784
C	-1.13383069	6.29984409	-1.56921988
H	-0.82750111	7.27516880	-1.93808055
C	-0.18420377	5.41525650	-1.05507312
H	0.86575483	5.69461034	-1.03005702
C	-0.58115760	4.16214988	-0.58796940
H	0.16164622	3.46748845	-0.20638315
C	3.97197964	2.22068737	0.90667534
C	3.58961540	3.53656931	1.22533232
H	2.77828530	4.00866516	0.67503216
C	4.22651997	4.25145294	2.23859488
H	3.91193173	5.26622610	2.46886229
C	5.26631281	3.66069027	2.96103027
H	5.76408546	4.21505249	3.75227677
C	5.66599522	2.35785097	2.65948532
H	6.47722644	1.89494161	3.21563333
C	5.02402937	1.64949697	1.64127930
H	5.34414393	0.63487007	1.41451873
C	4.03742946	-0.30955531	-0.80935059
C	5.11324125	-0.24986580	-1.71133209
H	5.37905135	0.69995049	-2.17174885
C	5.83987519	-1.39443391	-2.04226387
H	6.66518565	-1.32967260	-2.74686599
C	5.50037584	-2.62533698	-1.47521775
H	6.05719408	-3.51988813	-1.74130584
C	4.43613818	-2.70478586	-0.57604334
H	4.15681984	-3.66275519	-0.14585565
C	3.71462530	-1.55654631	-0.24960650
H	2.87066630	-1.62661372	0.43049138
C	0.17481117	-4.43354595	1.32787375
C	1.40921516	-4.29336620	1.98777446
H	2.11012151	-3.52804374	1.66361011
C	1.74935687	-5.11380084	3.06212668
H	2.70621745	-4.98608784	3.56142678
C	0.85790108	-6.09782978	3.49740855
H	1.12039320	-6.73815475	4.33524224

C	-0.37200899	-6.25249860	2.85648276
H	-1.07065444	-7.01297667	3.19532241
C	-0.70908162	-5.42440920	1.78399965
H	-1.67687167	-5.54408685	1.30289813
C	-2.09421759	-3.37627384	-0.43142544
C	-2.62037515	-4.48691423	-1.11515545
H	-1.95563182	-5.27864379	-1.45590213
C	-3.98693666	-4.58509819	-1.38001505
H	-4.37585995	-5.44991368	-1.91106173
C	-4.85121167	-3.56729739	-0.97103361
H	-5.91397530	-3.63592179	-1.18685376
C	-4.34446326	-2.45334476	-0.30053898
H	-5.00906342	-1.64648131	-0.00617289
C	-2.97965937	-2.36212944	-0.03393154
H	-2.59028905	-1.48112411	0.47269885
C	-1.10492333	-0.74047312	2.63219658
C	-0.59550256	0.36468628	3.45005957
C	0.65589322	0.69033190	2.99062089
C	-1.79588791	-1.91428044	3.23502621
C	-1.39947680	1.08965880	4.49771470
H	-1.81875965	0.37249600	5.21197050
H	-2.23926697	1.61070604	4.02727703
C	1.58701663	1.76646761	3.42032804
H	1.50805331	2.64104784	2.76395851
H	1.38250437	2.08506398	4.45028131
H	-1.14892048	-2.41606206	3.97019148
H	-2.69963864	-1.59075894	3.77236823
H	-2.09177211	-2.64856437	2.48262119
H	2.62657609	1.43111704	3.35479237
H	-0.78788273	1.81830711	5.03554214

TS_{BR}

E_elec(B3LYP)	-3709.76876782589	G_corr(TPSS)	0.90117066

Mo	-0.042431	0.179417	1.157609
Si	-0.362859	-3.203602	-0.008100
Si	-2.614979	2.018067	-0.239975
Si	3.012054	1.051438	-0.292252
O	0.026988	-1.710148	0.513241
O	-1.423035	0.955205	0.224271
O	1.630697	0.378731	0.267036
C	0.379004	-1.405720	-2.638232
C	-1.005400	-1.199795	-2.621487
H	-1.672765	-2.053589	-2.571951

C	-1.541645	0.094439	-2.639819
C	-0.667466	1.186759	-2.701725
H	-1.068286	2.194616	-2.727437
C	0.720937	1.001765	-2.688858
C	1.230881	-0.299491	-2.680875
H	2.305401	-0.456182	-2.686684
C	0.948440	-2.774958	-2.599340
C	0.634647	-3.674345	-1.552522
C	1.177658	-4.971080	-1.625814
H	0.954182	-5.684114	-0.836827
C	2.018620	-5.365599	-2.667342
H	2.417924	-6.376190	-2.691627
C	2.361013	-4.448650	-3.660238
H	3.035961	-4.732222	-4.463494
C	1.825592	-3.162832	-3.621991
H	2.074874	-2.446981	-4.401267
C	-3.012143	0.289832	-2.545457
C	-3.593001	1.152100	-1.579483
C	-4.992890	1.272029	-1.539971
H	-5.449194	1.906568	-0.783191
C	-5.813498	0.569500	-2.421379
H	-6.893033	0.679833	-2.367576
C	-5.235339	-0.291466	-3.353544
H	-5.861269	-0.857560	-4.037662
C	-3.849723	-0.430945	-3.407814
H	-3.401267	-1.101636	-4.135854
C	1.646175	2.160217	-2.667683
C	2.693907	2.256096	-1.716240
C	3.551654	3.369244	-1.799392
H	4.355590	3.473041	-1.074680
C	3.388296	4.361248	-2.766503
H	4.071029	5.205940	-2.800432
C	2.332530	4.269287	-3.672265
H	2.180504	5.041969	-4.421009
C	1.470704	3.176934	-3.616639
H	0.653959	3.093254	-4.328820
C	0.077201	-4.505713	1.281135
C	1.409446	-4.640006	1.714020
H	2.186200	-4.030098	1.257284
C	1.756847	-5.530922	2.728029
H	2.791098	-5.608653	3.053557
C	0.771686	-6.316088	3.333450
H	1.037198	-7.005263	4.130708
C	-0.553128	-6.214120	2.907013
H	-1.322336	-6.826427	3.370502
C	-0.892359	-5.317834	1.889754
H	-1.930241	-5.239604	1.574117

C	-2.208533	-3.312592	-0.358357
C	-2.749935	-4.336448	-1.156622
H	-2.094263	-5.095924	-1.578801
C	-4.116735	-4.388163	-1.435523
H	-4.515267	-5.184584	-2.059237
C	-4.971525	-3.409327	-0.922765
H	-6.033723	-3.438519	-1.151126
C	-4.454455	-2.386757	-0.126114
H	-5.113060	-1.613457	0.259628
C	-3.088024	-2.344785	0.152091
H	-2.687702	-1.535204	0.757108
C	-3.681676	2.318559	1.272331
C	-4.177757	1.226709	2.008788
H	-3.964357	0.212587	1.681341
C	-4.934109	1.419100	3.163780
H	-5.304815	0.561731	3.719423
C	-5.213780	2.714692	3.606527
H	-5.804945	2.867204	4.505725
C	-4.728944	3.811375	2.892612
H	-4.939715	4.820528	3.236887
C	-3.967237	3.612524	1.738715
H	-3.582842	4.475486	1.200675
C	-1.812394	3.620736	-0.771800
C	-2.564824	4.599979	-1.444339
H	-3.612689	4.416270	-1.673698
C	-1.983539	5.805811	-1.838592
H	-2.578736	6.548769	-2.362568
C	-0.636018	6.052877	-1.566583
H	-0.179816	6.987748	-1.880593
C	0.128048	5.089055	-0.907024
H	1.182989	5.263239	-0.716649
C	-0.457549	3.885750	-0.517623
H	0.157480	3.130806	-0.035481
C	3.811338	2.027587	1.101764
C	3.252774	3.245878	1.530244
H	2.395936	3.659037	1.003513
C	3.780198	3.943006	2.616088
H	3.333698	4.884176	2.926881
C	4.885563	3.432047	3.301920
H	5.302586	3.975634	4.145674
C	5.454365	2.223719	2.896295
H	6.314599	1.822637	3.426127
C	4.918940	1.530048	1.808242
H	5.370250	0.589034	1.501956
C	4.131234	-0.369064	-0.790825
C	5.347490	-0.165733	-1.466016
H	5.668508	0.844163	-1.714912

C	6.153549	-1.243837	-1.834770
H	7.088727	-1.068704	-2.360855
C	5.754765	-2.548938	-1.534186
H	6.378093	-3.389509	-1.828172
C	4.549810	-2.770170	-0.864868
H	4.226807	-3.783817	-0.643685
C	3.747684	-1.689211	-0.498104
H	2.797396	-1.863809	-0.000850
C	-0.027040	1.446324	2.665636
C	-0.216850	-0.755485	3.034342
C	0.068930	0.422130	3.684402
C	-0.272250	2.890197	2.965556
C	-0.169997	-2.138224	3.573036
H	-0.298464	-2.136847	4.663811
H	0.792197	-2.615735	3.348338
C	0.453311	0.636961	5.122793
H	-0.005950	-0.117250	5.768456
H	1.541668	0.560888	5.238653
H	-0.519336	3.474139	2.077097
H	-1.091772	3.003370	3.690181
H	0.625034	3.338053	3.417117
H	0.152323	1.633212	5.461122
H	-0.936281	-2.777378	3.127589

TS3

E_elec(B3LYP)	-3709.75870389877	G_corr(TPSS)	0.89442961

Mo	-0.086057	0.034640	1.149512
Si	-0.214598	-3.234571	-0.045366
Si	-2.528366	2.194039	-0.017423
Si	2.937528	1.163213	-0.420575
O	0.172451	-1.711584	0.434493
O	-1.191333	1.470793	0.632979
O	1.495829	0.656105	0.129185
C	0.477377	-1.375016	-2.648568
C	-0.897604	-1.195598	-2.469719
H	-1.540988	-2.061960	-2.361582
C	-1.459629	0.085483	-2.408121
C	-0.625467	1.199538	-2.558188
H	-1.046885	2.199129	-2.519352
C	0.751318	1.042544	-2.743483
C	1.289786	-0.246840	-2.809012
H	2.359228	-0.372949	-2.944548
C	1.071647	-2.733405	-2.631737

C	0.788486	-3.651688	-1.589163
C	1.385237	-4.923385	-1.651390
H	1.201265	-5.634166	-0.849083
C	2.236096	-5.289668	-2.694644
H	2.680727	-6.281016	-2.712617
C	2.528951	-4.366380	-3.697449
H	3.203972	-4.629909	-4.507197
C	1.952044	-3.098859	-3.659505
H	2.171597	-2.379598	-4.444330
C	-2.920141	0.248466	-2.221185
C	-3.471398	1.152334	-1.278835
C	-4.874837	1.272667	-1.234989
H	-5.325722	1.956237	-0.520881
C	-5.712487	0.524590	-2.061148
H	-6.790129	0.647957	-1.997385
C	-5.155707	-0.392055	-2.951250
H	-5.792806	-0.998188	-3.589439
C	-3.772346	-0.523461	-3.025490
H	-3.333035	-1.221982	-3.732429
C	1.643092	2.227559	-2.797093
C	2.699701	2.361248	-1.864176
C	3.525512	3.494309	-1.964453
H	4.332008	3.627474	-1.247007
C	3.320822	4.468927	-2.942295
H	3.977486	5.333296	-2.996200
C	2.256262	4.337319	-3.834000
H	2.074521	5.098927	-4.587583
C	1.421333	3.223760	-3.756152
H	0.592891	3.114268	-4.451597
C	0.330628	-4.400563	1.323967
C	1.499164	-4.115556	2.053147
H	2.074190	-3.223308	1.818330
C	1.926006	-4.948303	3.086762
H	2.830085	-4.707565	3.640368
C	1.189631	-6.090468	3.411999
H	1.519247	-6.740277	4.218313
C	0.026613	-6.391017	2.701164
H	-0.552518	-7.275452	2.953998
C	-0.397206	-5.550840	1.669196
H	-1.313839	-5.787791	1.133578
C	-2.058556	-3.419277	-0.331022
C	-2.565992	-4.464501	-1.123281
H	-1.882061	-5.174826	-1.584341
C	-3.937376	-4.595390	-1.348705
H	-4.310936	-5.407974	-1.966554
C	-4.827354	-3.674750	-0.790906
H	-5.894073	-3.767057	-0.977102

C	-4.340917	-2.627356	-0.006712
H	-5.027980	-1.896570	0.411915
C	-2.970992	-2.504079	0.222480
H	-2.595705	-1.674508	0.819138
C	-3.712649	2.498420	1.406753
C	-4.391770	1.415312	1.997407
H	-4.284686	0.419315	1.573612
C	-5.193895	1.594334	3.122866
H	-5.706780	0.744722	3.566085
C	-5.334202	2.867277	3.682954
H	-5.958625	3.009876	4.560850
C	-4.671079	3.954373	3.111898
H	-4.778963	4.945620	3.544402
C	-3.867293	3.768301	1.984341
H	-3.350200	4.620768	1.549831
C	-1.902246	3.813134	-0.717670
C	-2.725287	4.654786	-1.485304
H	-3.758327	4.372570	-1.679753
C	-2.233534	5.848152	-2.015877
H	-2.882346	6.486500	-2.609912
C	-0.904797	6.217208	-1.791170
H	-0.518478	7.141188	-2.213095
C	-0.072079	5.392059	-1.033350
H	0.966938	5.665083	-0.871375
C	-0.569414	4.202911	-0.501434
H	0.086262	3.551461	0.068465
C	3.813022	2.121344	0.947379
C	3.220864	3.282960	1.478262
H	2.298522	3.662125	1.043705
C	3.788578	3.958876	2.557176
H	3.311411	4.852362	2.952034
C	4.972361	3.486072	3.131177
H	5.417451	4.010303	3.972762
C	5.580548	2.339708	2.618241
H	6.501873	1.968625	3.060079
C	5.003282	1.666538	1.538119
H	5.481678	0.769275	1.152153
C	3.988543	-0.323433	-0.873389
C	5.149404	-0.222102	-1.658629
H	5.463390	0.749057	-2.037229
C	5.904519	-1.352952	-1.974472
H	6.796554	-1.257038	-2.588464
C	5.509254	-2.609371	-1.507611
H	6.091268	-3.491743	-1.760553
C	4.358151	-2.729779	-0.727225
H	4.034111	-3.706501	-0.378847
C	3.607058	-1.596375	-0.417248

H	2.691926	-1.694908	0.159741
C	0.692830	0.338502	3.016684
C	-1.059730	-0.773941	2.669894
C	-0.643858	0.519438	3.290264
C	1.963410	0.580744	3.737824
C	-1.279759	-2.010130	3.472940
H	-2.226946	-1.922487	4.025230
H	-0.487044	-2.162171	4.218659
C	-1.409486	1.563490	4.037243
H	-2.269268	1.905546	3.456451
H	-1.792242	1.145637	4.976953
H	1.846637	1.353837	4.507728
H	2.249288	-0.352277	4.244960
H	2.773876	0.846098	3.055344
H	-0.774188	2.423325	4.270199
H	-1.331532	-2.897141	2.836685

MTd 8

E_elec(B3LYP)	-3709.7888848594	G_corr(TPSS)	0.896425

Mo	0.05485454	-0.03065682	0.04326700
Si	-2.60611599	1.84047325	-1.29691877
Si	2.93536063	1.21108635	-1.54884821
Si	-0.37322613	-3.27415439	-1.25026669
O	-1.47563714	0.77988690	-0.76920385
O	1.47848066	0.81319416	-0.91576130
O	0.03502322	-1.76338087	-0.76793592
C	-1.52913091	0.01680904	-3.78136866
C	-0.68455290	1.12854901	-3.86914607
H	-1.10994856	2.12623554	-3.88049029
C	0.70511398	0.97448315	-3.92038777
C	1.24551222	-0.31586719	-3.92027067
H	2.32094247	-0.44742066	-3.97107381
C	0.41969585	-1.44184438	-3.83272794
C	-0.96702067	-1.26435596	-3.78267262
H	-1.61734369	-2.13060503	-3.72650471
C	-2.99901643	0.19079261	-3.67922785
C	-3.58026475	1.02865484	-2.69461687
C	-4.97941574	1.17083648	-2.69569707
H	-5.44795010	1.80054267	-1.94358688
C	-5.78935774	0.50324365	-3.61474135
H	-6.86756374	0.63586972	-3.58761848
C	-5.20634644	-0.34940980	-4.55094044
H	-5.82484746	-0.89136862	-5.26129267

C	-3.82176115	-0.50156426	-4.57821830
H	-3.36164949	-1.15324732	-5.31653524
C	1.59065054	2.16426573	-3.96391858
C	2.65085791	2.33419898	-3.03844153
C	3.46879608	3.46972927	-3.17743063
H	4.28179554	3.62568773	-2.47283175
C	3.25011470	4.42025572	-4.17494836
H	3.90169982	5.28627730	-4.25436182
C	2.17874504	4.26020257	-5.05242698
H	1.98362391	5.00125610	-5.82290604
C	1.35790628	3.13985713	-4.94286389
H	0.53063509	3.00321170	-5.63455732
C	1.00836867	-2.80280970	-3.78398207
C	0.67006319	-3.72073677	-2.75813211
C	1.23953252	-5.00548983	-2.81200797
H	1.00208404	-5.72285649	-2.03053619
C	2.12583201	-5.37911120	-3.82257511
H	2.54744494	-6.38058807	-3.83444849
C	2.48084549	-4.45212495	-4.80138721
H	3.18462721	-4.72149688	-5.58433091
C	1.92441784	-3.17510814	-4.77721123
H	2.18627551	-2.45410559	-5.54733745
C	-3.80582649	2.20225419	0.10395797
C	-4.57432223	1.16680675	0.66673888
H	-4.50612465	0.16301465	0.25468766
C	-5.42187122	1.39876498	1.74843450
H	-6.00475287	0.58281853	2.16803920
C	-5.51824082	2.68148690	2.29483219
H	-6.17715821	2.86596364	3.13911579
C	-4.76742193	3.72425898	1.75103918
H	-4.83987369	4.72382796	2.17189743
C	-3.92120238	3.48350527	0.66610036
H	-3.33822902	4.30312814	0.25251861
C	-1.80039285	3.45172027	-1.81768105
C	-2.48430361	4.34493619	-2.66120262
H	-3.48181059	4.09336398	-3.01692576
C	-1.89963050	5.54458248	-3.06764383
H	-2.44219687	6.21850053	-3.72558232
C	-0.61258464	5.87276466	-2.63627881
H	-0.14814691	6.79992977	-2.96096467
C	0.08121113	4.99988076	-1.79749342
H	1.08933279	5.24638630	-1.47679188
C	-0.50860274	3.80216691	-1.39282085
H	0.05415862	3.11901434	-0.76238758
C	3.91157501	2.18544698	-0.27202177
C	3.42330670	3.41350273	0.21049912
H	2.50031796	3.82039891	-0.19499050

C	4.09665142	4.12125795	1.20440284
H	3.69941549	5.06734566	1.56316809
C	5.28152979	3.61055844	1.74147897
H	5.80879044	4.15894378	2.51755286
C	5.78575943	2.39558835	1.27624836
H	6.70742758	1.99480459	1.69026527
C	5.10538194	1.69321561	0.27883117
H	5.50597799	0.74568551	-0.07385453
C	3.90624507	-0.33307401	-1.98365910
C	4.98290802	-0.26140315	-2.88515952
H	5.24590872	0.69411657	-3.33535475
C	5.71274020	-1.39943632	-3.22933374
H	6.53756093	-1.32425114	-3.93322921
C	5.37462149	-2.63656540	-2.67662363
H	5.93159845	-3.52790312	-2.95242798
C	4.31011600	-2.72731188	-1.77924794
H	4.03422604	-3.69240987	-1.36401448
C	3.58507091	-1.58583190	-1.43664451
H	2.74081577	-1.67645369	-0.75849006
C	0.04842404	-4.48988210	0.11986508
C	1.37905199	-4.64314648	0.55039176
H	2.17117570	-4.08849133	0.05362291
C	1.70601731	-5.49065011	1.60731250
H	2.74099117	-5.59169646	1.92389508
C	0.70046428	-6.20745910	2.26167049
H	0.95141120	-6.86840162	3.08701516
C	-0.62589002	-6.07292198	1.85008300
H	-1.41137742	-6.62907616	2.35526595
C	-0.94493631	-5.22233032	0.78899131
H	-1.98251032	-5.12311229	0.47870286
C	-2.21528246	-3.37793688	-1.58620058
C	-2.73497138	-4.41895385	-2.37566110
H	-2.06149822	-5.16196325	-2.79899946
C	-4.10124459	-4.50843106	-2.64315446
H	-4.48248501	-5.31716193	-3.26125830
C	-4.97632615	-3.55156417	-2.12448131
H	-6.03945201	-3.60987483	-2.34152932
C	-4.47950853	-2.51200619	-1.33756650
H	-5.15692349	-1.75661761	-0.94963633
C	-3.11269897	-2.42836273	-1.07157044
H	-2.73658964	-1.59787055	-0.48029559
C	0.08227902	-0.81941960	1.95269496
C	-0.55239262	0.50402755	1.94442377
C	0.90937365	0.39084229	1.87671305
C	0.01839878	-2.06484520	2.76457531
C	-1.56204156	1.25070550	2.74282994
H	-1.31524406	1.20645471	3.81221819

H	-2.56322755	0.83319420	2.60359915
C	2.09506705	0.95717598	2.57499580
H	2.22705506	2.01582353	2.33435694
H	1.98148099	0.86215585	3.66335773
H	0.85962096	-2.72655449	2.53962023
H	0.04389821	-1.82634739	3.83645155
H	-0.90304613	-2.61934434	2.56039957
H	3.01041431	0.43390999	2.28075844
H	-1.59772374	2.30328496	2.44438976

Minimum energy paths of isomerization

These data are obtained with the NEB method. The highest points were taken as starting points for the TS optimizations and are thus only close to but not exactly like the TS's. If no TS could be converged, these highest energy points were taken as approximations for the TS. For the x-axis, the distances of each point to its predecessor, i.e., $||\boldsymbol{R}(i+1)-\boldsymbol{R}(i)||$, where $\boldsymbol{R}(i)$ are the *xyz*-coordinates of the *i*-th point, are cumulatively summed up. This resembles the total movement on the 3N-6 dimensional potential energy surface (since the translational and rotational degrees of freedom are projected out in the NEB implementation of ORCA).

MCBD formation

We found that the MCBD formation occurs in two steps. First, the alkyne has to get close to the metal-carbon triple bond by squeezing between two of the free phenyl groups. A loosely bound van-der-Waals complex is obtained. This complex reacts further to give the MCBD via the commonly known mechanism, see Ref [16] or original manuscript. Since the latter barrier is more meaningful for comparison to the high barrier dissociation (especially for the dissociation), we used the second TS as TS1. For all energy profiles, the energy of the initial structure, from where the vdW complex is formed, is set to be the energy reference.

Formation of the vdW complex:

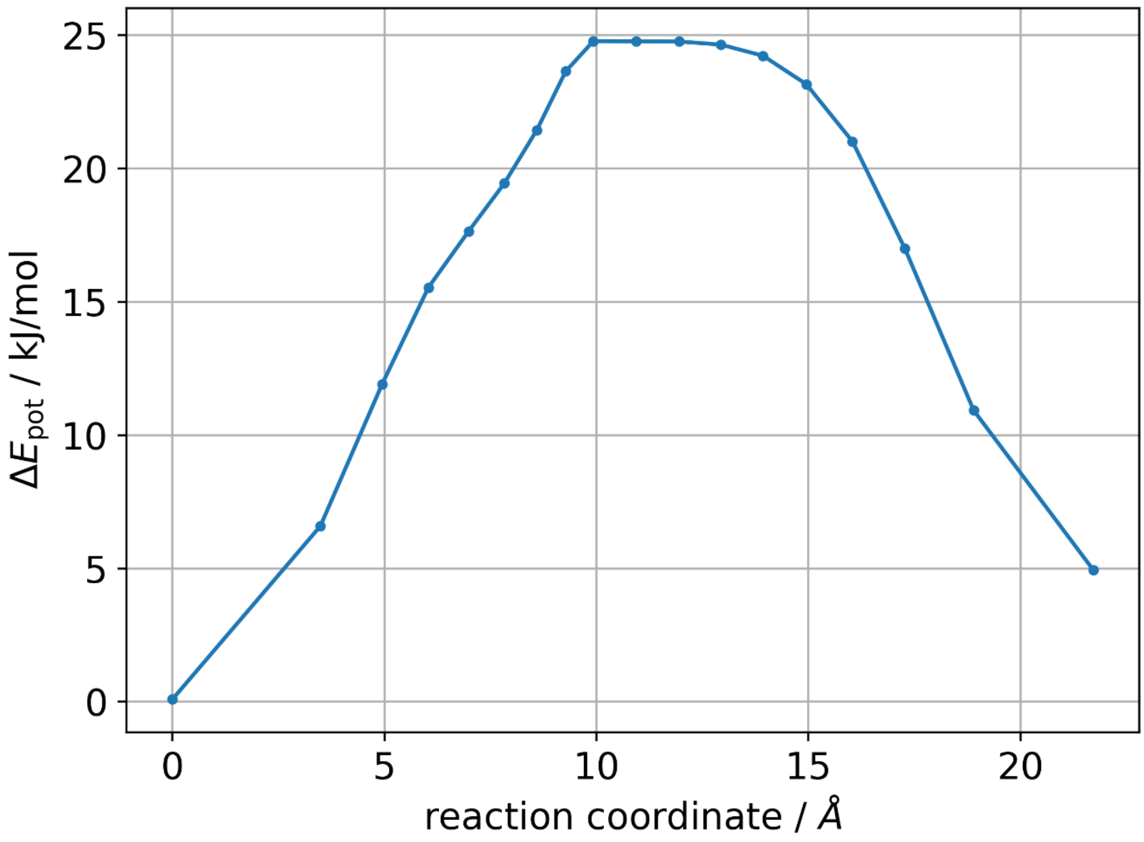

Actual MCBD 9 formation (TS1):

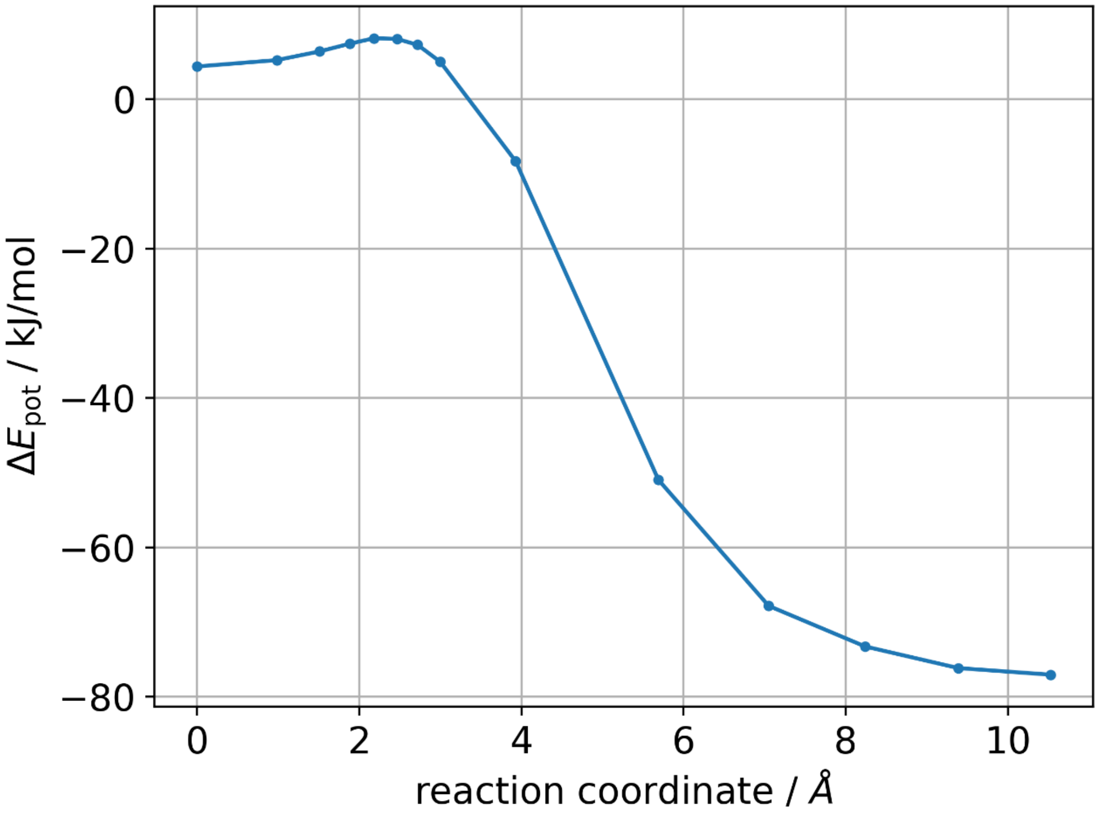

MCBD **9** to Intermediate **12**

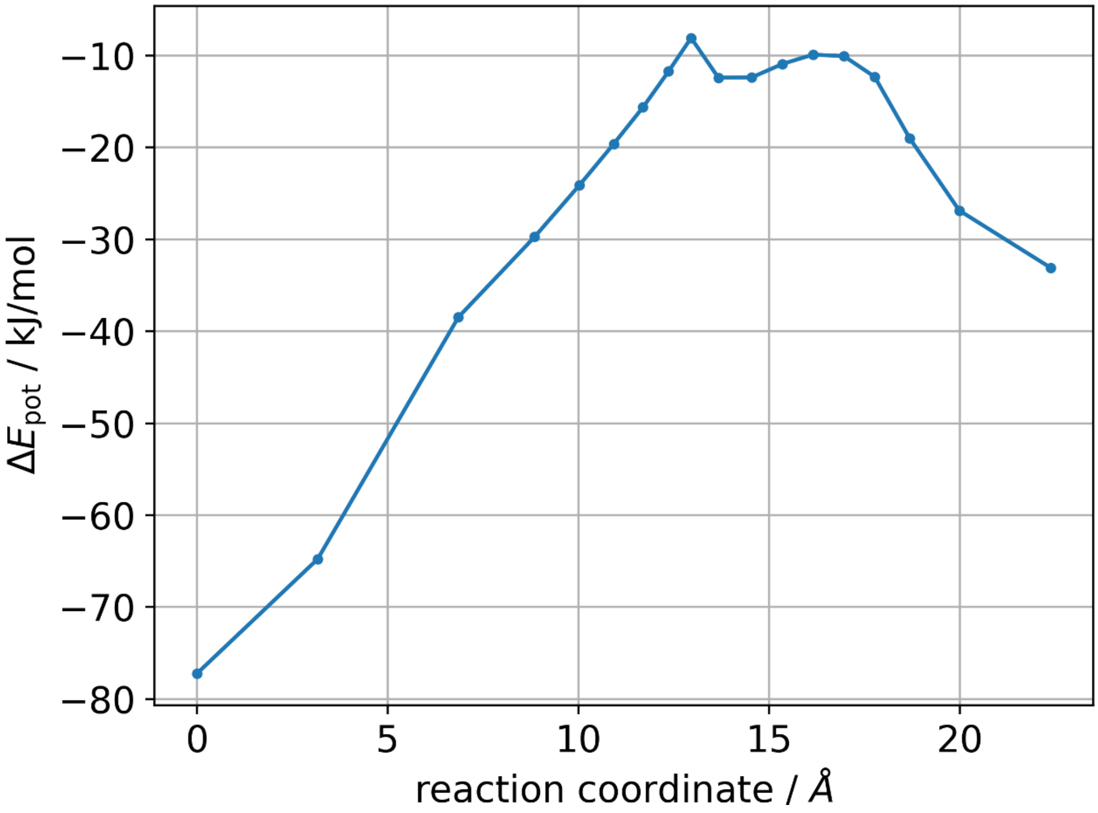

Berry Pseudorotation **12** to **12'**

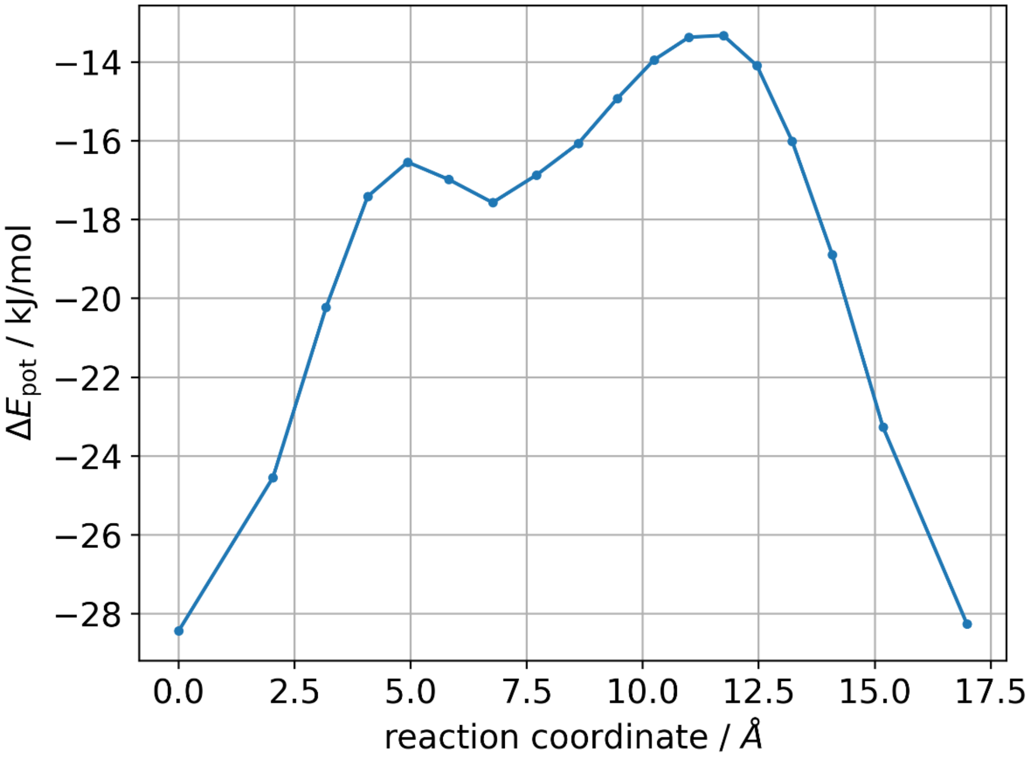

Intermediate **12** to MTd **8**

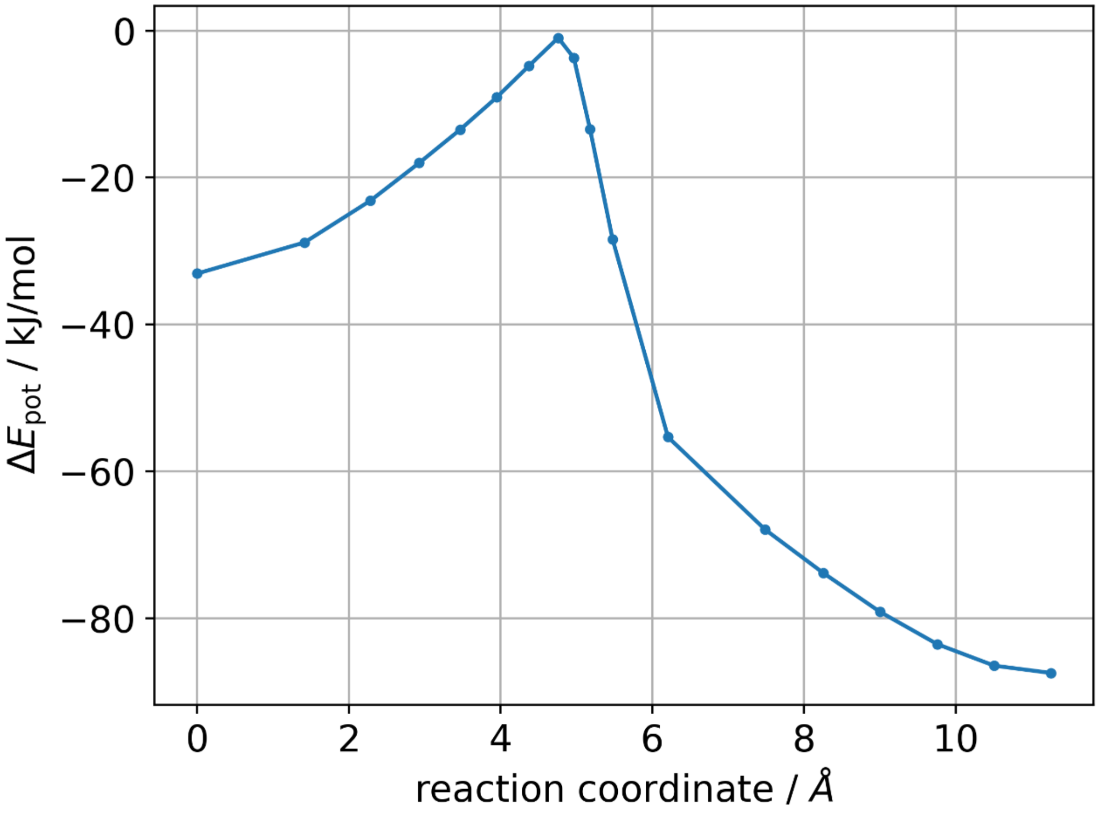

High barrier dissociation (**TS1'**)

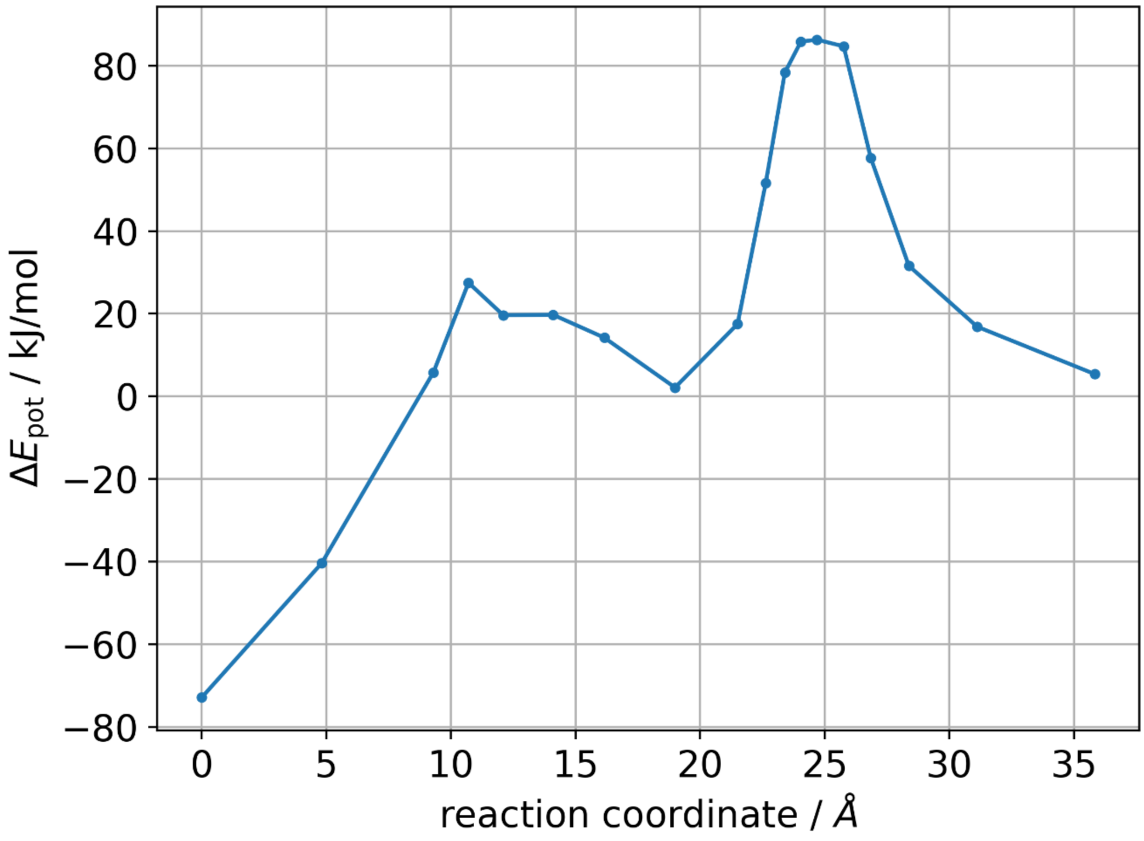

NMR calculation

Calculated ^{1}H NMR isotropic shieldings σ_{iso} and shifts δ ($\sigma_{ref} = 32.064$ ppm) of the protons in the MCBD unit in **9**. Note that $C_{\alpha'}$ is more shielded than C_α due to the adjacent Ph-groups.

	δ_{exp} / ppm		calc σ_{iso} / ppm	calc δ / ppm
C_α	2.42		29.466	
			29.386	
			27.635	
		av	28.829	3.24
$C_{\alpha'}$	1.18		30.333	
			30.690	
			31.629	
		av	30.884	1.18
C_β	1.71		28.898	
			28.051	
			28.823	
		av	28.591	3.47

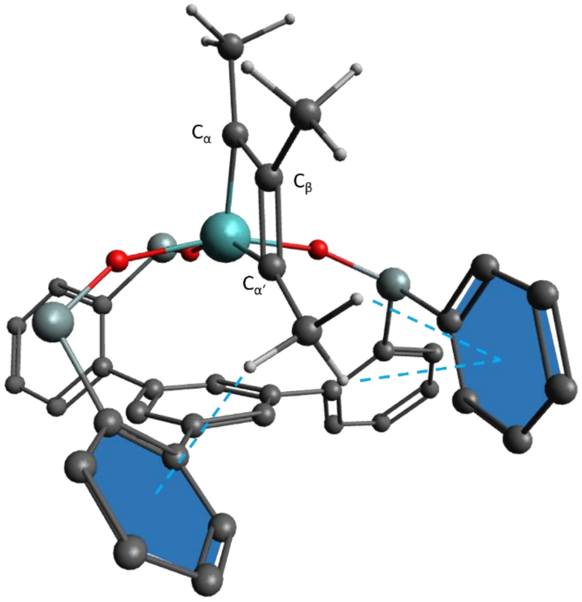

SPECTRA

VT ^{1}H NMR Studies of Metallacyclobutadiene Complex 7, 600 MHz, $C_6D_5CD_3$, 25 °C to −40 °C

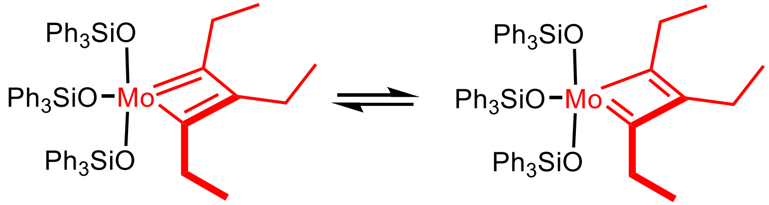

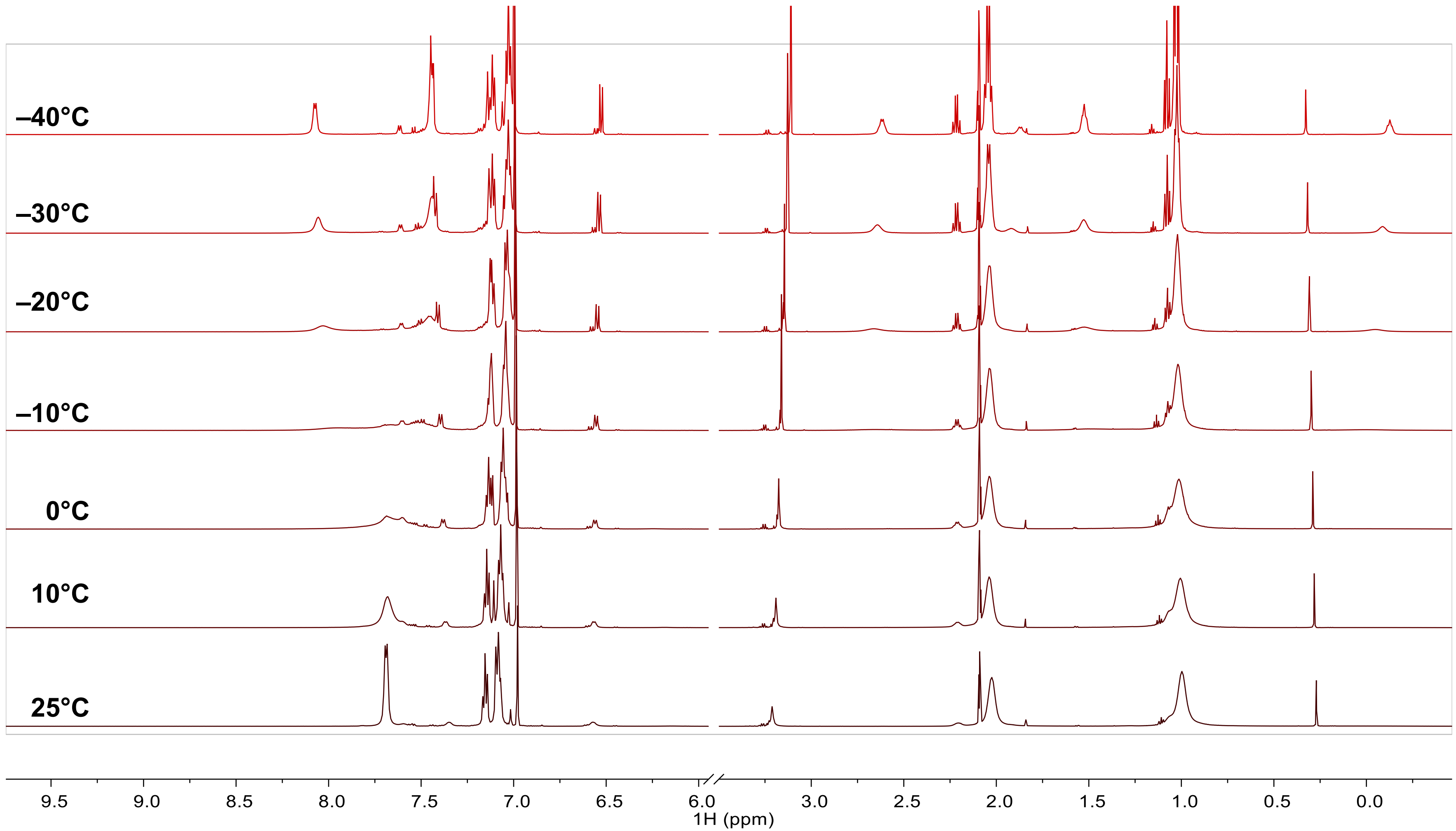

^{1}H NMR Spectrum of Metallacyclobutadiene Complex 7, 600 MHz, $C_6D_5CD_3$, –40 °C

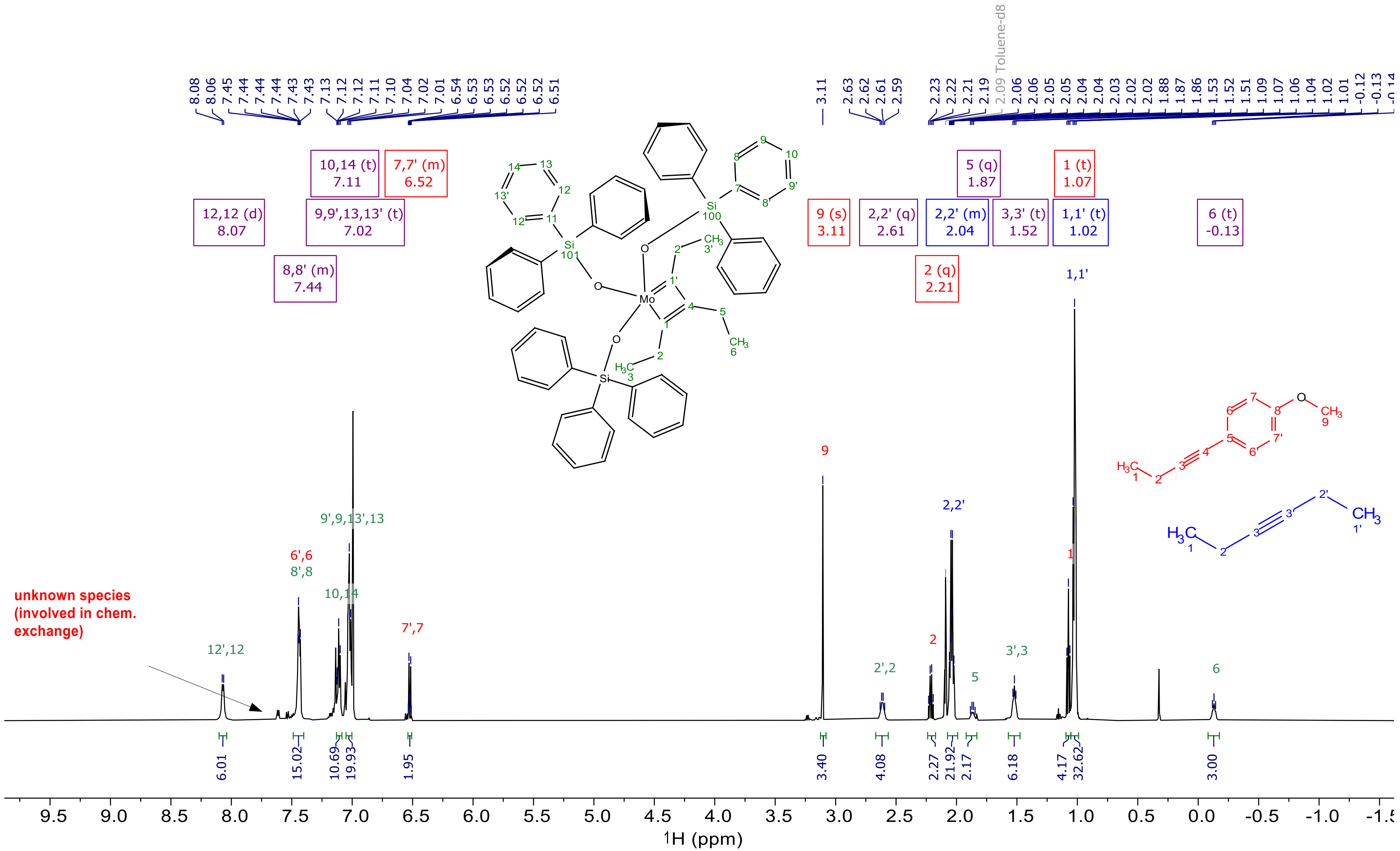

^{1}H CLIP-COSY NMR Spectrum of Metallacyclobutadiene Complex 7, 600 MHz, $C_6D_5CD_3$, –40 °C

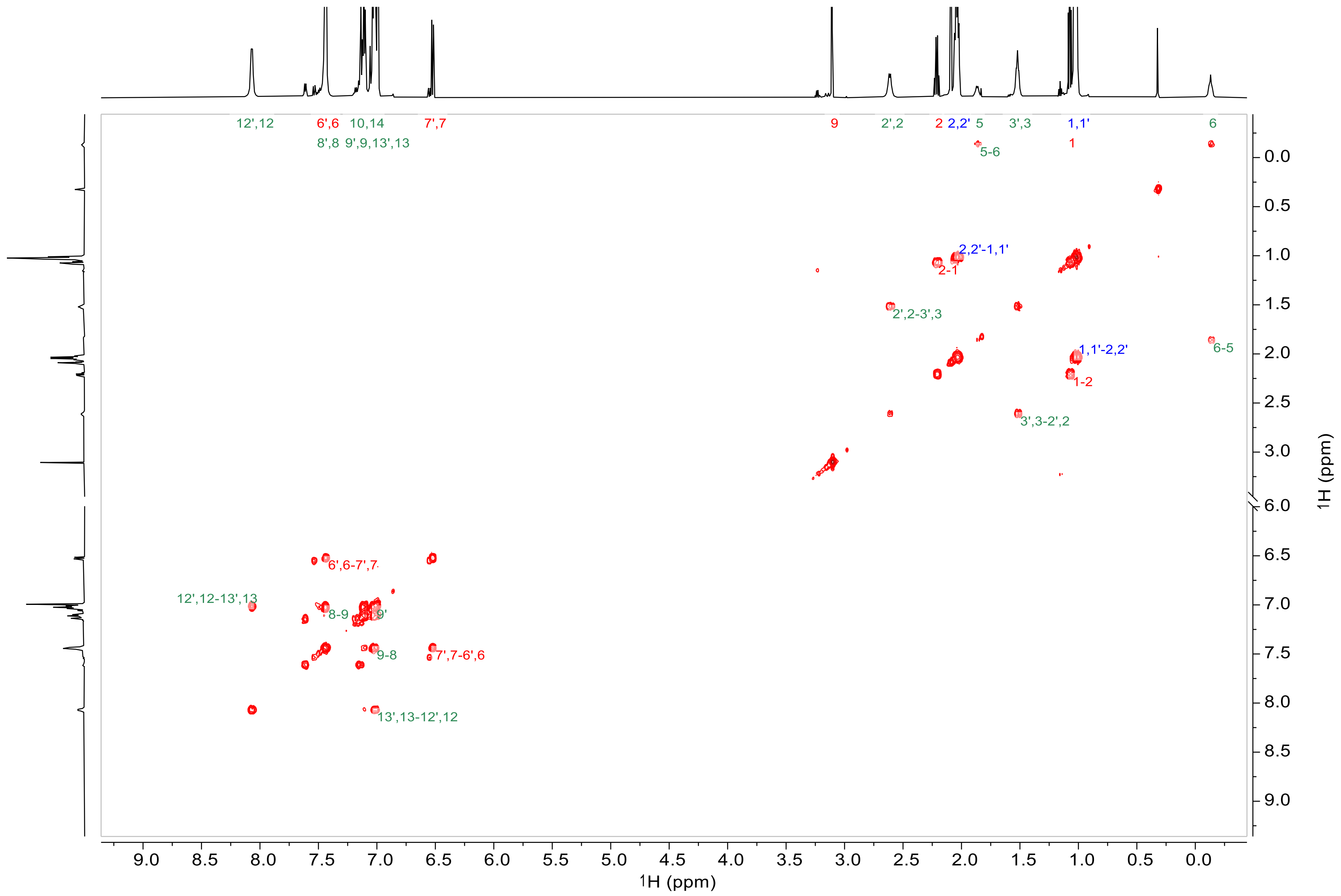

^{1}H EASY-ROESY NMR Spectrum ($\tau_{spinlock}$= 200ms) of Metallacyclobutadiene Complex 7, 600 MHz, $C_6D_5CD_3$, –40 °C

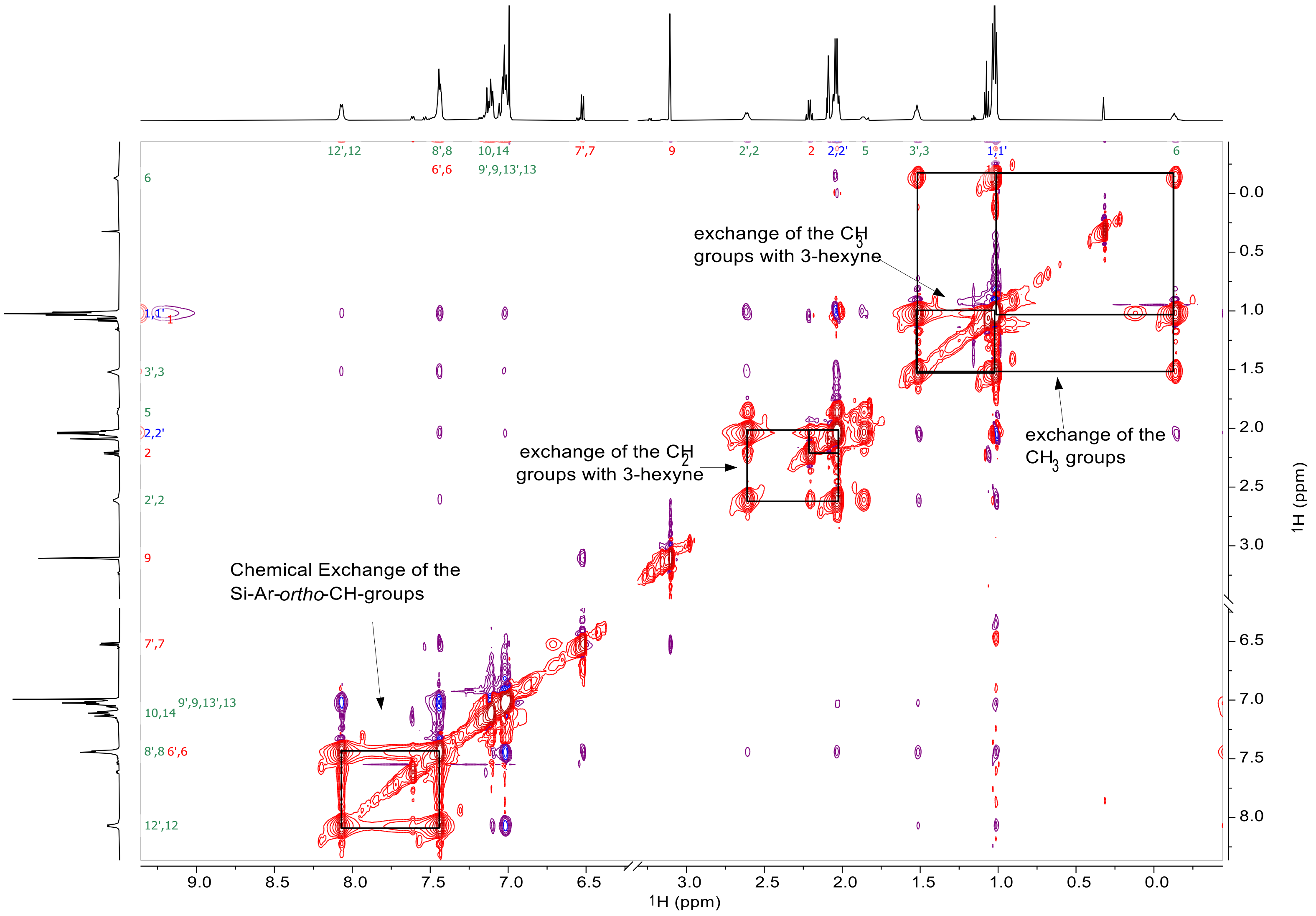

^{13}C NMR Spectrum of Metallacyclobutadiene Complex 7, 151 MHz, $C_6D_5CD_3$, –40 °C

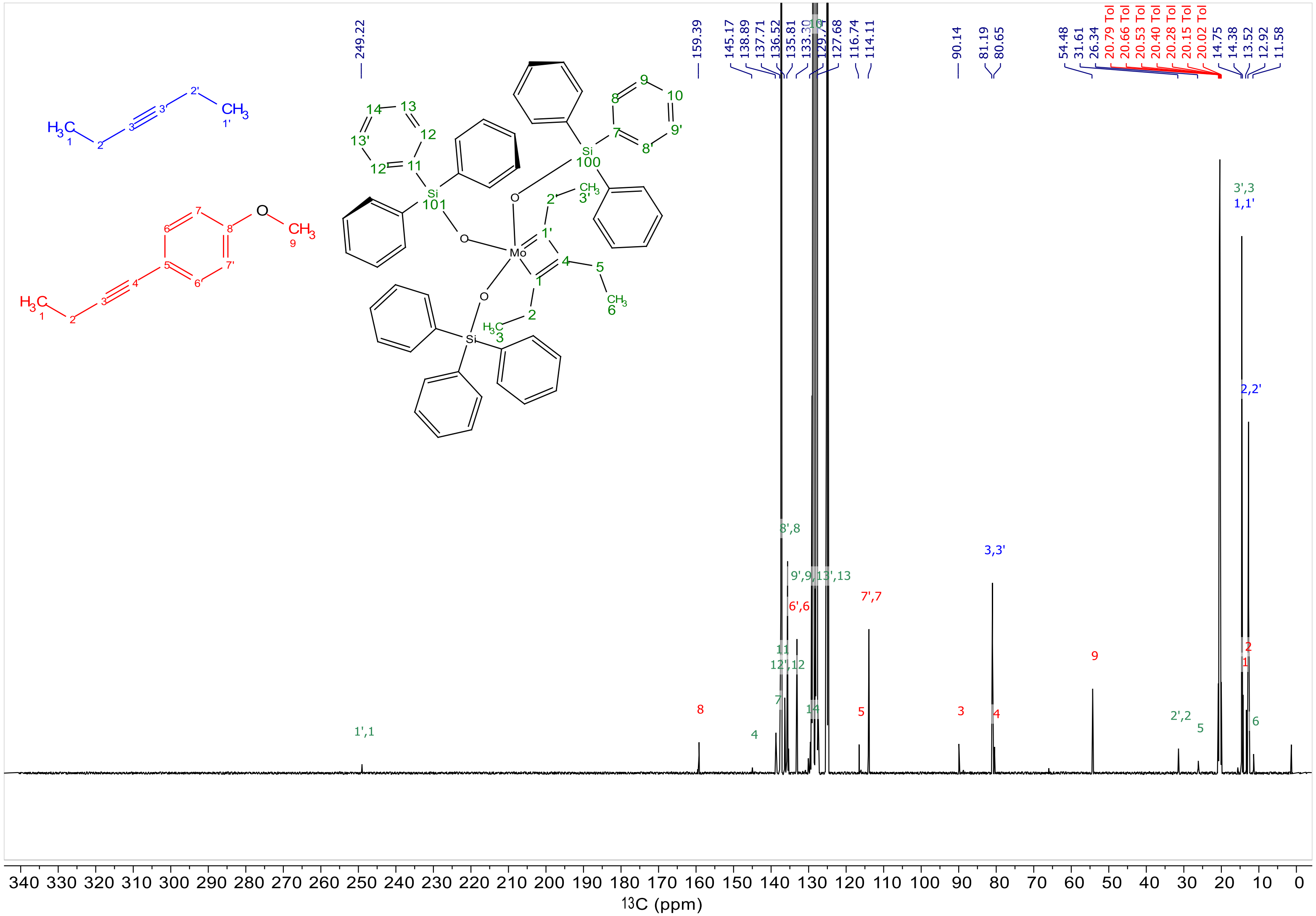

^{1}H-^{13}C edited HSQC NMR Spectrum of Metallacyclobutadiene Complex 7, $C_6D_5CD_3$, −40 °C

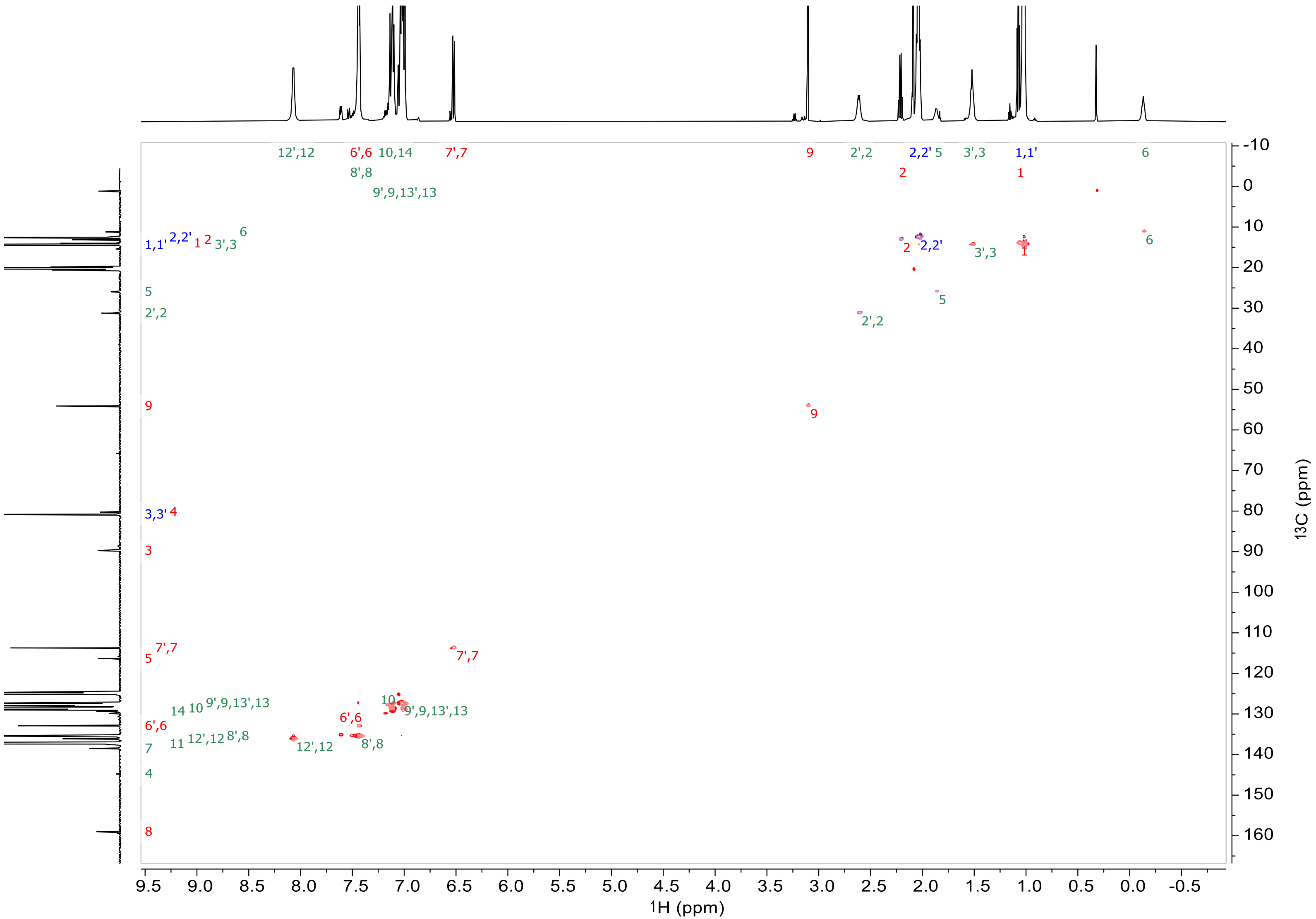

^{1}H-^{13}C HMBC NMR Spectrum of Metallacyclobutadiene Complex 7, $C_6D_5CD_3$, –40 °C

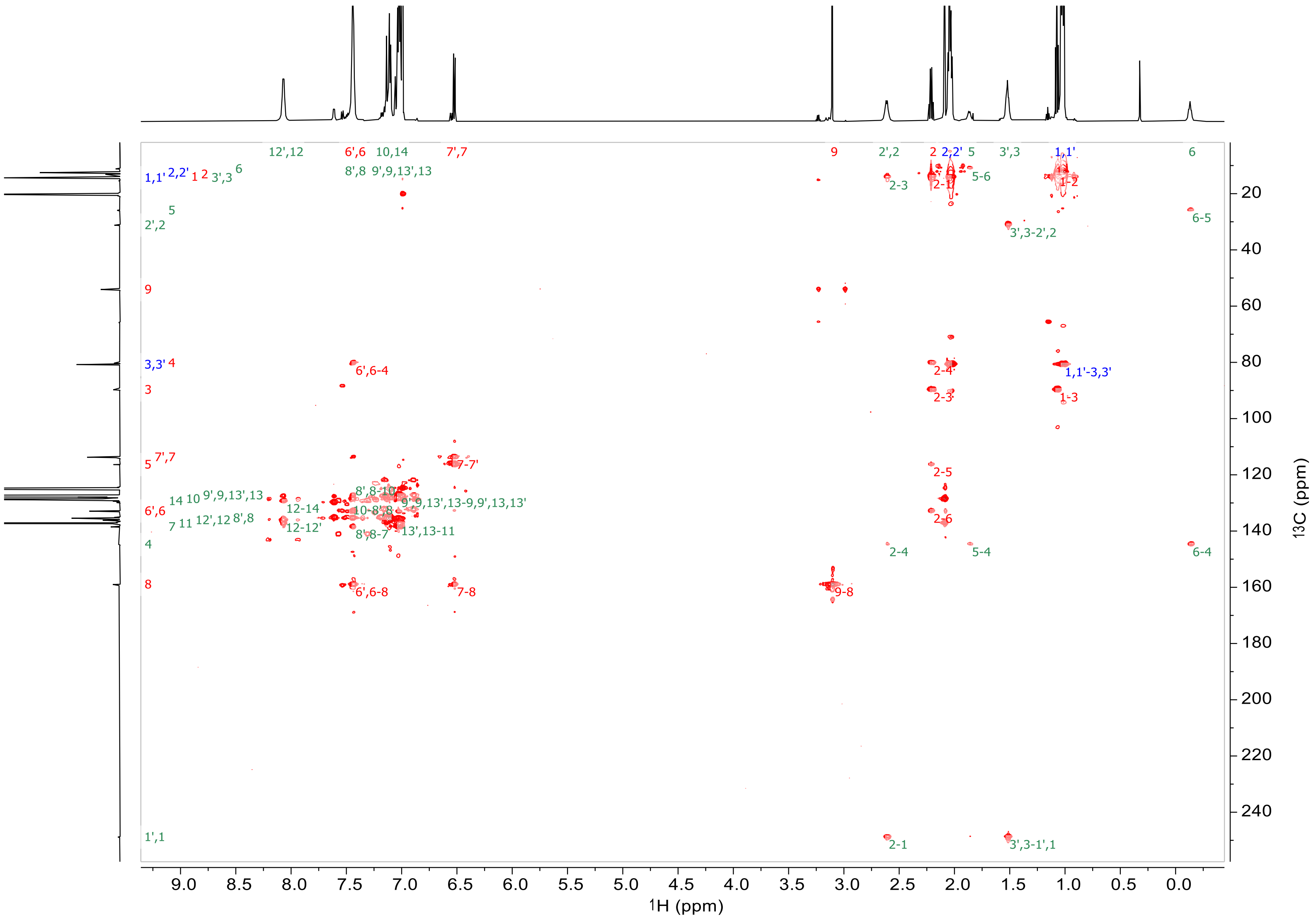

Aromatic ^{1}H region of the ^{1}H-^{13}C HMBC NMR Spectrum of Metallacyclobutadiene Complex 7, $C_6D_5CD_3$, –40 °C

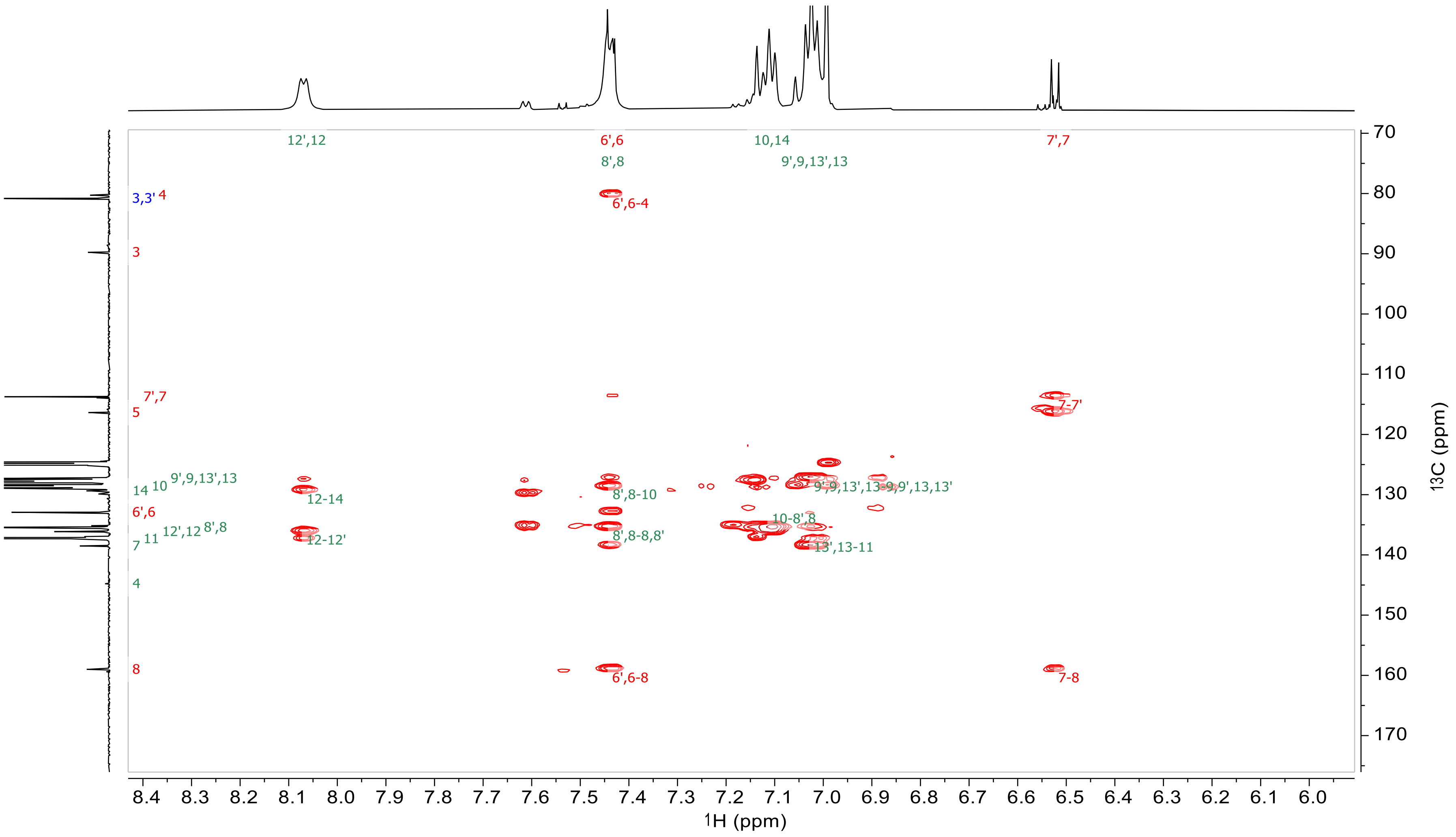

^{29}Si-INEPT NMR Spectrum of Metallacyclobutadiene Complex 7, 119 MHz, $C_6D_5CD_3$, –40 °C

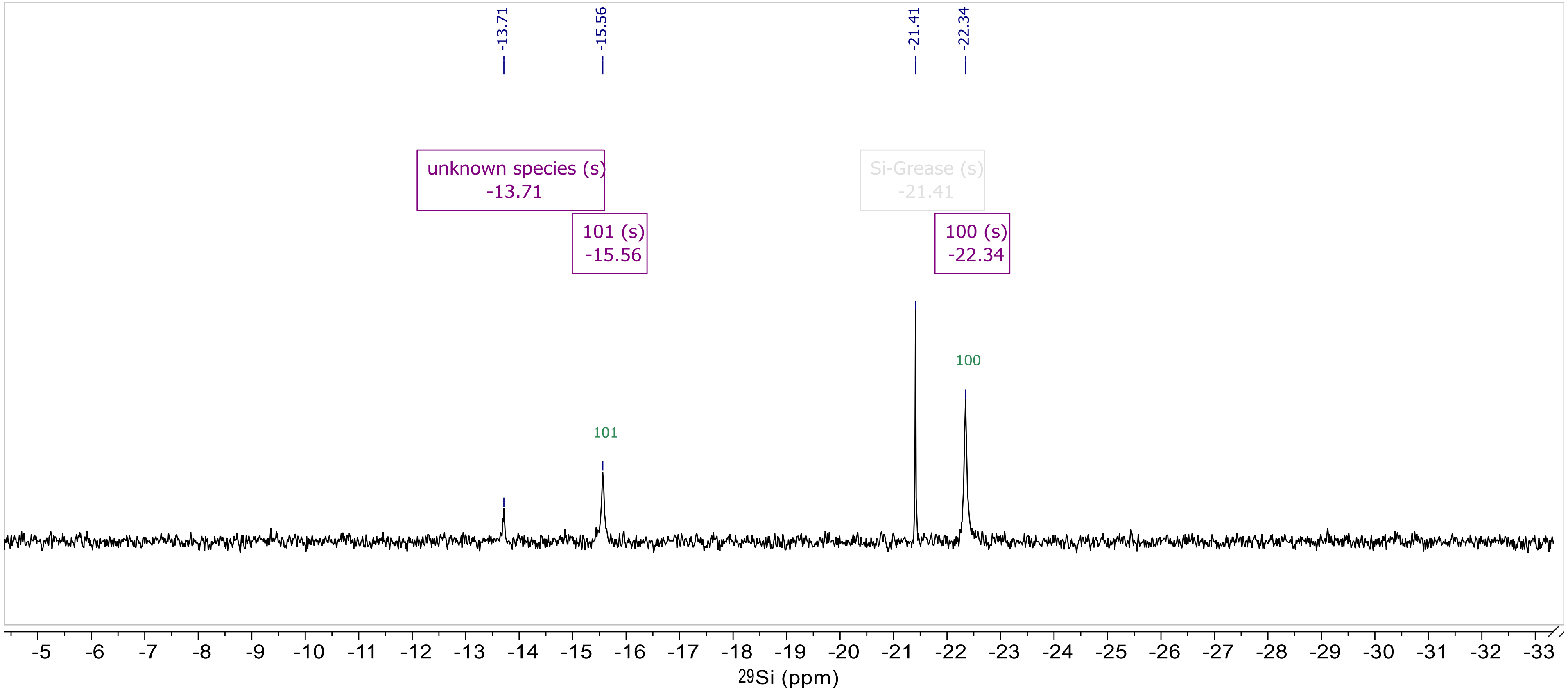

^{1}H-^{29}Si HMBC NMR Spectrum of Metallacyclobutadiene Complex 7, $C_6D_5CD_3$, –40 °C

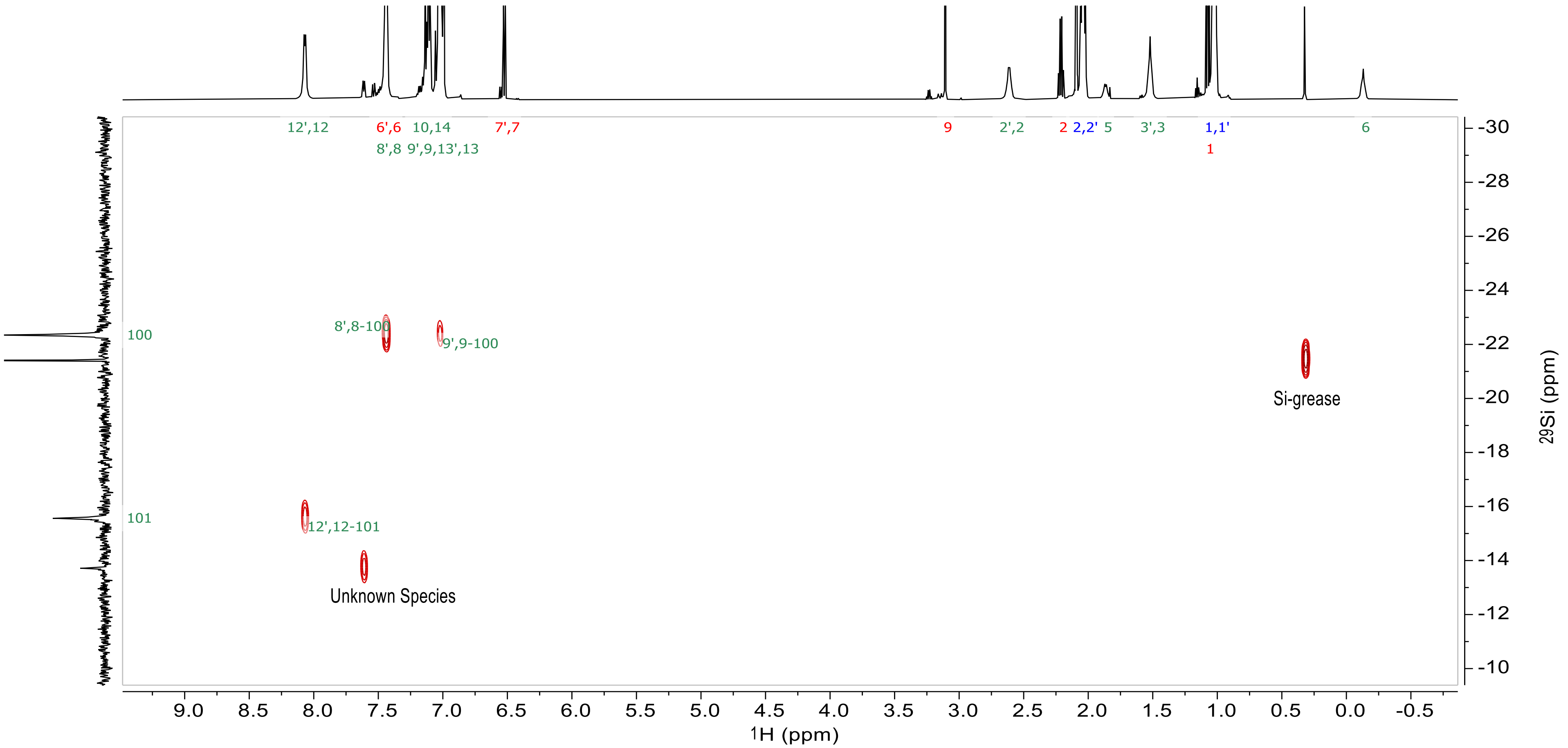

Comparison of ^{1}H NMR of Metallacyclobutadiene Complex 7 at -90°C (400 MHz) and at -40 °C (600 MHz), $C_6D_5CD_3$

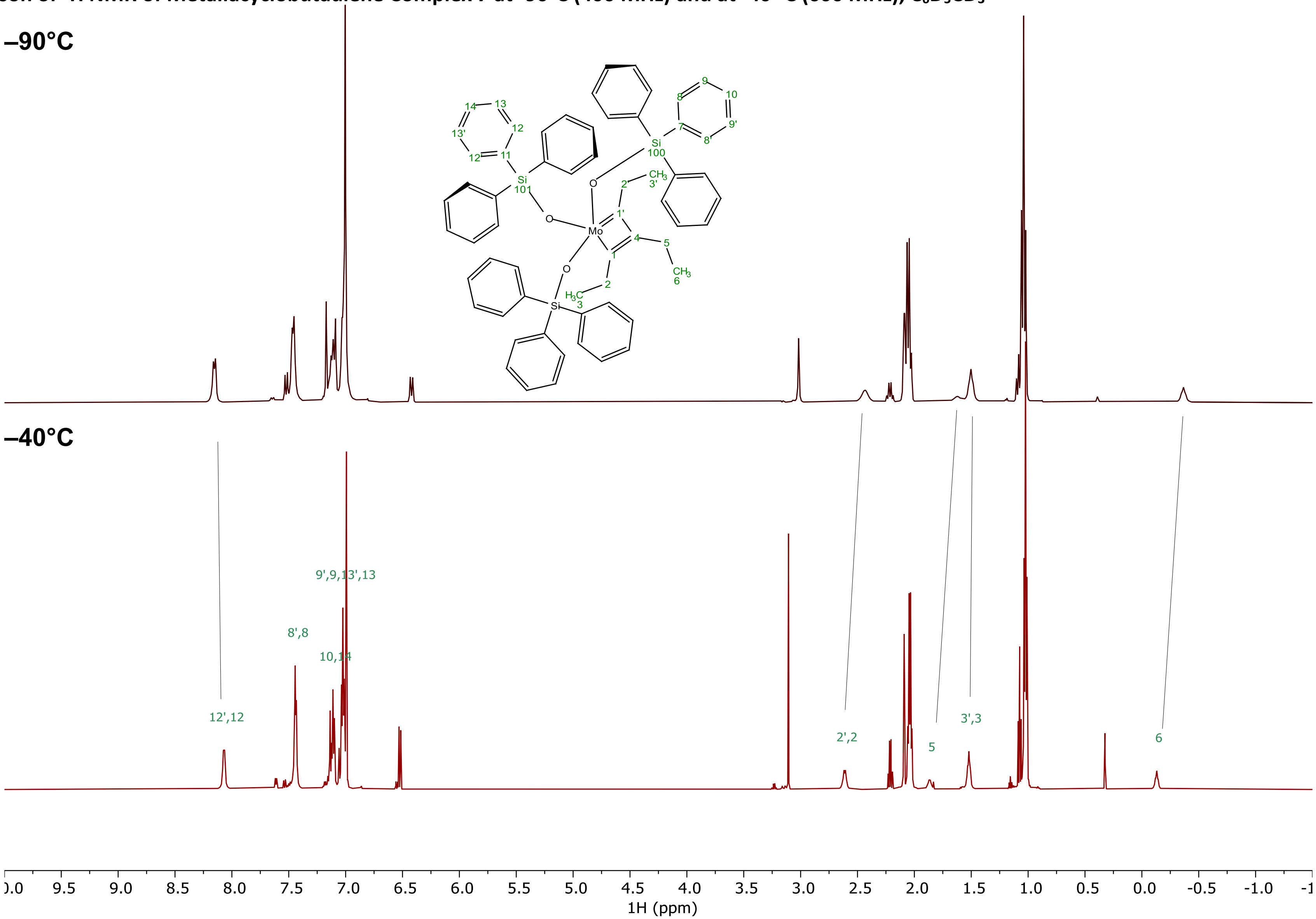

Comparison of ^{13}C NMR of Metallacyclobutadiene Complex 7 at -40 °C (151 MHz) and at -90°C (101 MHz), $C_6D_5CD_3$

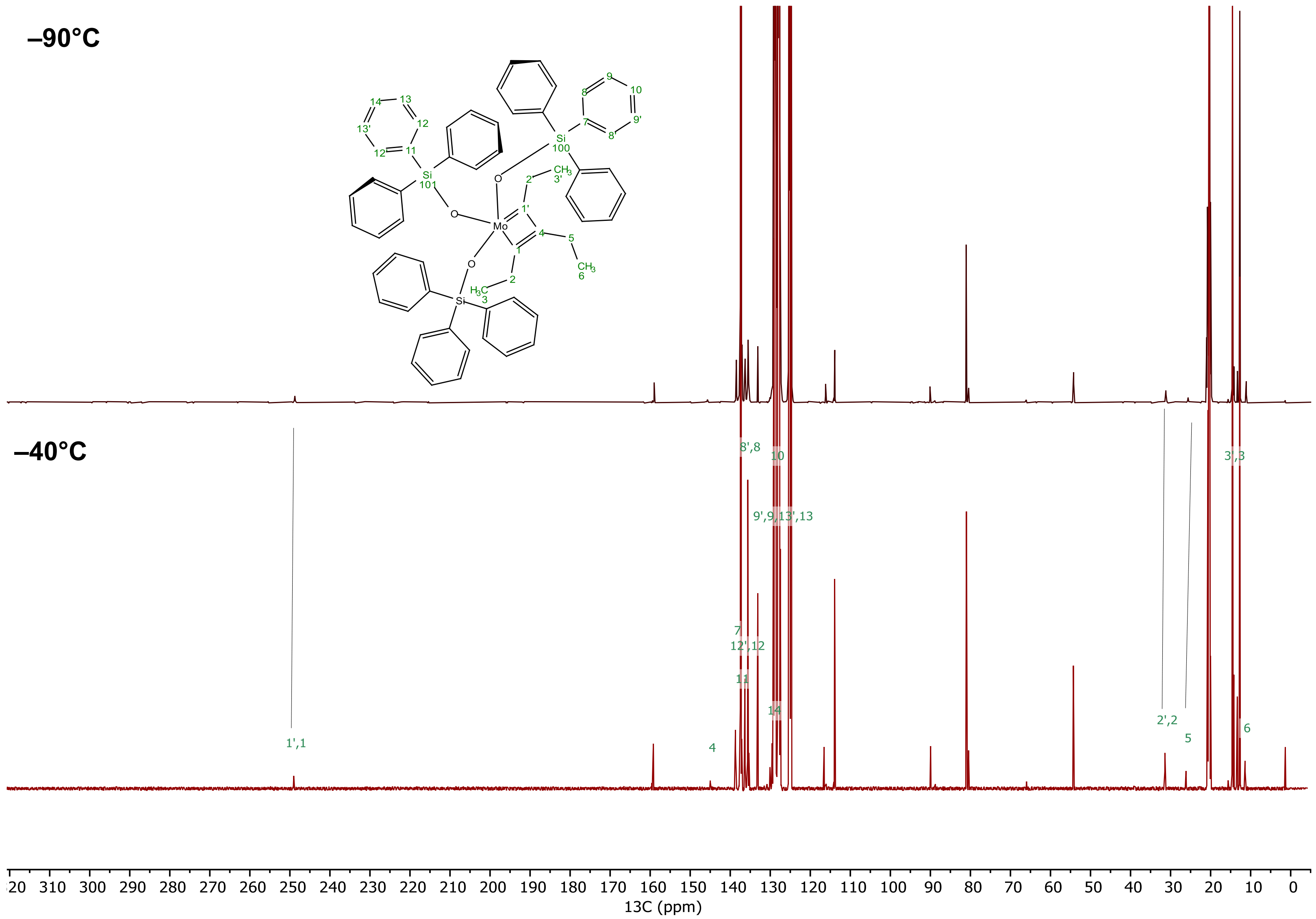

VT ¹H NMR Studies of Complexes 8 and 9, 600 MHz, $C_6D_5CD_3$, 25 °C to –40 °C

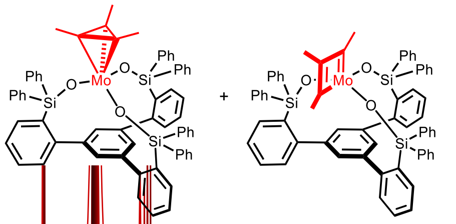

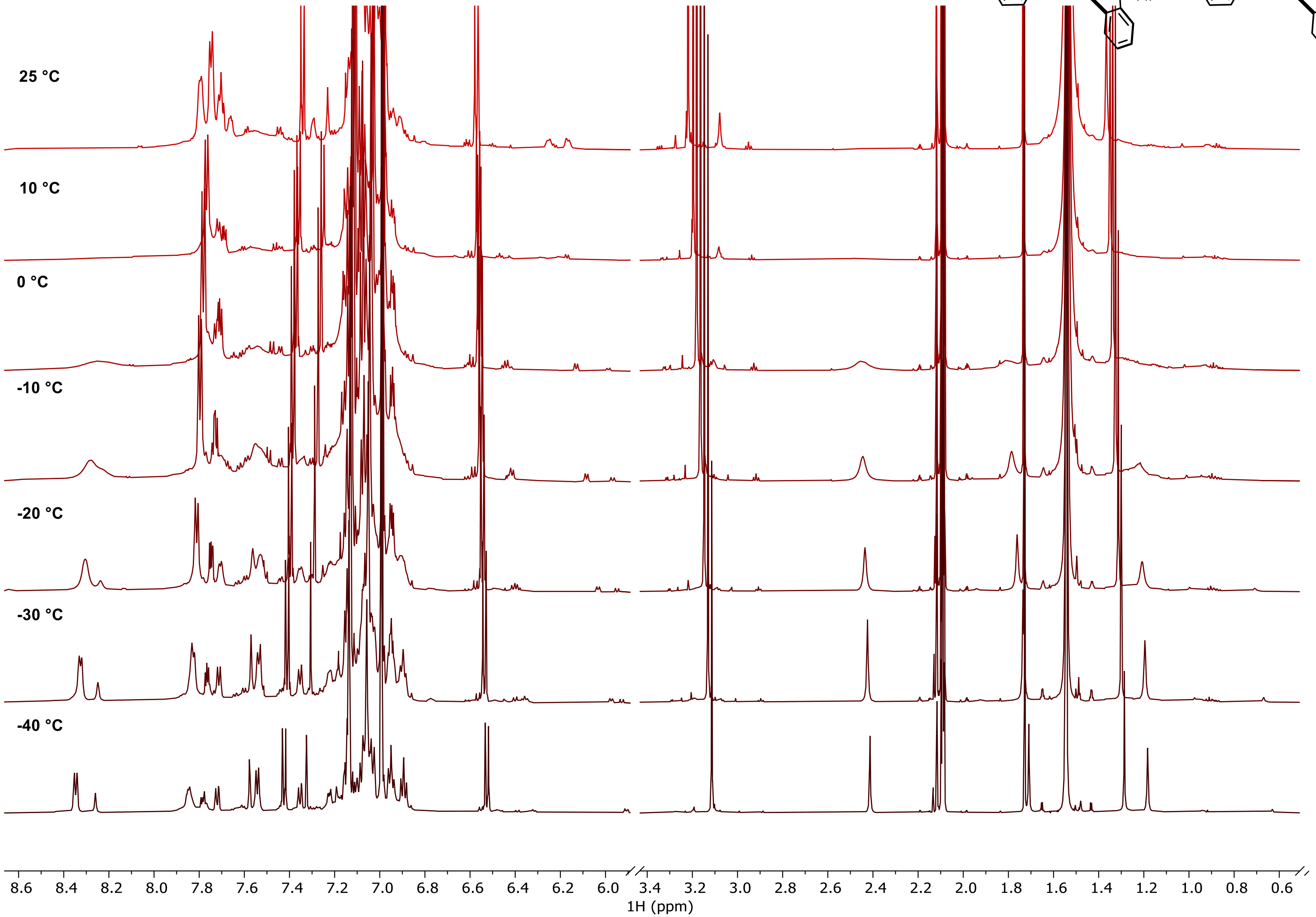

^{1}H NMR Spectrum of Complexes 8 and 9, 600 MHz, $C_6D_5CD_3$, –40 °C

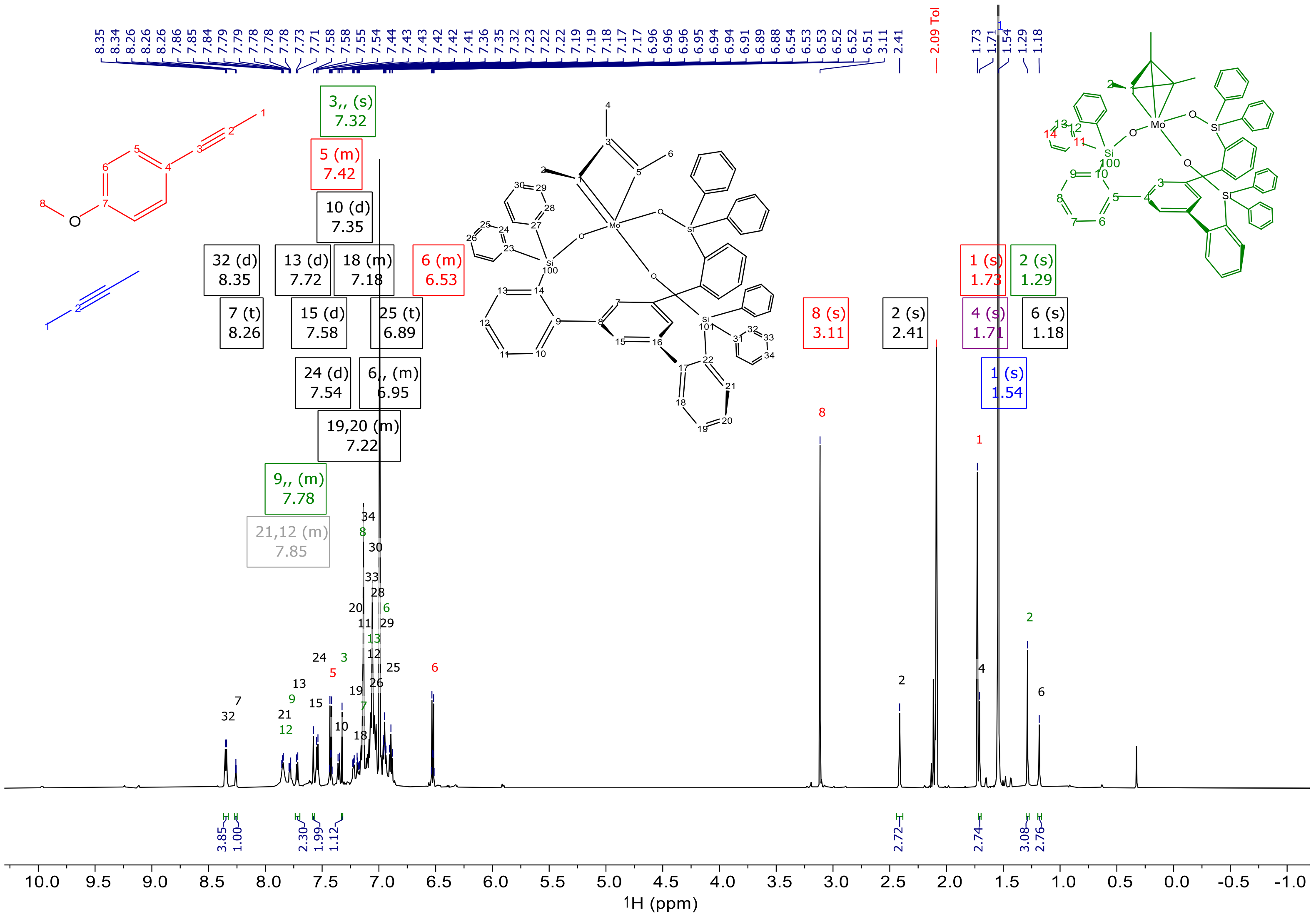

Aromatic Region of the ¹H NMR Spectrum of Complexes 8 and 9, 600 MHz, $C_6D_5CD_3$, –40 °C

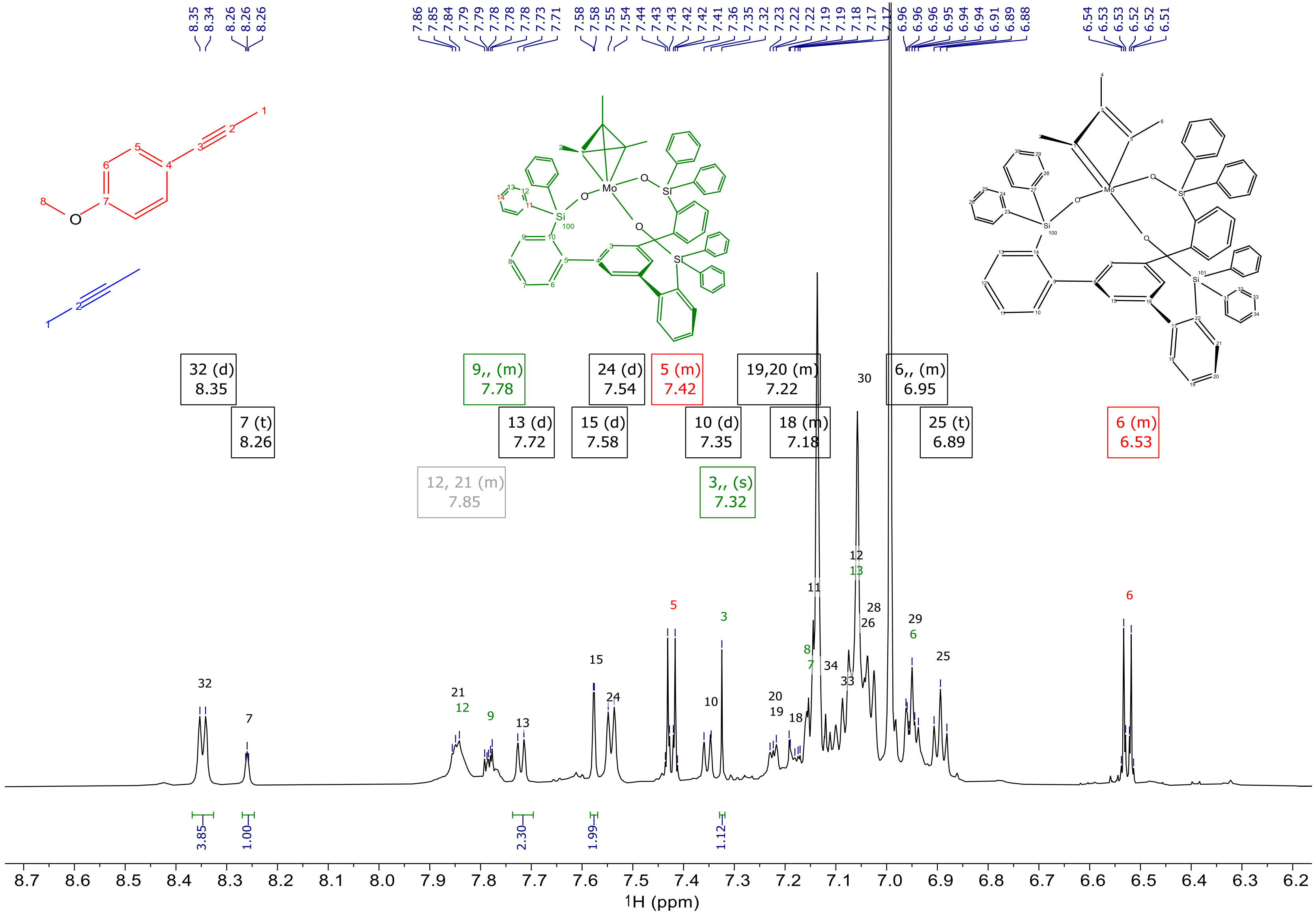

Aliphatic Region of the ^{1}H NMR Spectrum of Complexes 8 and 9, 600 MHz, $C_6D_5CD_3$, −40 °C

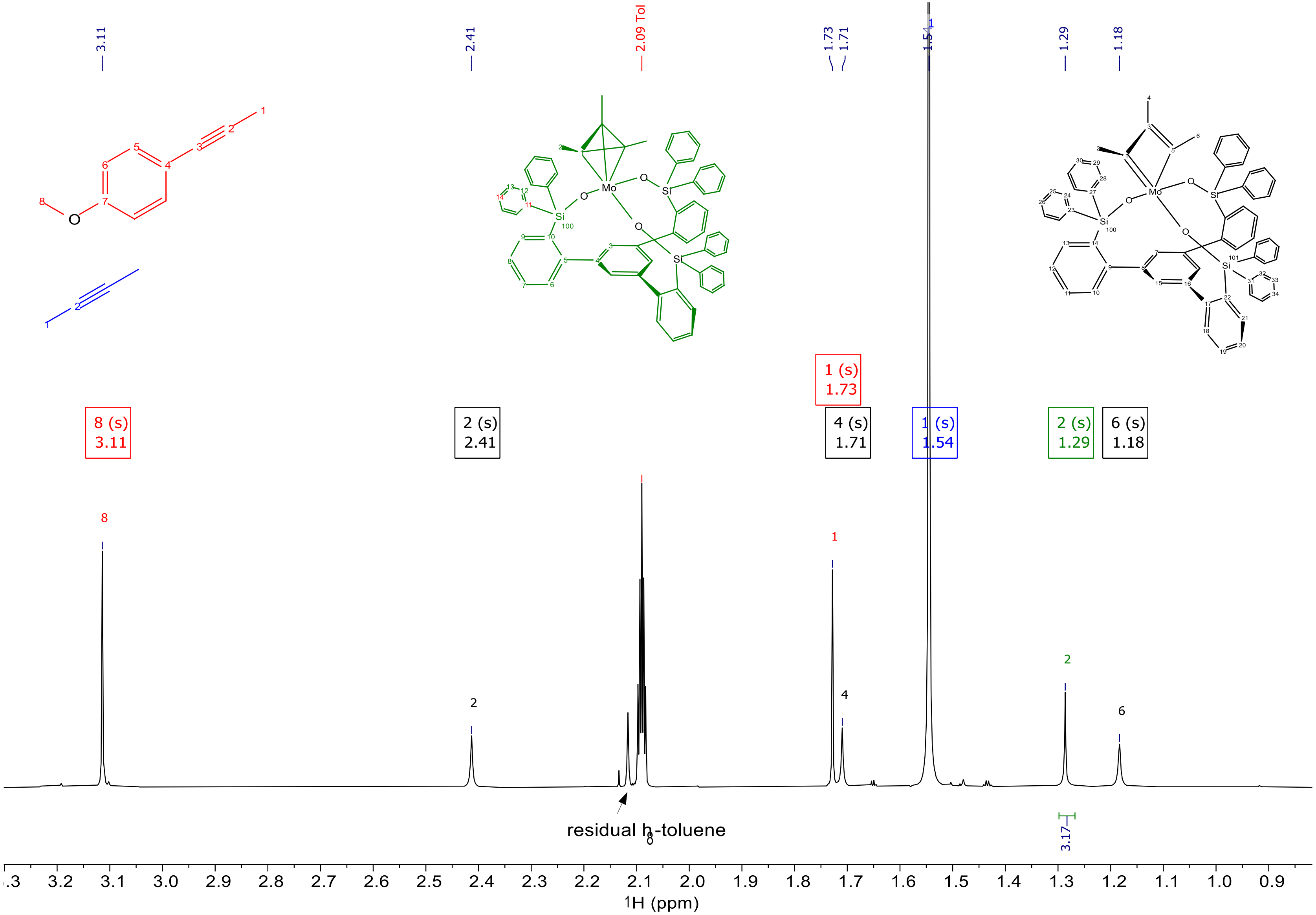

^{1}H COSY NMR Spectrum of Complexes 8 and 9, 600 MHz, $C_6D_5CD_3$, –40 °C

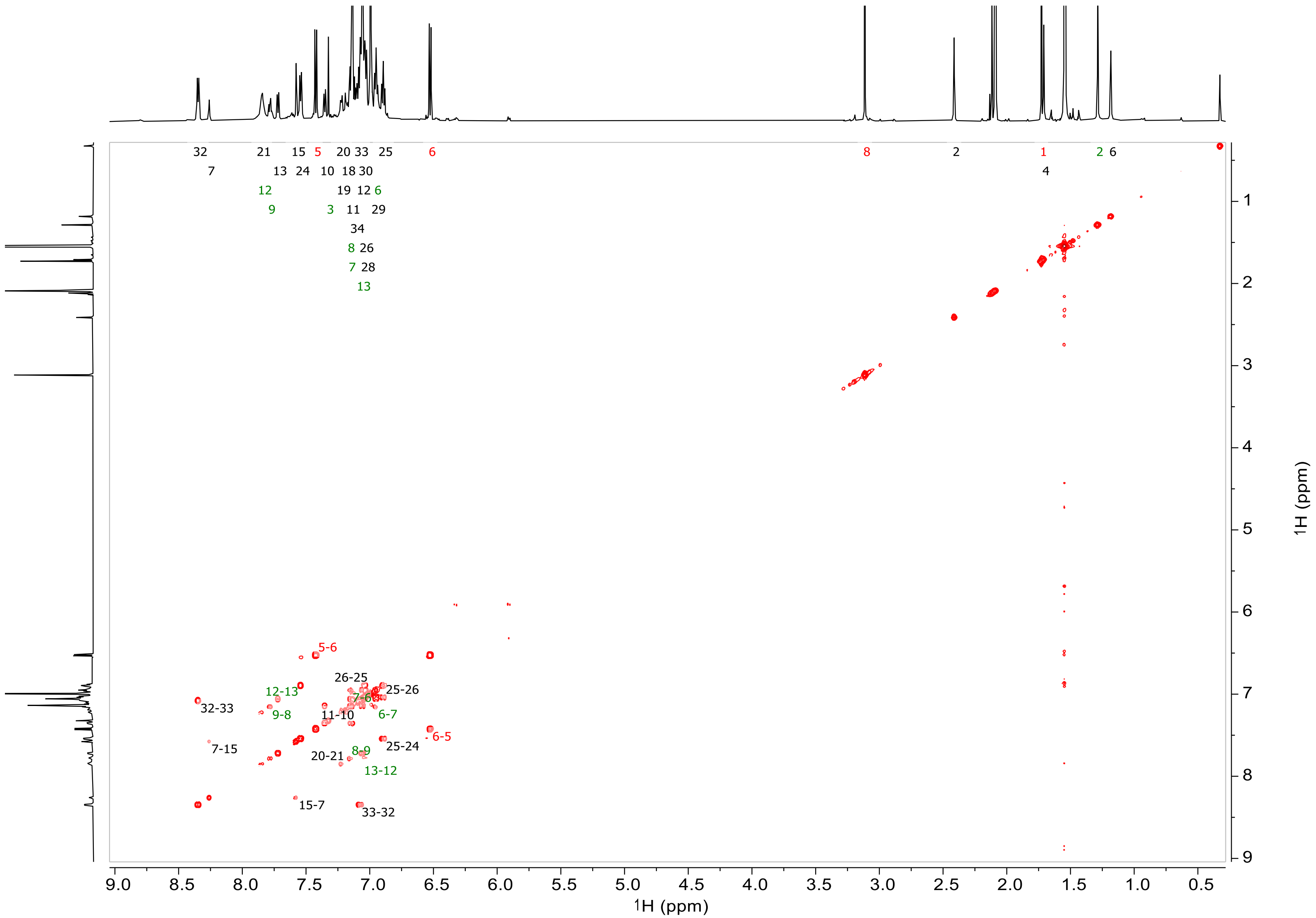

^{13}C NMR Spectrum of Complexes 8 and 9, 151 MHz, $C_6D_5CD_3$, –40 °C

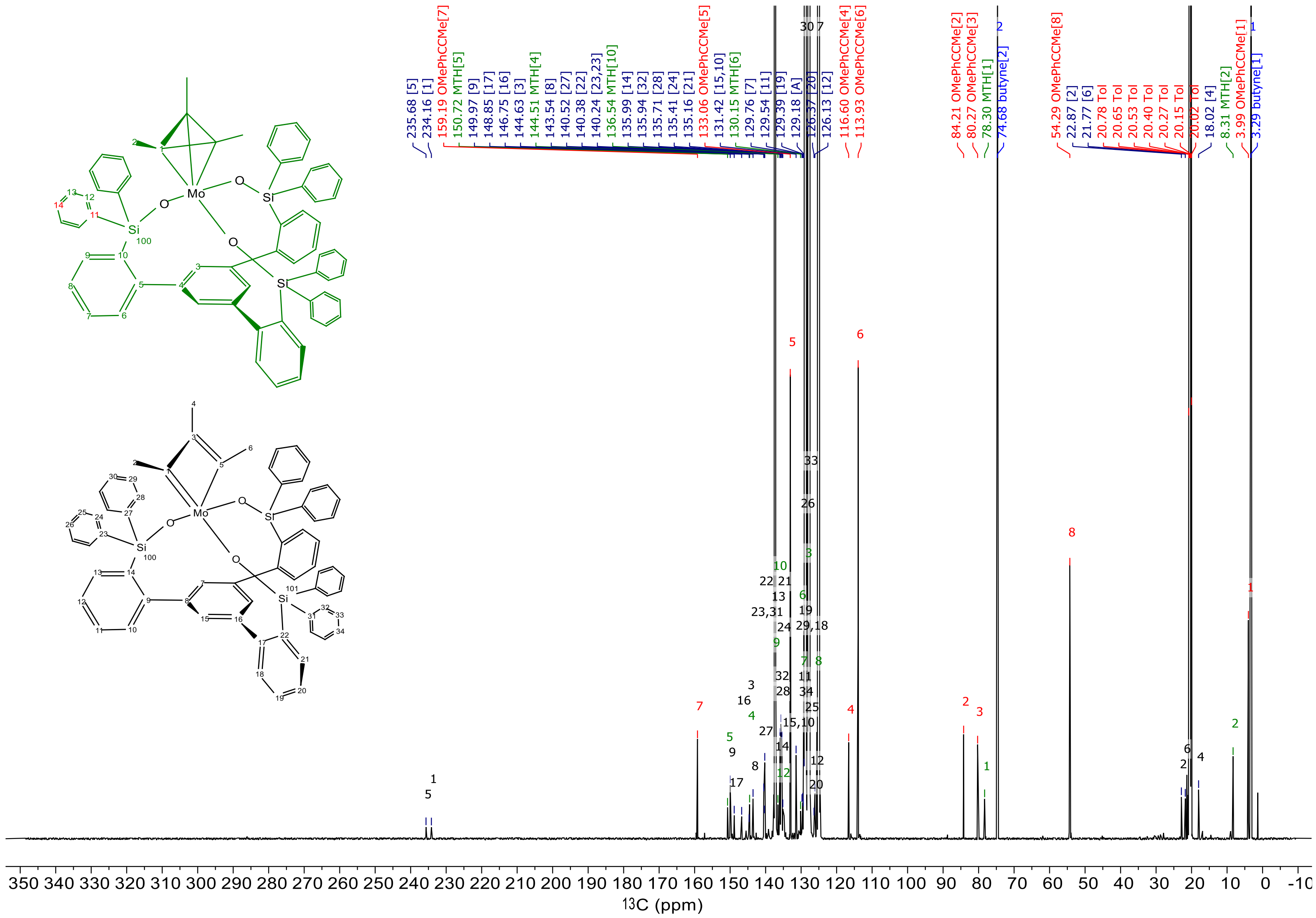

Extension of ^{13}C NMR Spectrum of Complexes 8 and 9 with the most relevant signals for the structure assignments, 151 MHz, $C_6D_5CD_3$, –40 °C

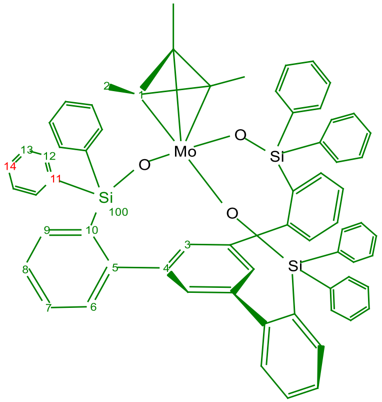

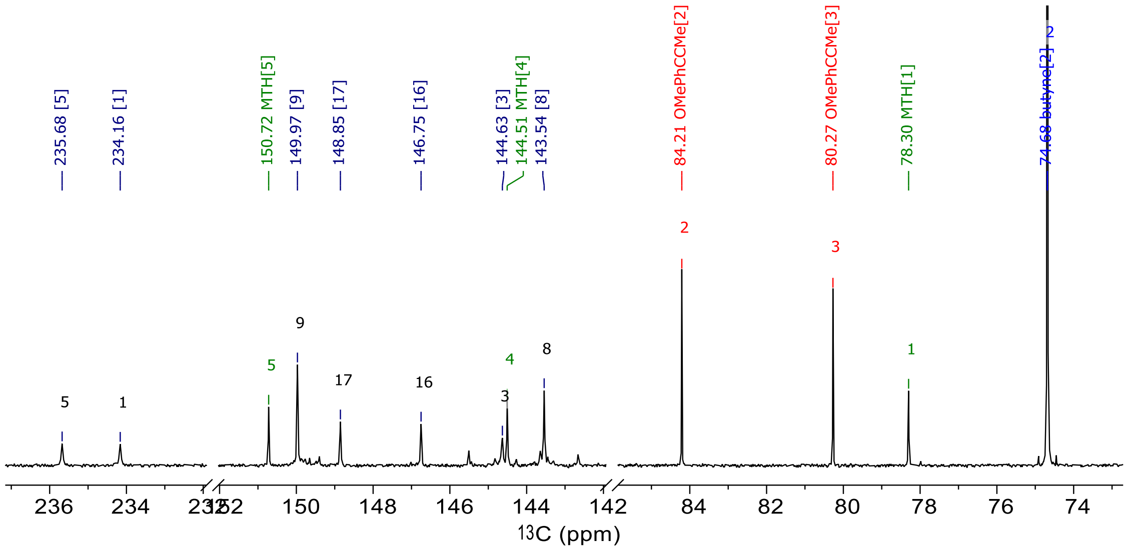

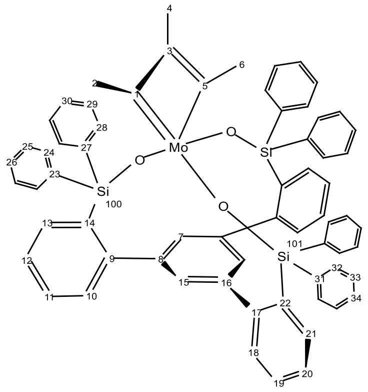

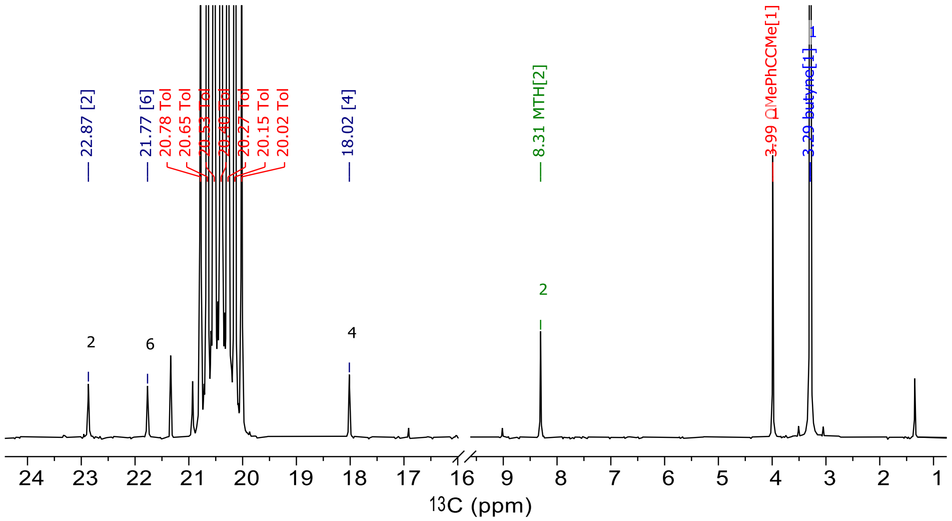

^{1}H-^{13}C edited HSQC NMR Spectrum of Complexes 8 and 9, $C_6D_5CD_3$, –40 °C

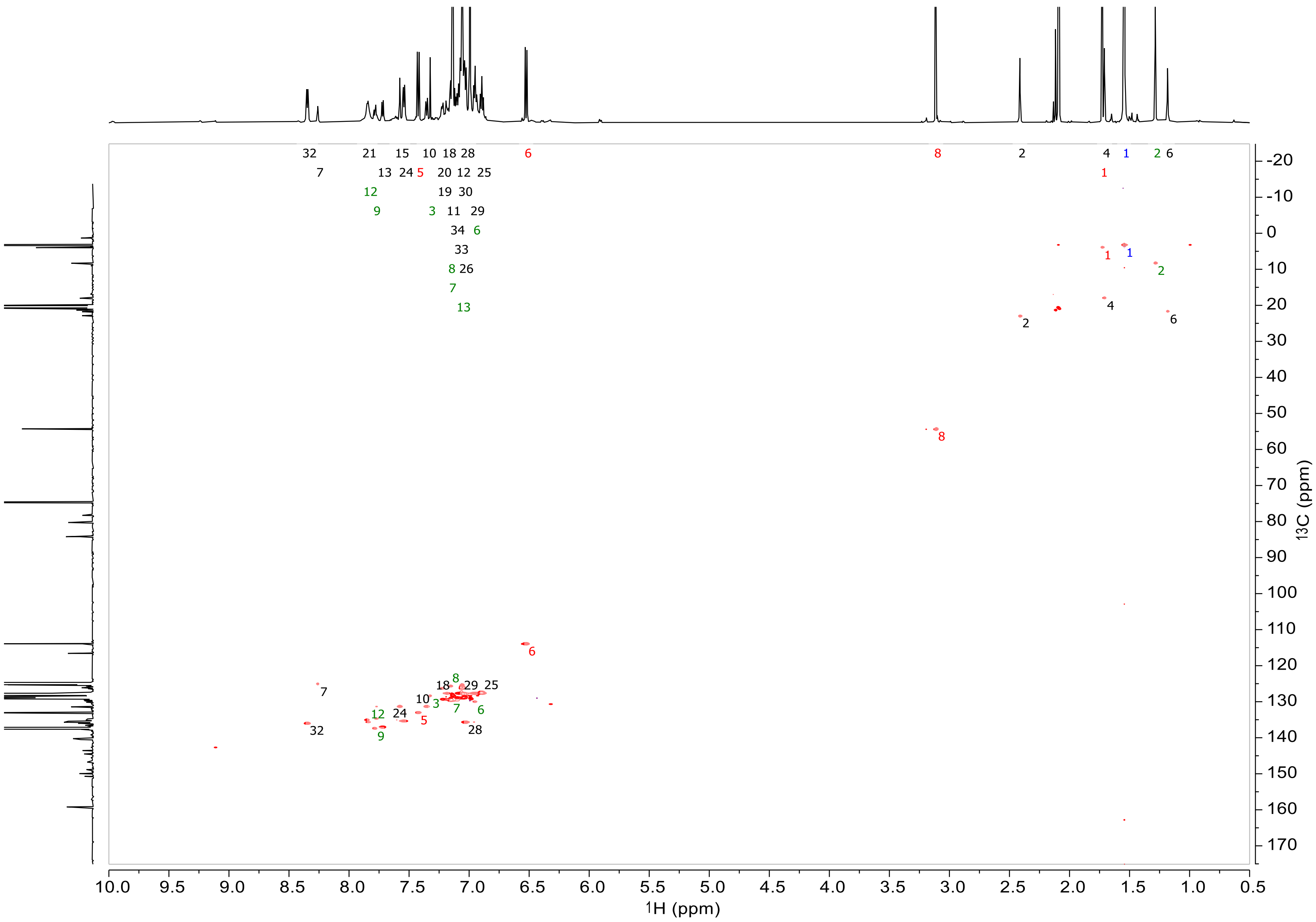

^{1}H-^{13}C HMBC NMR Spectrum of Complexes 8 and 9, $C_6D_5CD_3$, −40 °C

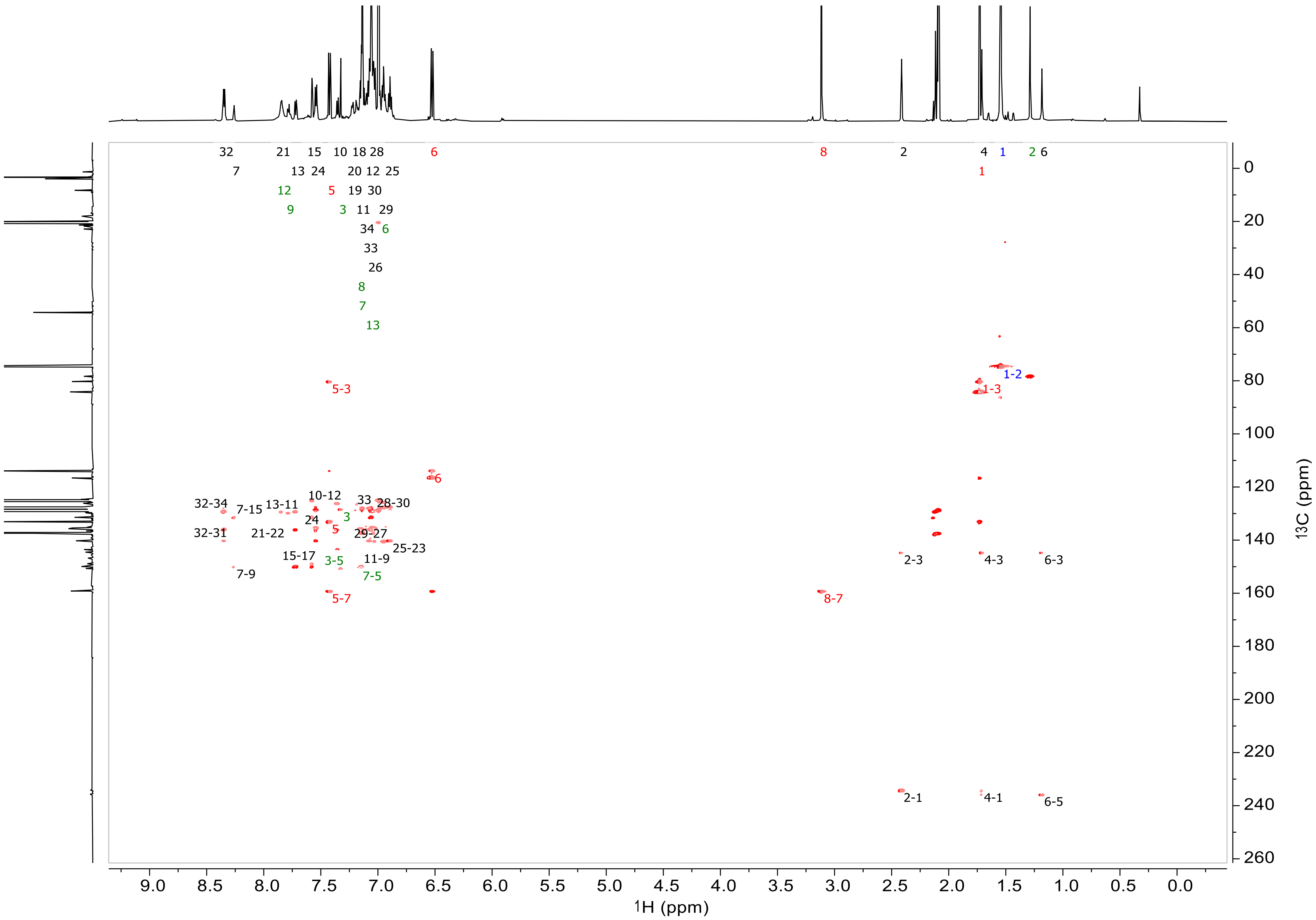

^{1}**H EASY-ROESY NMR Spectrum of Complexes 8 and 9**, 600 MHz, $\tau_{spinlock}$ = 200 ms, $C_6D_5CD_3$, –40 °C

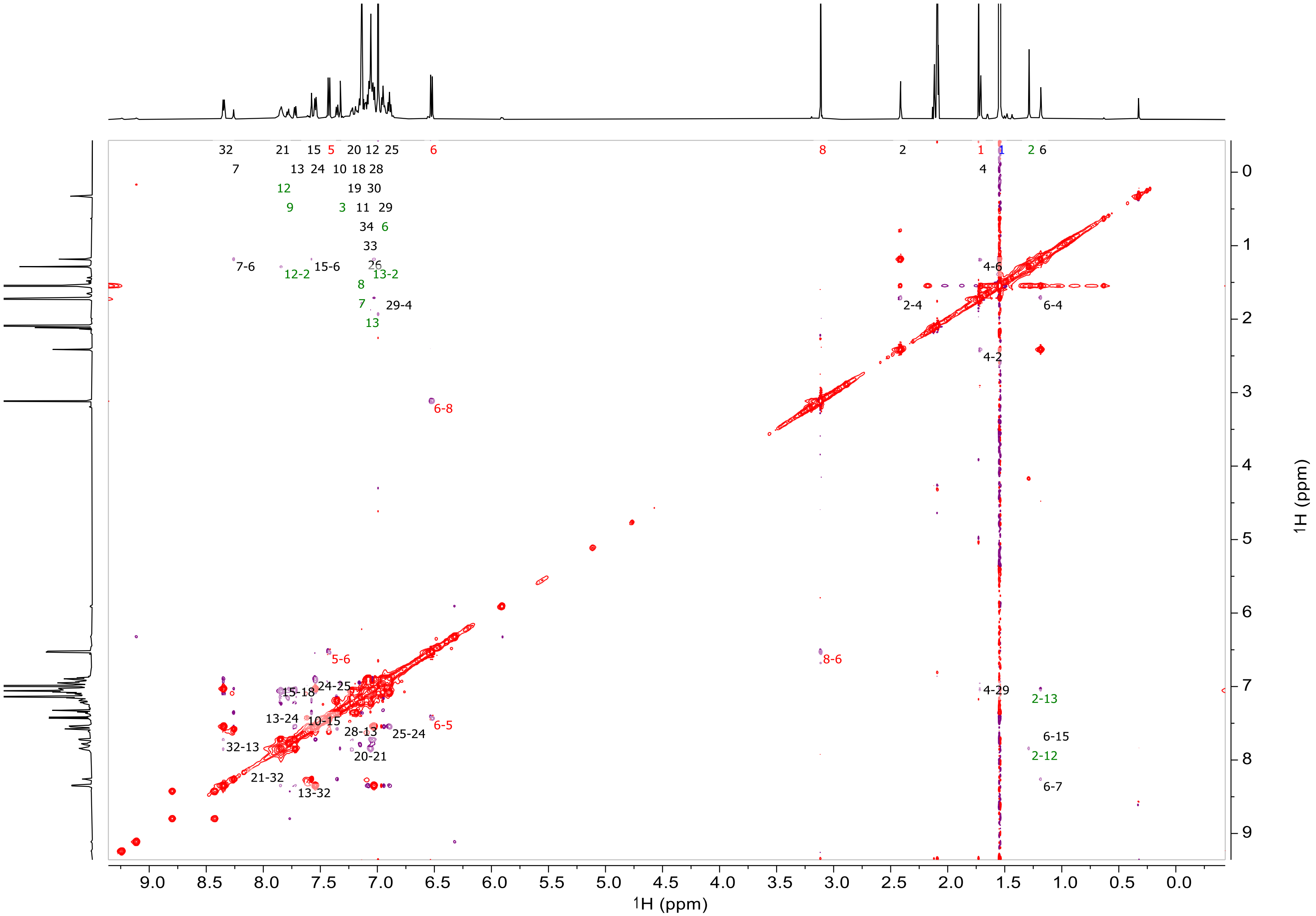

Aromatic region of the ^{1}H EASY ROESY NMR Spectrum of Complexes 8 and 9, 600 MHz, $\tau_{spinlock}$ = 200 ms , $C_6D_5CD_3$, –40 °C

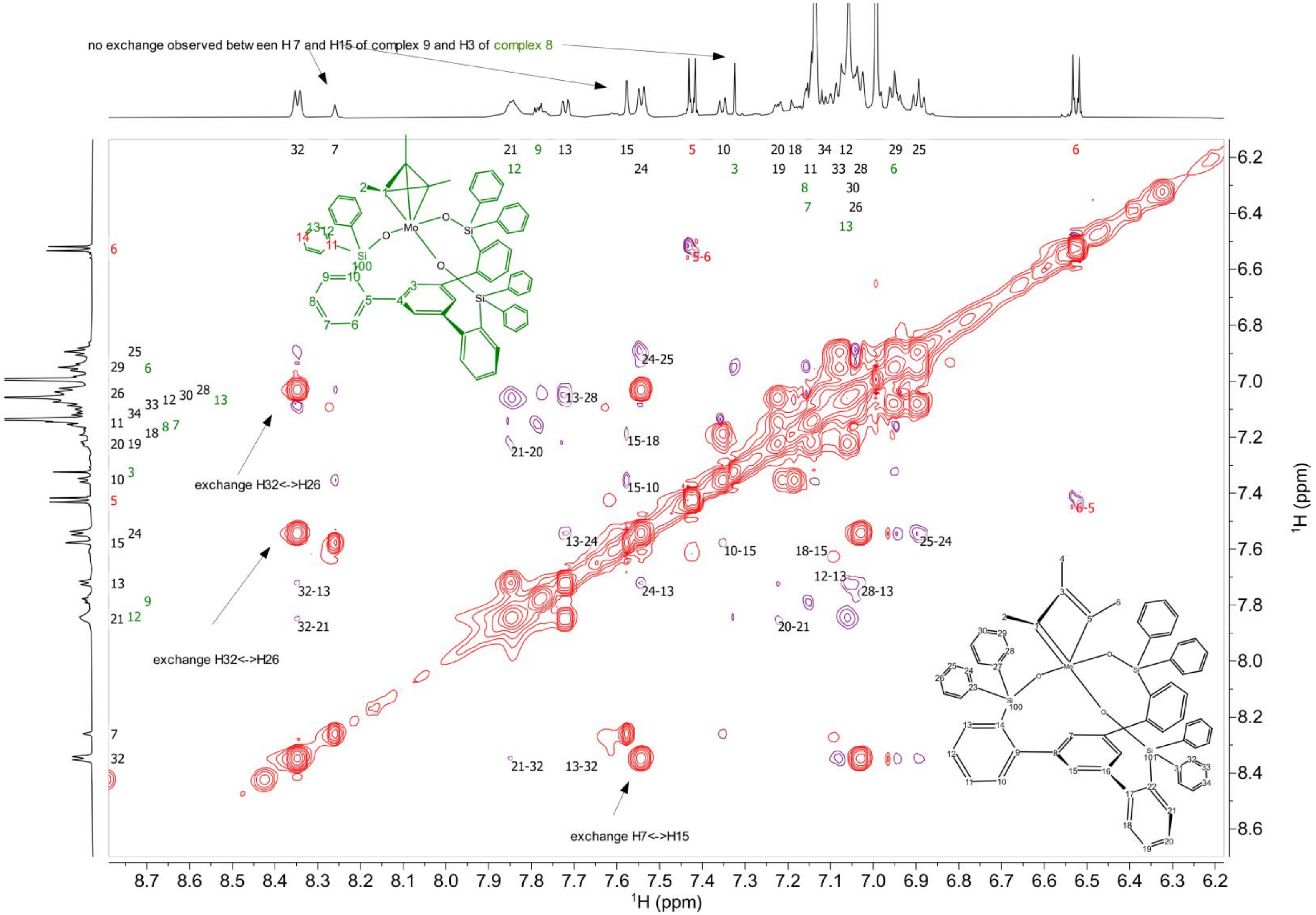

Aliphatic region of the ^{1}H EASY ROESY NMR Spectrum of Complexes 8 and 9, 600 MHz, $\tau_{spinlock}$ = 200 ms , $C_6D_5CD_3$, –40 °C

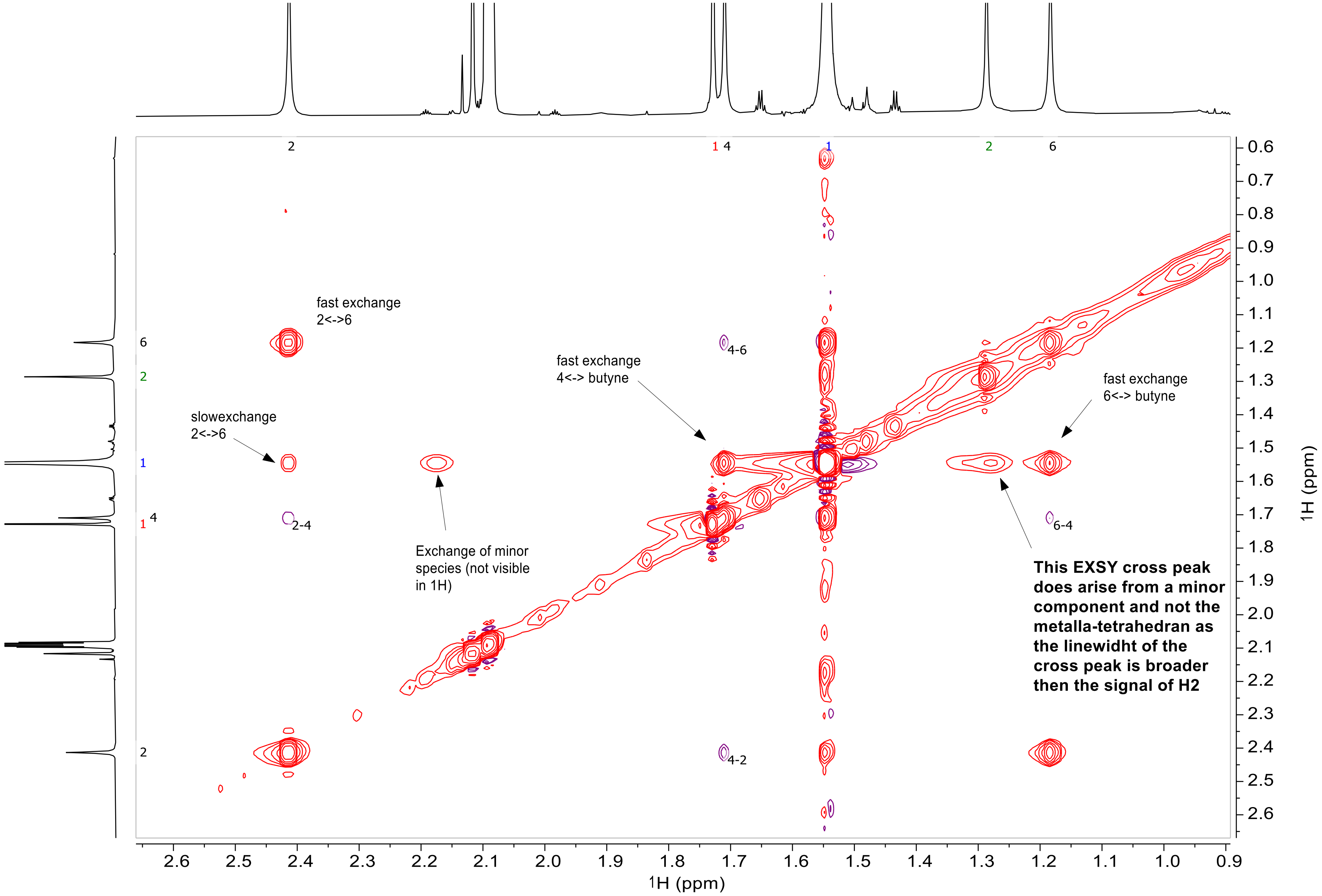

Aliphatic region of the ^{1}H EASY-ROESY NMR Spectrum of Complexes 8 and 9 with different thresholds, 600 MHz, $\tau_{spinlock}$ = 200 ms , $C_6D_5CD_3$, 0°C

0 °C

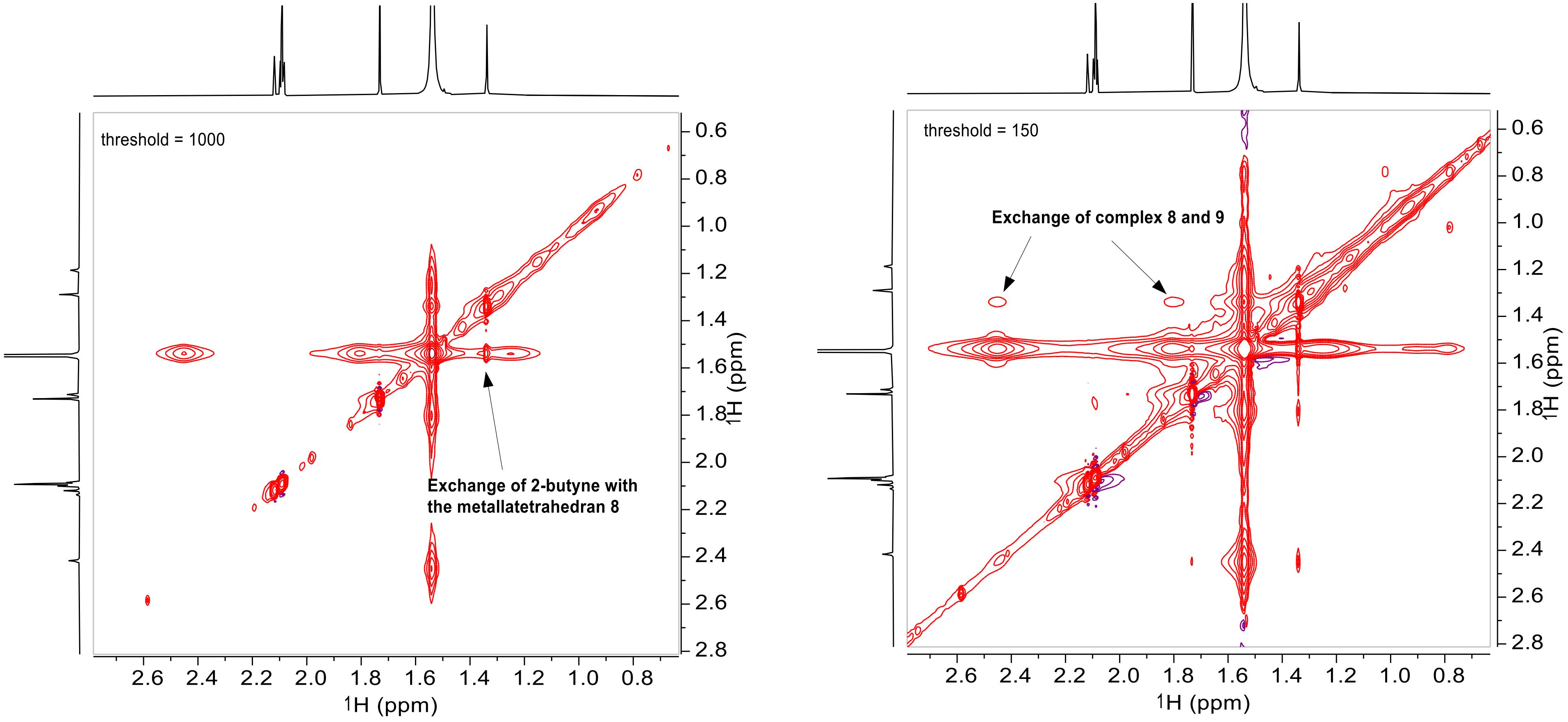

Comparison of EASY-ROESY NMR Spectra of Complexes 8 and 9 with different spinlock times and same threshold, 600 MHz, $C_6D_5CD_3$, –40 °C

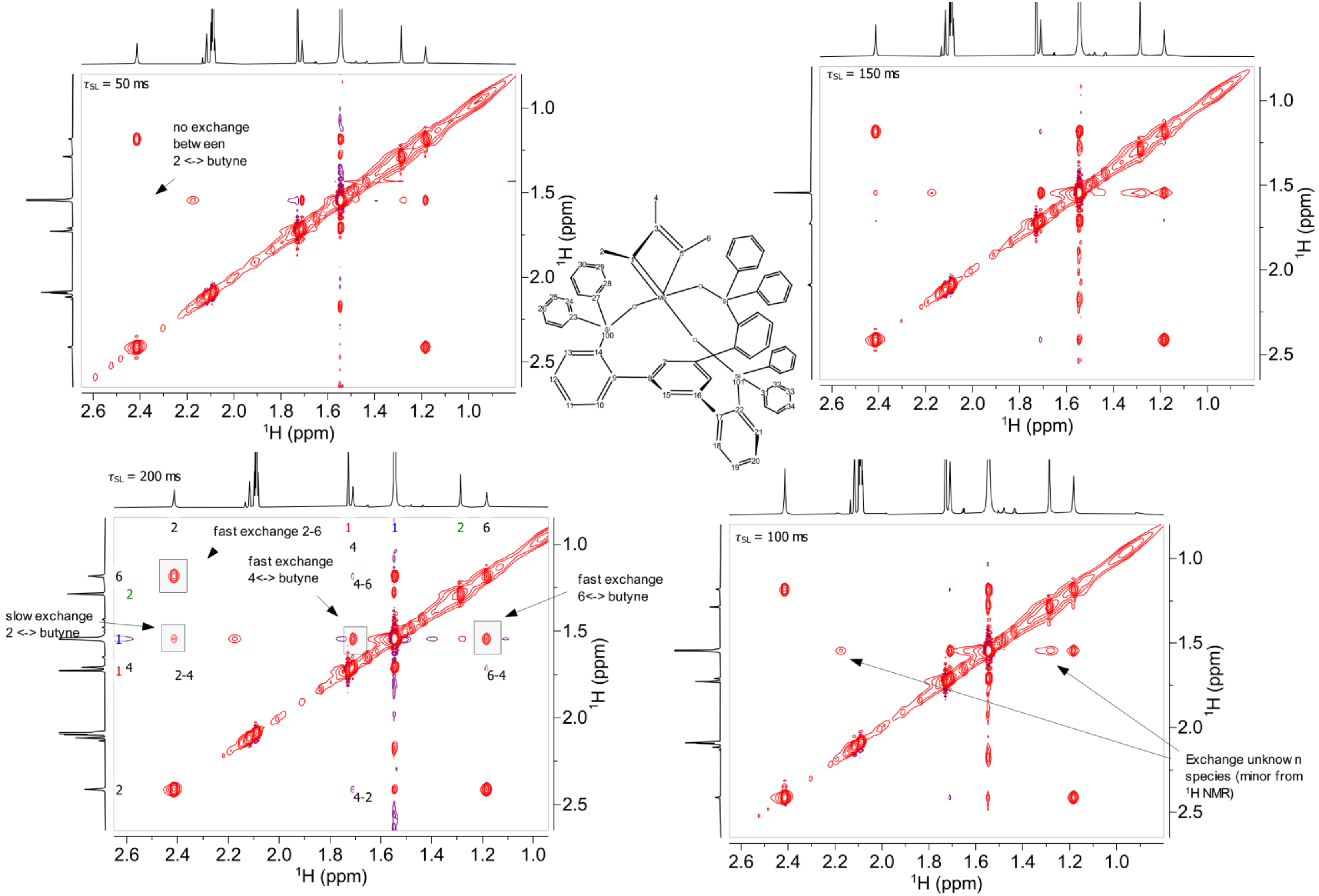

Cross-peak build up curves extracted from 2D EASY-ROESY NMR Spectra

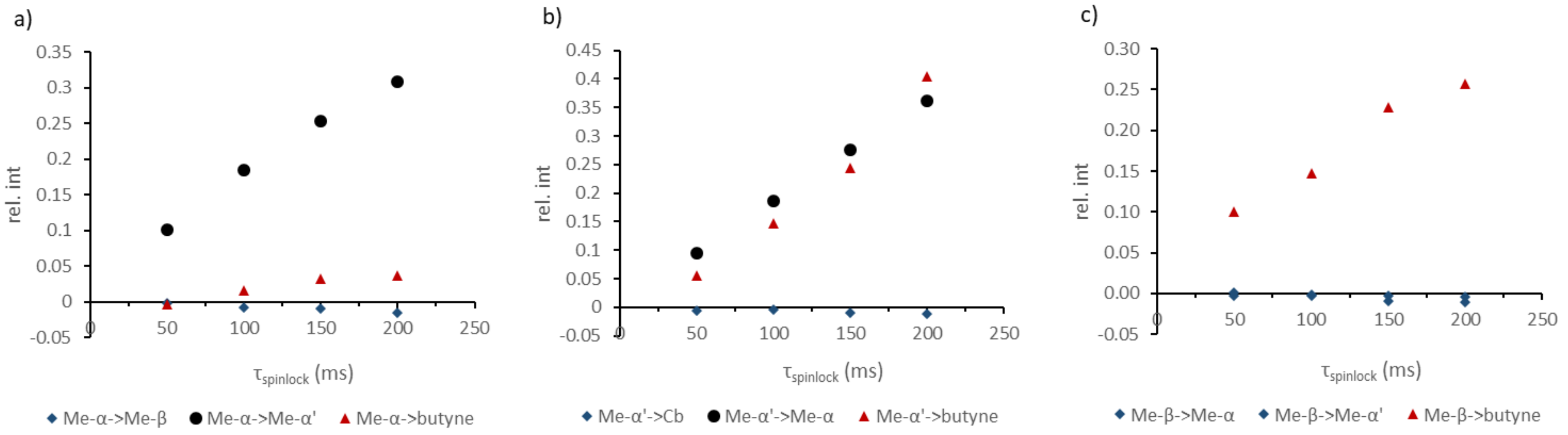

Figure 3: Normalized cross peak build-up curves extracted from the 2D EASY-ROESY spectra. Cross peaks were normalized to the diagonal cross peak integral of the corresponding diagonal peak. a) cross peak build up from the Me-group of compound 9 at Cα to the other methyl groups and 2-butyne; b) cross peak build up from the Me-group at Cα' to the other methyl groups and 2-butyne; c) cross peak build up from the Me-group at Cβ to the other methyl groups and 2-butyne.

^{29}Si-INEPT NMR Spectrum of Metallacyclobutadiene Complexes 8 and 9, 119 MHz, $C_6D_5CD_3$, –40 °C

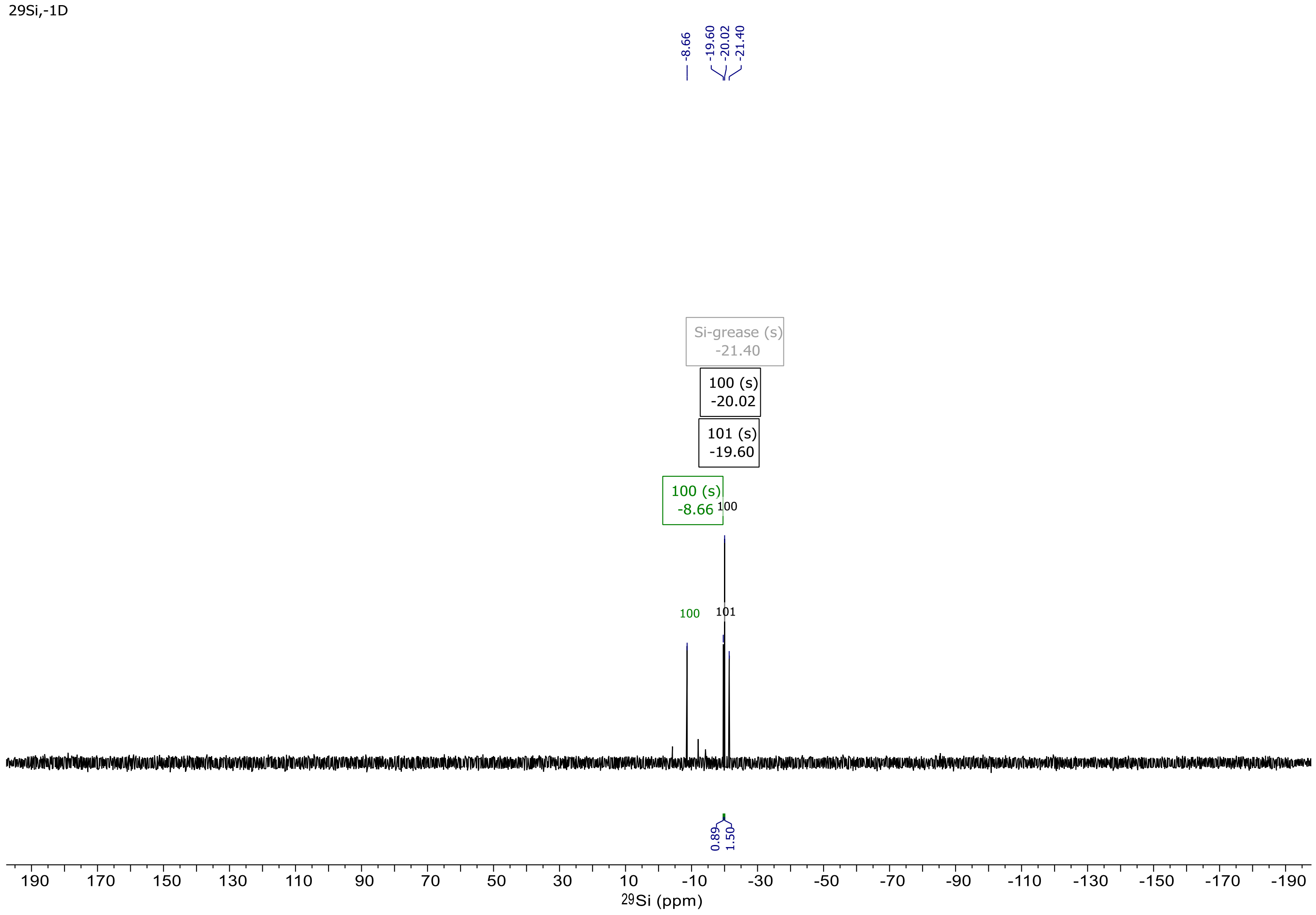

^{1}H-^{29}Si HMBC NMR Spectrum of Metallacyclobutadiene Complexes 8 and 9, $C_6D_5CD_3$, –40 °C

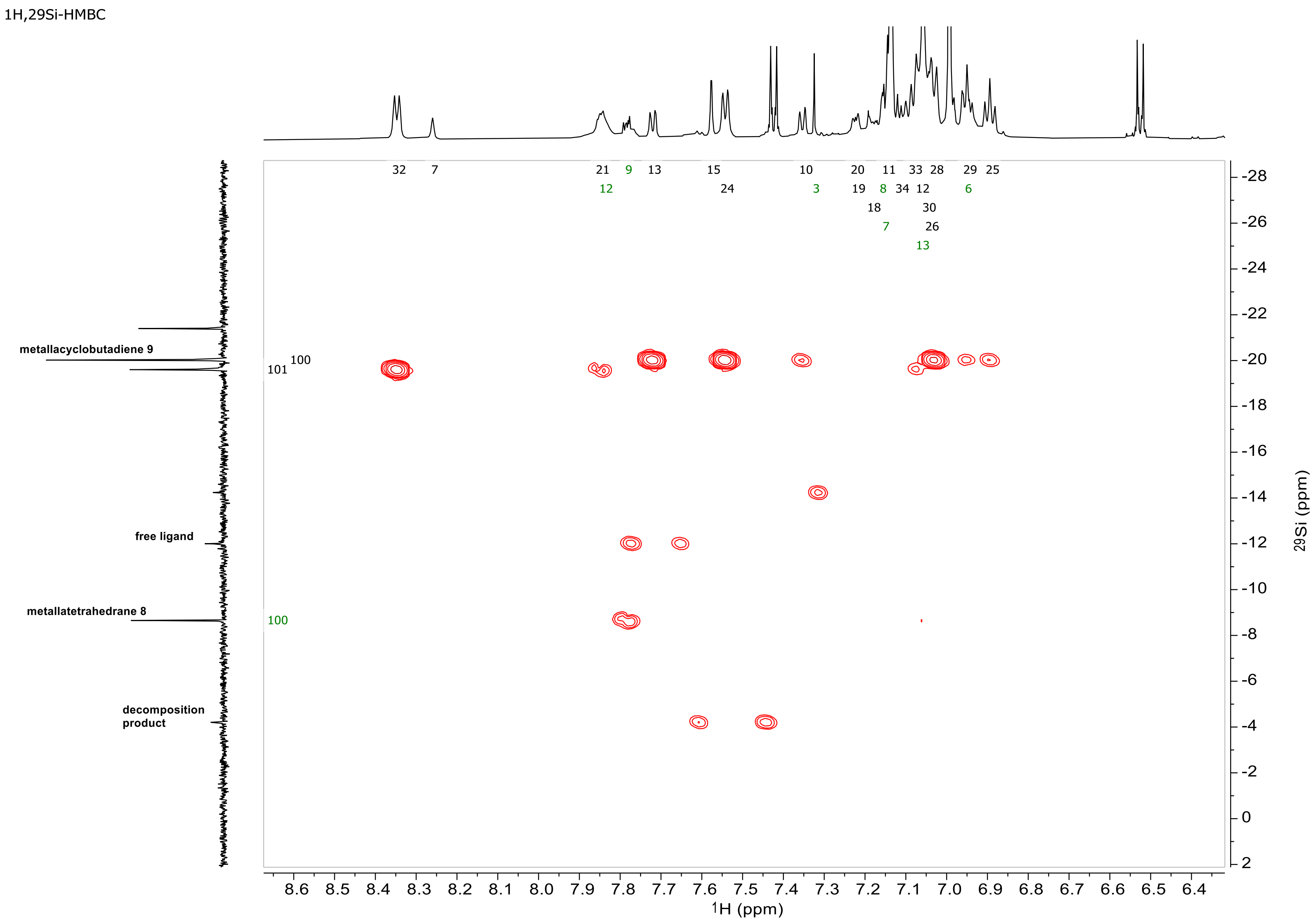

VT ^{29}Si NMR Studies of Complexes 8 and 9, 119 MHz, $C_6D_5CD_3$, 25 °C to –40 °C

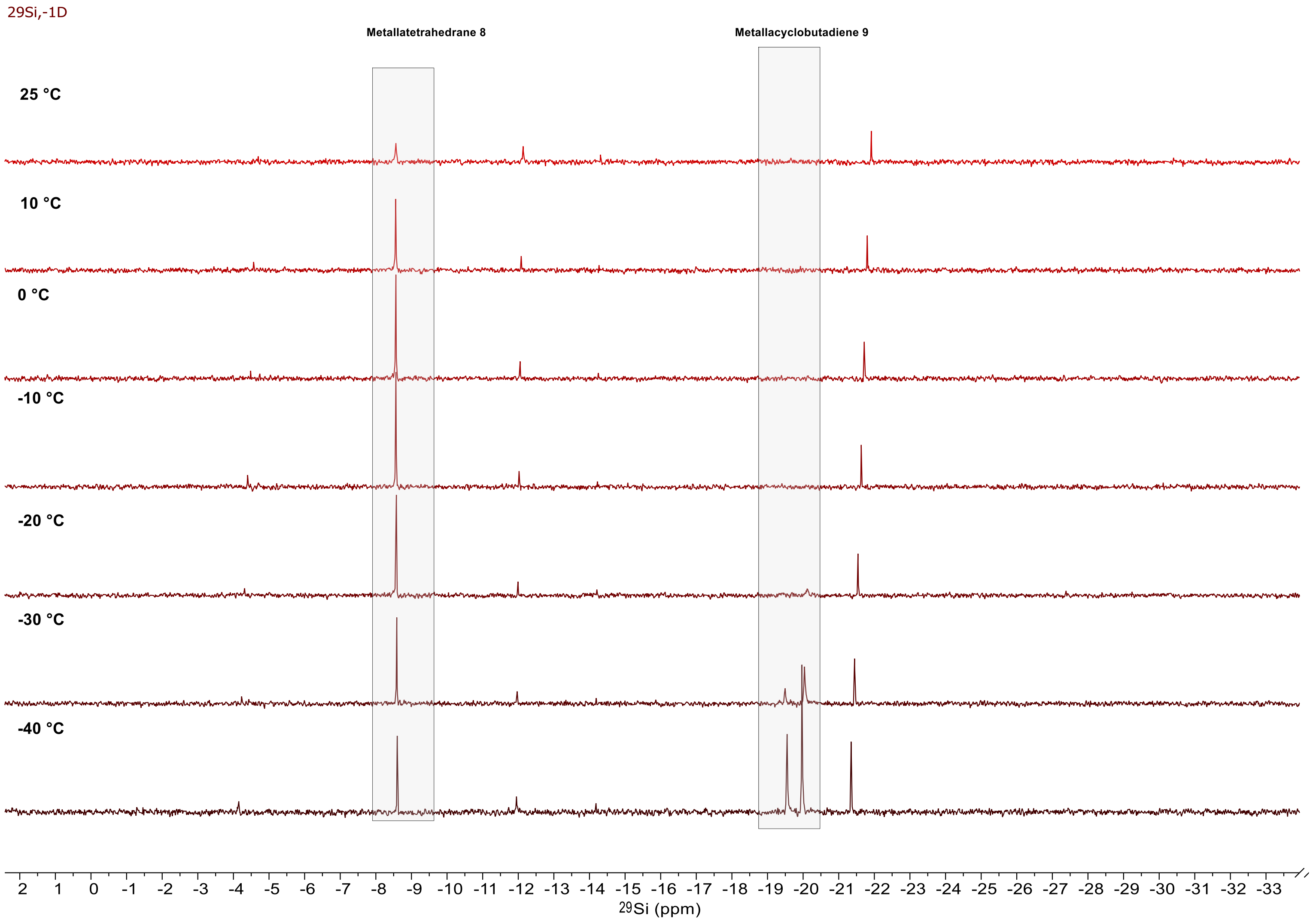

*The somewhat higher stability of the metallatetrahedrane **8** is in good agreement with the computed thermodynamic data (see Figure 4, main text)*

REFERENCES

1 (a) Hillenbrand, J.; Leutzsch, M.; Yiannakas, E.; Gordon, C.; Wille, C.; Nöthling, N.; Copéret, C.; Fürstner, A. "Canopy Catalysts" for Alkyne Metathesis: Molybdenum Alkylidyne Complexes with a Tripodal Ligand Framework. *J. Am. Chem. Soc*. **2020**, *142*, 11279-11294. (b) Hillenbrand, J.; Leutzsch, M.; Fürstner, A. Molybdenum Alkylidyne Complexes with Tripodal Silanolate Ligands: The Next Generation of Alkyne Metathesis Catalysts. *Angew. Chem. Int. Ed*. **2019**, *58*, 15690-15696. (c) Hillenbrand, J.; Leutzsch, M.; Gordon, C. P.; Copéret, C.; Fürstner, A. ^{183}W NMR Spectroscopy Guides the Search for Tungsten Alkylidyne Catalysts for Alkyne Metathesis. *Angew. Chemie Int. Ed.* **2020**, *59*, 21758–21768.

2 Harris, R. K.; Becker, E. D.; Cabral de Menezes, S. M.; Granger, P.; Hoffman, R. E.; Zilm, K. W., Further conventions for NMR shielding and chemical shifts (IUPAC Recommendations 2008). *Pure Appl. Chem.* **2008**, 80, 59.

3 Tao, J.; Perdew, J. P.; Staroverov, V. N.; Scuseria, G. E. Climbing the Density Functional Ladder: Nonempirical Meta–Generalized Gradient Approximation Designed for Molecules and Solids. *Phys. Rev. Lett.* **2003**, *91*, 146401.

4 (a) Grimme, S.; Ehrlich, S.; Goerigk, L. Effect of the Damping Function in Dispersion Corrected Density Functional Theory. *J. Comput. Chem.* **2011**, *32*, 1456–1465. (b) Grimme, S.; Antony, J.; Ehrlich, S.; Krieg, H. A Consistent and Accurate Ab Initio Parametrization of Density Functional Dispersion Correction (DFT-D) for the 94 Elements H-Pu. *J. Chem. Phys.* **2010**, *132*, 154104.

5 (a) Weigend, F.; Ahlrichs, R. Balanced Basis Sets of Split Valence, Triple Zeta Valence and Quadruple Zeta Valence Quality for H to Rn: Design and Assessment of Accuracy. *Phys. Chem. Chem. Phys.* **2005**, *7*, 3297–3305. (b) Weigend, F. Accurate Coulomb-Fitting Basis Sets for H to Rn. *Phys. Chem. Chem. Phys.* **2006**, *8*, 1057.

6 (a) H. Jónsson, G. Mills, and K. W. Jacobsen, Classical and Quantum Dynamics in Condensed Phase Simulations, edited by B. J. Berne, G. Ciccotti, and D. F. Coker (World Scientific, Singapore, **1998**), pp. 385–404 (b) Henkelman, G.; Jónsson, H. Improved Tangent Estimate in the Nudged Elastic Band Method for Finding Minimum Energy Paths and Saddle Points. *J. Chem. Phys.* **2000**, *113*, 9978–9985. (c) Henkelman, G.; Uberuaga, B. P.; Jónsson, H. A Climbing Image Nudged Elastic Band Method for Finding Saddle Points and Minimum Energy Paths. *J. Chem. Phys.* **2000**, *113*, 9901–9904.

7 (a) Becke, A. D. Density-Functional Thermochemistry. III. The Role of Exact Exchange. *J. Chem. Phys.* **1993**, *98* (7), 5648–5652. (b) Lee, C.; Yang, W.; Parr, R. G. Development of the Colle-Salvetti Correlation-Energy Formula into a Functional of the Electron Density. *Phys. Rev. B* **1988**, *37*, 785–789.

8 Garcia-Ratés, M.; Neese, F. Effect of the Solute Cavity on the Solvation Energy and Its Derivatives within the Framework of the Gaussian Charge Scheme. *J. Comput. Chem.* **2020**, *41*, 922–939.

9 (a) Neese, F. The ORCA Program System. *WIREs Comput. Mol. Sci.* **2012**, *2*, 73–78. (b) Neese, F. Software Update: The ORCA Program System, Version 4.0. *Wiley Interdiscip. Rev. Comput. Mol. Sci.* **2018**, *8*, e1327.
